# Chromosomal 3p loss and 8q gain drive vasculogenic mimicry via HIF-2α and VE-cadherin activation in uveal melanoma

**DOI:** 10.1038/s41418-025-01469-9

**Published:** 2025-02-26

**Authors:** Daniel Delgado-Bellido, Antonio Chacon-Barrado, Joaquin Olmedo-Pelayo, Carmen Jordán Perez, Paula Gilabert-Prieto, Juan Díaz-Martin, Angel Garcia-Diaz, F. J. Oliver, Enrique de Álava

**Affiliations:** 1 Instituto de Parasitología y Biomedicina López Neyra, CSIC, Granada, Spain; 2 Instituto de Salud Carlos III, CIBERONC, Madrid, Spain; 3 Institute of Biomedicine of Sevilla, IBiS/ Virgen del Rocio University Hospital /CSIC/University of Sevilla/CIBERONC, 41013 Seville, Spain; 4 Department of Normal and Pathological Cytology and Histology, School of Medicine, University of Seville, 41009 Seville, Spain

**Keywords:** Metastasis, Cadherins, Focal adhesion

## Abstract

Uveal melanoma (UM) is the most common primary intraocular malignant tumor in adults and is where Vasculogenic Mimicry (VM) was first described. VM enables aggressive cancer cells to independently form blood networks, complicating treatment for patients exhibiting VM. Previous studies linked VE-Cadherin phosphorylation at Y658 to gene expression via Focal Adhesion Kinase (FAK), enhancing the Kaiso/β-catenin/TCF-4 complex associated with VE-Cadherin and thereby promoting VM. Recently, an allosteric HIF-2α inhibitor (Belzutifan) was FDA-approved for VHL-associated ccRCCs. In this research, we elucidate the primary causes of VM formation in UM patients with chromosome 3p loss and chromosome 8q gain, identifying VHL, BAP1, and FAK as important factors driving VM and worsening prognosis. These factors promote abnormal activation of HIF-2α and VE-Cadherin under basal hypoxic conditions, leading to VM formation. Cytoscan 750k experiments on the MUM 2B cell line reveal a loss of chromosome 3p, where the VHL, BAP1, and CTNNB1 genes are located, and a gain of chromosome 8q (FAK), whereas the MUM 2C cell line shows a gain of chromosome 3p. This provides an outstanding cross-sectional model from patient samples to established cell lines for VM studies. LC-MS experiments demonstrate that VE-Cad/ENG expression is related to FAK activity in UM cell lines. Finally, using a combination of Belzutifan (HIF-2α inhibitor) and FAK inhibitor (FAKi), we observed a significant reduction in UM xenografts. Our results lead us to propose combining Belzutifan and FAKi as a personalized treatment strategy for UM patients. This approach inhibits VM formation and counters the initial hypoxic conditions resulting from chromosome 3p loss and chromosome 8q gain in UM patients, instilling confidence in the potential of this treatment strategy.

## Introduction

Uveal melanoma (UM) is the most common primary intraocular malignant tumor in adults. Incidence rates were generally ≥8.0 cases per million person-years in Northern Europe, Western Europe, and Oceania; 2.0–7.9 in North America, Eastern Europe, and Southern Europe; and <2.0 in South America, Asia, and Africa [1], classifying it as a rare and neglected disease [2]. UM primarily originates from melanocytes in the choroid (90%), with fewer cases in the iris (4%) and ciliary body (6%). The uveal tract, composed of the retinal choroid, iris, and ciliary body, is a vascular tissue without lymphatic vessels. Therefore, angiogenesis, rather than lymphangiogenesis, is crucial for UM metastasis [3]. Unlike UM, which can metastasize to various organs, UM metastases are almost exclusively found in the liver (over 90% of cases), bypassing the typical TNM staging progression. This distinct metastatic pattern makes UM a simpler model for studying and identifying potential treatments [4]. Approximately 50% of UM cases develop metastases, with a 40% mortality rate from metastatic disease despite successful primary tumor treatment [5]. Metastasis primarily affects the liver in up to 90% of cases [6, 7]. Most UMs (80–90%) have mutations in the GNAQ gene, leading to constitutively active Gαq proteins that act as oncogenes, causing imbalances in effector kinases like pFAK [8]. The cytogenetic and molecular genetic characteristics of metastases are likely not only associated with but may also directly influence the progression of metastatic uveal melanoma. However, their impact on overall survival (OS) has been addressed only preliminary. Cytogenetic abnormalities, particularly partial or complete monosomy 3 [9], and pathogenic variants in BAP1 [10], demonstrated by loss of nuclear immunoreactivity for the BAP1 protein, are detected in 70–100% and 60–80% of metastatic uveal melanoma cases, respectively. Aberrant tumor microcirculation, a critical factor in hypoxia and treatment failure, is influenced by abnormal angiogenesis termed Vasculogenic Mimicry or Vascular Mimicry (VM) [11, 12]. VM is the dominant blood supply in early melanoma growth, later supported by conventional blood vessels. VM not only aids tumor growth but also increases the likelihood of metastasis by enhancing tumor cells’ proximity to blood flow [13, 14]. VM pathways, formed by highly invasive, genetically deregulated tumor cells, were first described in UM cells [15]. VM facilitates tumor perfusion and may help disseminate tumor cells through blood vessels. There is a strong association between VM patterns in primary uveal and cutaneous melanoma and death from metastasis [16]. Aberrant extra-vascular expression of VE-cadherin, specifically activated by FAK at Y658, has been observed in cancers associated with VM [17–19]. Our group reported high expression of pVE-cadherin Y658 in malignant melanoma, forming a complex with p120-catenin and Kaiso in the nucleus. UM cells lacking VE-cadherin (via CRISPR/Cas9 or siRNA) lose the ability to form VM [20]. Additionally, p120 catenin stabilizes VE-cadherin in VM-prone melanoma cells through VE-PTP involvement [21]. In fact, β-catenin/TCF-4 associates with nuclear VE-cadherin, enhancing the ability of melanoma cells to undergo VM. Preventing VE-cadherin phosphorylation at Y658 disrupts the VE-cadherin/β-catenin complex, increasing β-catenin degradation and reducing TCF-4-dependent gene transcription. Uveal melanoma cells lacking VE-cadherin lose β-catenin expression, but restoring VE-cadherin stabilizes β-catenin and reduces tumor growth. Combined treatment with FAK inhibitor PF-271 and anti-angiogenic agent bevacizumab significantly reduces tumor growth. VE-cadherin’s abnormal expression and phosphorylation by FAK in metastatic melanoma cells promote the aggressive VM phenotype through β-catenin cooperation and enhanced TCF-4-dependent transcription [22].

Hypoxia, characterized by low oxygen levels, is a defining feature of the tumor microenvironment and plays a significant role in driving cancer progression and resistance to treatment [23]. Extensive research has emphasized the pivotal influence of hypoxia on controlling tumor invasiveness, angiogenesis, VM, and treatment response in melanoma [12]. The VHL tumor suppressor gene, identified in 1993, is essential for cellular oxygen sensing by targeting hypoxia-inducible factors. Inactivation of VHL is common in clear-cell renal cell carcinoma (ccRCC), leading to targeted therapies [24]. Recent studies reveal additional driver genes in ccRCC pathogenesis, highlighting ongoing research into VHL biology and potential new treatments (Belzutifan PT2977, FDA approved in 2021 [25–27], but the VHL role in UM hasn’t been explored yet. Following the initial findings of VM in UM, several other research groups have corroborated its role in UM. While hypoxia exposure has been implicated as a factor in VM in tumors of various origins, there is currently no available data regarding the regulation of VM by hypoxia/VHL in UM.

## Results

### Critical role of chromosome 3 loss and 8 gain in uveal melanoma patient outcomes

UM [28] is a rare cancer but is the second most common type of melanoma after cutaneous melanoma and the most common primary intraocular malignancy. The most frequent structural alteration linked to a higher risk of distant recurrences in UM is complete monosomy of chromosome 3. Other cytogenetic abnormalities include 8q gain, and losses of 1p, 6q, and 8p [29].

**Fig. 1:**
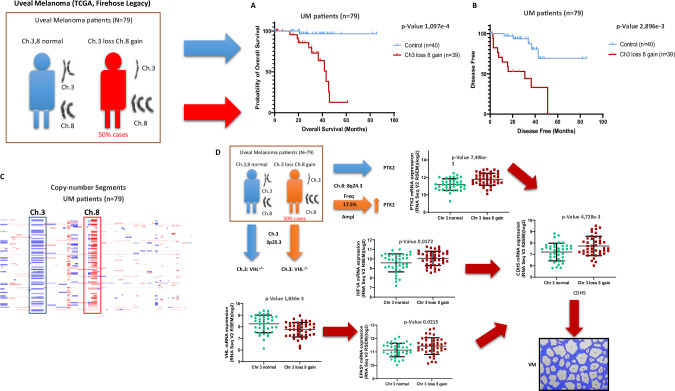
Critical role of chromosome 3 loss and 8 gain in uveal melanoma patient outcomes. **A** Overall survival and **B** disease free in Ch3,8 normal vs Ch3 loss 8 gain uveal melanoma patients. **C** Copy number alterations in *n* = 79 cohort’s uveal melanoma patients. **D** Evaluation of the correlation between Ch3,8 normal vs Ch3 loss 8 gain uveal melanoma patients and CDH5, VHL, EPAS1, HIF-1A and PTK2 mRNA levels in a cohort of uveal melanoma (*n*  =  79, TCGA Firehose legacy) using cBioPortal database.

With this background and aims, we explored the potential implications of chromosome 3 loss and chromosome 8 gain on survival and disease progression in uveal melanoma patients using the cBioPortal database (UM *n* = 79 patients). We confirmed that patients with these chromosomal aberrations have a statistically significant reduced survival of only 60 months compared to the control group (Fig. 1A), as well as a more rapid disease progression (Fig. 1B). Investigating the copy number alterations (CNAs), we observed that half of the patients exhibit these previously mentioned chromosomal aberrations (Fig. 1C). We investigated the implications of chromosome 3 loss in uveal melanoma. VHL, located on chromosome 3p25.3, could be reduced due to this loss. PTK2 (FAK), on chromosome 8q24.3, shows a 17.5% amplification frequency, suggesting increased expression due to chromosome 8 gain or amplification. We explored the effects of reduced VHL and increased PTK2 in this patient cohort. To investigate this (Fig. 1D), we compared mRNA expressions between groups and observed that patients with chromosome 3 loss and chromosome 8 gain indeed have statistically lower expression of VHL. As reported in numerous scientific articles [24, 30, 31], the implication of VHL in regulating HIFs levels was evident, with both HIF-1α and EPAS1 (HIF-2α) levels highly increased in these patients. This led to an increase in a classical endothelial marker and EPAS1 target, VE-cadherin, which is closely associated as a marker of VM. Additionally, we observed that these patients also have increased mRNA expression of PTK2. Combining these chromosomal aberrations, we believe, as we have reported in recent years [17, 20–22], that these chromosomal abnormalities could culminate in VM undesired production. As shown in Supplementary Fig. S1A, these uveal melanoma patients exhibit an increase in the expression of classic and well-documented hypoxia-dependent genes such as PDGFB, LOXL2 [32], VEGF, ANGPT2, KDR, and S1PR1 [22, 33]. Markers of EMT and VM, such as TWIST1 [22] and FOXC2, are also elevated. Recent publications have highlighted the involvement of FOXC2 in VM and its role in increased resistance to anti-angiogenic treatments [34]. Interestingly, there is a decrease in mRNA expression of HIF1AN (FIH) in these patients, which is a known inhibitor of HIFs expression [35]. Additionally, these patients also overexpress FAK activity markers at the mRNA level, such as the genes CCL5, CXCL10 [36], and CCN2 [8] from the YAP/TAZ pathway (Supplementary Fig. S1B).

### Origins of vasculogenic mimicry

VM was intriguingly first described in metastatic cells originating from uveal melanoma [15]. Initially, this phenomenon was considered an adaptation by highly aggressive tumor cells to express endothelial markers. Since its discovery, VM has been a subject of controversy, but over the past decade, it has revealed a novel angiogenic dynamic that may explain the failure of anti-angiogenic treatments (anti-VEGF) [22, 34, 37, 38]. One of the markers initially studied was the aberrant expression of VE-Cadherin by uveal melanoma cells [39]. Over time, the study of vasculogenic mimicry has extended to other tumor models [18, 40].

**Fig. 2:**
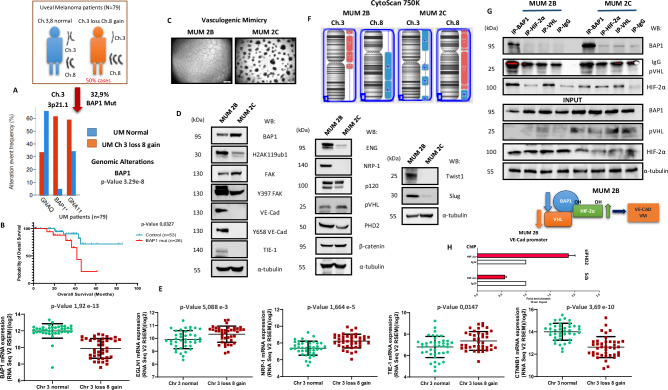
Origins of vasculogenic mimicry. **A** Alteration event frequency in UM patients Ch3,8 normal vs Ch3 loss 8 gain, denoted the BAP1 high altered event in Ch3 loss 8 gain patient and **B** Analysis of BAP1 mRNA expression (*n* = 79, TCGA Firehose Legacy) using the cBioPortal database and overall survival comparison between Control and BAP1-mutant patients. **C** Vasculogenic Mimicry formation in matrigel from MUM 2B and MUM 2C cells. **D** Western blot experiments in MUM 2B and MUM 2C cells. **E** Evaluation of the correlation between Ch3,8 normal vs Ch3 loss 8 gain uveal melanoma patients and EGLN, NRP-1, TIE1 and CTNNB1 mRNA levels in a cohort of uveal melanoma (*n*  =  79, TCGA Firehose legacy) using cBioPortal database. **F** Ch3 and Ch8 representation from Cytoscan 750 K experiments in MUM 2B and MUM 2C cells line. **G** Immunoprecipitation of BAP1, HIF-2α and VHL experiments in MUM 2B cells, schematic figure represented the BAP1/HIF-2α pathway in VM implications. **H** ChIP-HIF-2α assay in MUM 2B cells at the VE-Cad promoter with and without PHD2.

BAP1, a deubiquitinase with a ubiquitin C-terminal hydrolase domain, plays diverse biological roles and has been linked to human cancer through advanced sequencing studies. Somatic and germline mutations in the BAP1 gene are prevalent in mesothelioma, uveal melanoma [41], and clear cell renal cell carcinoma. The BAP1 cancer syndrome shows that individuals with inherited BAP1 mutations often develop multiple cancers with high penetrance. Aberrant deposition of H2AK119ub1 is strongly linked to the development of several human cancers. This is notably due to BAP1 loss causing global increases in H2AK119ub1, which occurs frequently in uveal melanoma (~45%) [42–44]. This suggests that BAP1 acts as a tumor suppressor, though its mechanisms are still being understood. With this aim, we decided to investigate the possible implications of BAP1 in the Ch3 loss 8 gain patients (Fig. 2). We found that 32.9% of the patients have a BAP1 DeepDel mutation, with significant alteration events occurring at a frequency of around 60%. This is not surprising because BAP1 is located on the 3p21.1 arm of chromosome 3. This DeepDel mutation of BAP1 in these patients results in a decreased mRNA expression of BAP1 and significantly reduced overall survival (Fig. 2B). As previously mentioned, the initial studies on VM were conducted in highly aggressive metastatic uveal melanoma cells with different gene expression profiling [13, 45], specifically in cells called MUM 2B and MUM 2C, which were extracted from the same patient. As extensively studied, these cells show that MUM 2B cells can form vascular structures independently, unlike the MUM 2C cell line (Fig. 2C). We decided to investigate the chromosomal fingerprint in these UM VM models. Using Western blotting experiments (Fig. 2D), we observed that MUM 2C cells exhibit higher protein expression levels of BAP1, pVHL, and β-catenin. Interestingly, all three genes are localized on chromosome 3p, suggesting that MUM 2B cells may have an aberrant loss of chromosome 3. Moreover, the MUM 2B cell line exhibits high expression of H2AK119ub1 by BAP1 loss expression, FAK Y397 [46], which is implicated in the increased kinase activity of FAK, as well as VE-Cad, its phosphorylation at Y658 [20], TIE-1 [39, 47], ENG [48], NRP-1 [49], PHD2, TWIST1 [22], and Slug [50], all related in VM formation and positivity. This strongly suggests that the MUM 2B cell line may have a chromosomal aberration involving the loss of chromosome 3 and the gain of chromosome 8, similar to what is observed in 50% of uveal melanoma patients in mRNA expression levels (Fig. 2E). The next step was to investigate the possible chromosomal aberrations in both cell lines (MUM 2B vs. MUM 2C) through CytoScan 750 experiments (Fig. 2F). This confirmed that MUM 2B has a loss of the short arm of chromosome 3, where the VHL, BAP1, and CTNNB1 genes are located and gain of the long arm chromosome 8 (PTK2). Surprisingly, the MUM 2C cell line has a short arm gain of chromosome 3 (VHL, BAP1 and CTNNB1). Thus, providing the best cross-sectional model from patient samples to established cell lines for VM models. As recently investigated, BAP1 binds to HIF-1α [51], thus regulating its activity. Therefore, we decided to explore the potential interaction of BAP1 in the complex dynamics with pVHL and HIF-2α (Fig. 2G). These immunoprecipitation experiments revealed that this trio of proteins (BAP1, pVHL, and HIF-2α) come together to promote high expression of HIF-2α and its activity on VM markers, such as the high expression of VE-Cad by MUM 2B cell lines even without hypoxic conditions (basal chromosome 3 loss). This is partially attributed to the loss of VHL/BAP1 expression in the MUM 2B cell line. It is hypothesized that the interplay between VHL, BAP1, and HIF-2α at the basal chromosomal level acts as a regulator of HIF-2α expression, thereby sustaining VE-Cad gene expression in MUM 2B cells. In contrast, this regulatory mechanism is absent in MUM 2C cells, due to increased levels of BAP1 and VHL, which lead to a decrease in HIF-2 alpha expression, ultimately resulting in the lack of VE-Cad expression (Fig. 2D, G). PHD2 (prolyl hydroxylase domain 2) is considered to be the key oxygen sensor, as the knockdown of PHD2 results in elevated HIFs protein. Several recent studies have highlighted the importance of PHD2 in tumourigenesis [52]. Finally, we conducted ChIP-HIF-2α (Fig. 2H) experiments following siPHD2 to assess the potential implication of HIF-2α in VE-Cad promoter transcription, and this silencing increased VE-Cad expression.

### Depletion of VHL/PHD2 enhances p-FAK and VE-Cadherin Y658 phosphorylation, promoting vasculogenic mimicry

**Fig. 3:**
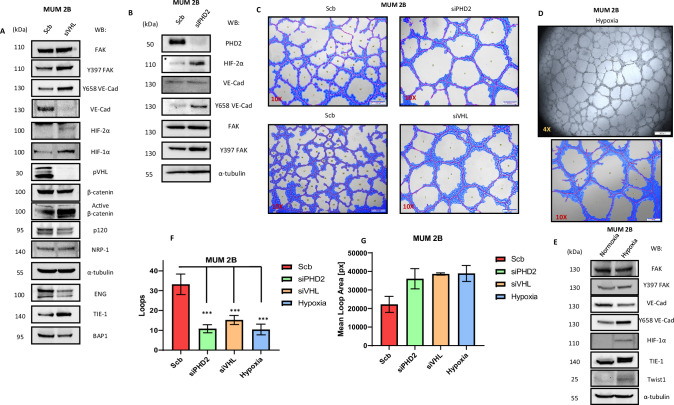
Depletion of VHL/PHD2 enhances p-FAK and VE-Cadherin Y658 phosphorylation, promoting vasculogenic mimicry. **A** Western blot experiments in MUM 2B after siVHL. **B** siPHD2. **C** In vitro angiogenesis assay with Matrigel in MUM 2B showed the effect of siVHL or siPHD2, **D** In vitro angiogenesis assay with Matrigel in MUM 2B in hypoxia (3%) conditions. **E** Western blot experiments in MUM 2B and MUM 2C in hypoxia conditions. **F**, **G** images were acquired using an Olympus CKX41 microscope (10X or 4X lens) (bars 50 μm) and the formation of tube-like structures was then quantified by Wimasis program. **D** In vitro angiogenesis assay with Matrigel in MUM 2C. **E** Western blot experiments in MUM 2C after siVHL.

**Fig. 4:**
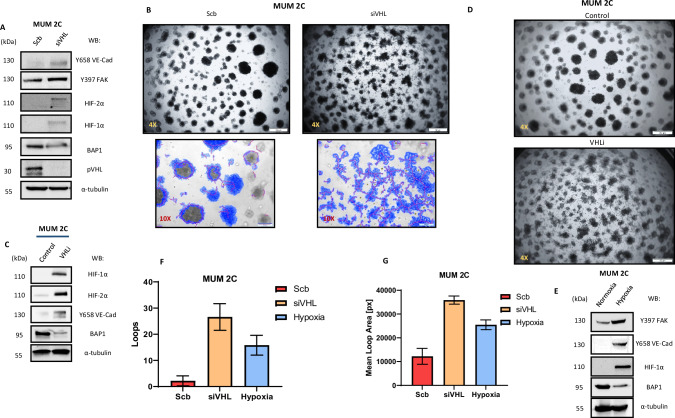
Depletion of VHL/PHD2 enhances p-FAK and VE-Cadherin Y658 phosphorylation, promoting vasculogenic mimicry. **A** Western blot experiments in MUM 2C after siVHL. **B**, **C** In vitro angiogenesis assay with Matrigel in MUM 2B showed the effect of siVHL conditions or **D** VHL inhibitor (50 µM during 24 h). **E** Western blot experiments in MUM 2C after VHL inhibitor (50 µM during 24 h). **F**, **G** Images were acquired using an Olympus CKX41 microscope (10X or 4X lens) (bars 50 μm) and the formation of tube-like structures was then quantified by Wimasis program.

Following the fact that the MUM 2B cell line has a monosomy of chromosome 3 and aware that VHL is located on this chromosome, we decided to silence VHL and examine the previously described VM markers. We discovered (Fig. 3A) that silencing VHL increases the phosphorylation of FAK at Y397, which consequently increases the phosphorylation of VE-cadherin at Y658. This shift moves the entire pool of VE-cadherin to a phosphorylated state. This effect may also be partially due to a decrease in p120-catenin (which, as reported, binds at the Y658 residue of VE-Cad [21, 53]. Additionally, we observed that NRP-1 [49] (the co-receptor of VEGFR2) did not increase, but TIE-1 [39], a receptor closely associated with VM, did. We confirmed the silencing of VHL with classical hypoxia markers such as HIF-1α and HIF-2α. This experiment also showed a decrease in total β-catenin, with a corresponding increase in non-phospho β-catenin (active β-catenin). Therefore, we also decided to silence PHD2 (Fig. 3B), obtaining similar results with an increase in FAK Y397 and VE-cadherin Y658 phosphorylation. Both silencing experiments, when performed in 3D Matrigel assays, showed that depletion of either VHL or PHD2 visibly increases loop area by MUM 2B cells in 3D Matrigel cultures, consequently decreasing the number of loops, as represented in Fig. 3C, Supplementary Fig. S4 and quantified by Wimasis program (Fig. 3F, G) and increased the mRNA levels of CCND1 intimately correlated with VM [20, 22] (Supplementary Fig. S5D), but not in siPHD1 experiments shown in the Supplementary Fig. S2C, F, J. We decided to silence VHL (Fig. 4A, B) in the MUM 2C cell line, which neither forms VM nor expresses VM markers. Surprisingly, after VHL silencing and VHL inhibitor treatment [54], these cells changed their morphology in matrigel, attempting to form tubules formation (quantified by Wimasis program, Fig. 4F, G) and increase the mRNA level of VE-Cad (Supplementary Fig. S2H, I). This was accompanied by an increase in Y658 phosphorylation of VE-cadherin, Y397 phosphorylation of FAK, and a decrease in BAP1 expression, resembling the protein profile of MUM 2B cells, as shown in Fig. 4A–D. Observing this behavior in both MUM 2C and MUM 2B cells. We decided to test the impact of general hypoxia on VM markers and cell morphology in Matrigel. We observed that under hypoxia, both cell lines increased the expression of Y658 VE-Cad as well as Y397 FAK and decreased the expression of BAP1 in MUM 2C cells, exhibiting a protein profile similar to that of MUM 2B cells, consequently enhancing the formation of VM (Figs. 3D, E and 4E) (Supplementary Fig. S5A, B).

VM was first described in uveal melanoma. Despite the rarity of this tumor type, several seminal articles have highlighted that approximately 80–90% of uveal melanomas possess a GNAQ or GNA11 mutations but it does not have a clear relationship with overall survival in patients with UM (Supplementary Fig S2K). These mutations lead to constitutively active Gαq proteins, making them driver oncogenes. This aberration disrupts downstream kinase mechanisms, particularly affecting FAK activity at Y397. The altered FAK activity impacts the localization and phosphorylation of YAP, enhancing YAP’s transcriptional activity in the nucleus [8, 55]. As seen in the previously cited articles, clozapine N-oxide (CNO) is a synthetic ligand that can activate the established synthetic Gαq-coupled GPCR (Gαq-DREADD). We decided to test this activator of FAK phosphorylation in the MUM 2B cell line (Supplementary Fig. S2A). The results showed that CNO did not activate FAK in these cell lines, even when combined with siVHL. This lack of activation may be partly due to the MUM 2B cell line already exhibiting high basal expression of FAK as a result of chromosome 8 gain, which is also observed in patients with Ch3 loss Ch8 gain (as shown in Fig. 2A). Additionally, CNO did not activate VE-Cad phosphorylation in MUM 2B and reduced de VM formation in MUM 2B (Supplementary Fig. S2B, E). Moreover, UM cell lines 92.1 and OMM.1 do not form VM (Supplementary Fig. S3F). We hypothesize that primary mutations in GNAQ or GNA11 contribute mechanistically to the activation of FAK but do not directly influence the initial expression of FAK in UM patients or the formation of VM. This observation supports the prioritization of classifying UM patients based on chromosomal aberrations, such as Ch3p loss/Ch8q gain, or BAP1 mutations, as these factors are associated with the poorest overall survival outcomes (Figs. 1A, 2B). Finally, we decided to observe and investigate the potential effect of siHIF-1α and siHIF-2α and their impact on VM formation and VM markers. We observed (Supplementary Fig. S2D, G, J) that only siHIF-2α decreases VM formation in matrigel experiments (quantified by Wimasis program, Supplementary Fig. S2F), reducing the expression of VE-Cad and NRP-1 at both the protein and mRNA levels (Supplementary Fig. S2G). Additionally, we conducted IP-CE and NE experiments on β-catenin in MUM 2B cells, demonstrating that β-catenin enhances the nuclear binding of VE-Cadherin following siVHL treatment, Supplementary Fig. S5C.

It has been observed that VM formation may be associated with a worse prognosis in other tumors. VM was first described in uveal melanoma, but later this phenomenon has been seen in various tumors, one of them being Ewing sarcoma [56] and related with ENG expression in Ewing sarcoma tumor by hypoxia [57, 58]. Therefore, knowing the possible implication of monosomy 3 and the gain of chromosome 8 in VM formation through HIFs stability, we decided to explore these chromosomal aberrations in Ewing sarcoma cell lines. We found through cBioPortal (a database of global cell lines) that various Ewing sarcoma cell lines have these parameters. Specifically, we found that the SKNMC cell line has a deep deletion of VHL and BAP1 located on chromosome 3p and an amplification of PTK2 on chromosome 8q. We then conducted VM expression experiments in these cell lines compared to other Ewing sarcoma cell lines. We observed that the SKNMC cell line expresses the Y397 of FAK and the Y658 of VE-cadherin and can form VM (Supplementary Fig. S3A–C). We then depleted PHD2 to explore the potential consequences of increased HIF-2α on VM formation. We found that this silencing does not change VM formation or the expression of VM markers (Supplementary Fig. S3D, E). Surprisingly, we found that the TC-71 Ewing sarcoma cell line has high basal expression of HIF-2α and forms more VM, partly due to the basal level of ENG in these cells (Supplementary Fig. S3D, E).

### VE-Cadherin/ENG forms a complex in a FAK-dependent manner

**Fig. 5:**
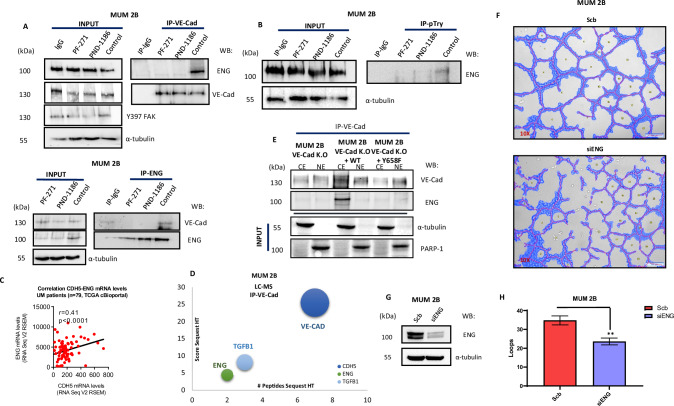
VE-Cadherin/ENG forms a complex in a FAK-dependent manner. **A** Immunoprecipitation after FAKi (PND-1186 and PF-271 (1 µM during 24 h) of VE-Cadherin, ENG or **B** pTyr B in MUM 2B cells, input was used to protein expression controls. **C** Evaluation of the correlation between ENG mRNA levels in a cohort of uveal melanoma (*n*  =  79, TCGA Firehose legacy) using cBioPortal. Correlation analysis was done using the Spearman test. **D** Immunoprecipitation of VE-cadherin experiments shows union with ENG/TGFB1 in MUM 2B cells, representative union catenin to VE-cadherin graph of LC-MS proteomics IP-VE-cadherin, score sequest HT in the X axis and #peptides sequest HT in Y axis. **E** Immunoprecipitation after cytosol-nucleus fractionation of VE-Cadherin in MUM 2B cells, VE-Cad K.O, VE-Cad K.O WT VE-Cad, VE-Cad K.O Y658F construct (1 µgr during 48 h), input was used to protein expression controls, α-tubulin cytosol control or PARP-1 nucleus control. **F**–**H** In vitro angiogenesis assay with Matrigel in MUM 2B showed the effect of siENG, images were acquired using an Olympus CKX41 microscope (10X lens) (bars 50 μm) and the formation of tube-like structures was quantified by Wimasis program. siRNA of ENG was confirmed by western blot experiments.

As described and published by our group in recent years [20, 22], the highly aggressive MUM 2B cell lines, which are capable of VM formation, express endothelial markers. Exploring LC-MS experiment data [22] through the immunoprecipitation of VE-Cad from different VE-Cad localizations, we found that VE-Cad binds to endoglin (ENG) as well as the TGFB1 receptor (Fig. 5D). Following this finding, we conducted immunoprecipitation experiments after inhibiting FAK (using PF-271 and PND-1186), which we have demonstrated is highly related to the binding of various catenins to VE-Cad. We observed that VE-Cad indeed binds to ENG in the MUM 2B cell line and that FAK inhibitors abolish this binding (Fig. 5A, B). Endoglin also binds to VE-Cadherin only in the MUM 2B KO VE-Cad cell line rescued with WT VE-Cad, and does not bind in the MUM 2B KO Y658F cell line (Fig. 5E). This suggests that the binding of VE-Cad to ENG is partially dependent on the phosphorylation of VE-Cad by FAK, corroborating the previously mentioned experiments. In the next step, we explored the direct consequence of ENG on VM formation in MUM 2B. To do this, we silenced ENG and conducted VM experiments in Matrigel. As shown in Fig. 5F, G after ENG silencing, VM formation was significantly reduced and quantified by the Wimasis program represented in Fig. 5H.

### Belzutifan in combination with FAKi: promising candidates for the treatment of metastatic uveal melanoma patients

It has been reported that Belzutifan is a promising drug for patients with ccRCC harboring VHL mutations [26]. However, the relationship between chromosome 3 loss and the subsequent loss of VHL in uveal melanoma patients has not yet been explored. Additionally, knowing that these patients with chromosome 3 loss have chromosome 8 gain, and that PTK2 (FAK) is located on chromosome 8, we logically decided to conduct experiments with HIF-2α inhibitors combined with FAK inhibitors. This dual action targets genes affected by initial chromosomal aberrations. Preclinical studies have shown that the activation and elevated expression of focal adhesion kinase (FAK) is associated with tumor progression, invasion, and drug resistance in solid tumors, prompting the development of FAK inhibitors. Four FAK inhibitors (GSK2256098, PF-00562271, VS-6063, and BI853520) have proven effective in preclinical studies and have progressed to phase I and II clinical trials. While FAK inhibitors have demonstrated efficacy in delaying progression-free survival and maintaining stable disease in some advanced solid tumors, they have not produced objective clinical responses. Prognostic biomarker-driven studies are necessary to identify patients most likely to benefit from FAK inhibitor treatment. Current clinical trials explore treatment strategies combining FAK inhibitors with chemotherapy, targeted therapy, or checkpoint inhibitors to enhance efficacy [59].

**Fig. 6:**
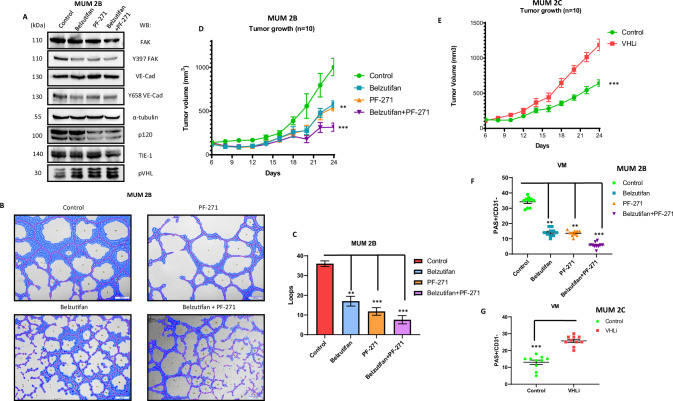
Belzutifan in combination with FAKi: promising candidates for the treatment of metastatic uveal melanoma patients. **A** Western blot experiments in MUM 2B after Belzutifan (1 µM during 24 h) and PF-271 (1 µM during 24 h), **B** In vitro angiogenesis assay with Matrigel in MUM 2B showed the effect of PF-271 (1 µM during 24 h) or Belzutifan (1 µM during 24 h), images were acquired using an Olympus CKX41 microscope (10× lens) (bars 50 μm) and the formation of tube-like structures was then quantified by **C** Wimasis program. **D**, **E** Tumor growth progression graphic representation on different days, the annotations of the tumor were every two days, being the day 6 post-injection of MUM 2B or MUM 2C cells the beginning of different treatments. **F**, **G** Graphic representing of CD31-/PAS+ regions in MUM 2B and MUM 2C xenografts experiments. Asterisks denote significance in an unpaired t-test (*p*  <  0.05, *p*  <  0.01, *p*  <  0.001), and error bars denote SD.

We confirmed that both inhibitors reduce the expression of FAK Y397 and, consequently, Y658 of VE-Cad, suggesting that the combination of these inhibitors is promising for inhibiting VM formation (Fig. 6A–C, Supplementary Fig. S7A). We also conducted VM experiments following PHD2 and VHL silencing in the MUM 2B cell line in combination with Belzutifan plus FAKi (Supplementary Fig. S6A, B). With this aim, we set up a xenograft approach with MUM 2B cells in which we combined the FDA-approved to clinic-used treatment Belzutifan plus a potential specific inhibitor of Y397 phosphorylation, PF-271 (Fig. 6A–C), to limit VM, according to us in vitro results. Tumor growth was evaluated 24 days after the inoculation of MUM2B in nude mice (*N*  =  10 per group), the combination of Belzutifan plus PF-271 reduced global tumor progression, as well as the final tumor growth (Fig. 6D). We performed a xenograft approach using MUM 2C cells treated with a VHL inhibitor. Tumor growth was evaluated 24 days post-inoculation in nude mice (*N* = 10 per group), revealing a significant increase in overall tumor progression (Fig. 6E). Notably, the reduction in CD31−/PAS+ regions (used as a surrogate for VM areas) in tumors treated with the Belzutifan and PF-271 combination is shown in MUM 2B (Fig. 6F, Supplementary Figs. S7B,  S8), whereas in MUM 2C, treatment with the VHLi increased CD31−/PAS+ regions, resembling levels observed in MUM 2B (Fig. 6G, Supplementary Figs. S7B,  S8) (Supplementary Table S1).

## Discussion

Germline loss-of-function mutations in the VHL tumor suppressor gene cause von Hippel–Lindau disease, increasing the risk of hemangioblastomas, ccRCCs, and paragangliomas. The VHL protein targets the hypoxia-inducible factor (HIF) for degradation when oxygen is present [60, 61]. Deregulation of HIFs, especially HIF-2α, promotes the growth of VHL-defective ccRCCs. VEGF inhibitors are standard treatments for ccRCC. Recently, an allosteric HIF-2α inhibitor was approved for VHL-associated ccRCCs and is in phase III testing for sporadic ccRCCs. Furthermore, the formation of VM has recently been correlated with ccRCC [62, 63]. However, the implications of VHL in uveal melanoma patients have yet to be explored. In the early studies of VM, this phenomenon was curiously described in cell lines of uveal melanoma origin. In the present research, we have demonstrated that metastatic UM cell lines (MUM 2B and MUM 2C) exhibit significant chromosomal changes, with VHL playing a fundamental role in VM formation. These highly aggressive cells can maintain basal hypoxia due to the loss of VHL functionality over cellular HIFs. This leads to the aberrant activation of genes involved in classical angiogenesis and the VM phenomenon through HIF-2α and VE-Cad. Importantly, these cells or patients have amplification of chromosome 8q and consequently PTK2 (FAK), which is highly correlated with post-translational changes in VE-Cad, as well as the YAP/TAZ pathway in UM [8]. Additionally, nuclear localization of FAK increases certain cytokines like CCL5 and CXCL10 levels, suggesting a potential immune response and possible immunotherapeutic treatments in uveal melanoma patients as demonstrated in squamous cell carcinoma [36]. Knowing this and as we have presented, uveal melanoma patients with monosomy of chromosome 3p (implicating VHL and BAP1) and gain of chromosome 8q (PTK2) have a worse prognosis. Therefore, we believe these patients should be individually prioritized for study and supported by new patient monitoring technologies. Considering all this, we propose that these patients could receive prophylactic treatment with Belzutifan plus FAK inhibitors. Additionally, this treatment could be considered for patients who present with characteristics of chromosome 3p loss and chromosome 8q gain with initial metastasis at diagnosis.

Considering the recent approval of treatment for ccRCCs and the potential application to other tumors related to VHL syndrome, we can hypothesize that the proposed treatment with Belzutifan plus FAK inhibitors for patients with chromosome 3p loss and chromosome 8q gain might be the best personalized approach for these patients, who have the worst prognosis, overall survival, and disease-free survival. Therefore, we believe this drug combination could be tested in future clinical trials. This article could open the door to treating a rare and often overlooked disease, metastatic uveal melanoma. The question remains open whether the deep deletion of VHL or BAP1, or their combination, along with chromosome 8q amplification and PTK2 (FAK) gain of function, might be implicated in the pathogenesis of other tumors. As we have hinted in Ewing sarcoma cell lines, we lack sufficient patient data to draw definitive conclusions. However, the results presented here could pave the way for future original articles on this topic and emphasize the importance of functional studies on the deep deletion of VHL and BAP1 combined with FAK amplification.

## Conclusions

**Fig. 7:**
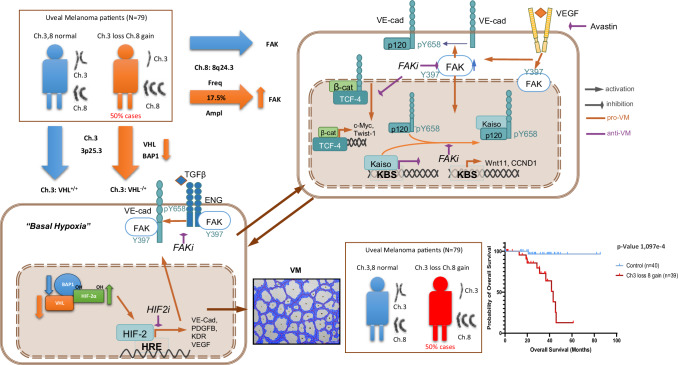
Graphical figure summarizing the origin of VM in UM patients. The loss of chromosome 3 combined with the gain of chromosome 8 culminates in the ability to form VM due to sustained basal hypoxia from VHL loss of function on HIF-2α. Further details are explained in the “Discussion” and “Conclusion “section.

Figure 7 summarizes the pivotal conclusions regarding the origin of VM in UM patients. The combined loss of chromosome 3p and gain of chromosome 8q leads to VM formation, driven by sustained basal hypoxia due to VHL/BAP1 loss of function on HIF-2α. This hypoxic state subsequently by Ch8 gain, activates FAK, which in turn phosphorylates VE-cadherin, promoting TCF-4/Kaiso-mediated gene expression. Patients with these chromosomal abnormalities have the poorest prognosis. Therefore, targeting the basal hypoxia driven by VHL loss and inhibiting FAK activation represents the most effective personalized therapeutic strategy for UM patients.

## Materials and methods

### Reagents and antibodies

The following reagents were used: Belzutifan (PT2977, Selleckchem) 1 μM during 24 h in vitro assays and 25 mg/Kg oral gavage two days per week in tumor xenograft assay. PF-562271 (Selleckchem) 1 μM during 24 h in vitro assays and 30 mg/Kg oral gavage every two days in tumor xenograft assay, VHL inhibitor (VH298, Abcam, ab230370), 50 μM during 24 h in vitro assays and 25 mg/Kg intraperitoneal two days per week in tumor xenograft assay. PND-1186 (Selleckchem) 1 μM during 24 h in vitro assays, CNO 1 μM during 24 h. Corning Matrigel Basement Membrane Matrix for in vitro angiogenesis experiments. Antibodies used were: Y658 VE-Cad rabbit (1:1000 WB, 1:100 IF, Thermofisher), VE-Cad (1:1000 WB, 2 µg IP, clone D87F8, cell signaling), β-catenin mouse (1:1000 WB, 2 µg IP, sc-7963), non-phopho β-catenin mouse (1:1000 WB, 1:100 IF, Clone D13A1, cell signaling), phopho Y397 FAK rabbit (1:1000 WB, Clone 44-624 G, Thermofisher), FAK rabbit (1:1000 WB, Clone C-20, sc-558), HIF-2α mouse (1:500 WB, 5 µg ChiP, 2 µg IP, Clone 190b, sc-13596), p120 mouse (1:1000 WB, Clone pY228, BD Biosciences), PHD2 mouse (1:500 WB, Clone H-8, sc-271835), VHL rabbit (1:1000 WB, 2 µg IP, Clone E3X9K, cell signaling), HIF-1α rabbit (1:1000 WB, Clone D1S7W, cell signaling), BAP1 rabbit (1:1000 WB, 2 µg IP, Clone D7W7O, cell signaling), CD105 rabbit (1:1000 WB, 2 µg IP, Clone EPR10145-10, Abcam), NRP-1 mouse (1:500 WB, Clone A-12, sc-5307), TIE-1 rabbit (1:1000 WB, Clone D2K2T, cell signaling), Slug rabbit (1:1000 WB, Clone C19G7, cell signaling), α-tubulin mouse (1:10000 WB, clone B-5-1-2, Sigma-Aldrich), lamin B1 rabbit (1:1000 WB, Abcam) and PARP-1 mouse (1:1000 WB, Calbiochem), Twist-1 rabbit (1:1000 WB, Clone E7E2G, cell signaling), c-Myc mouse (1:1000 WB, Clone 9E10, sc-40) and H2AK119ub1 rabbit (1:1000 WB, Clone D27C4, cell signaling).

### Cell lines and construction of GFP-tagged VEC

Human uveal melanoma cells MUM 2B, MUM 2C, 92.1 and OMM-1 were grown in RPMI medium supplemented with 10% fetal bovine serum, 2 mM of L-glutamine, and 1% penicillin/streptomycin (PAA laboratories). All cells were cultured at 37 °C and 5% CO2 in incubator cells. Ewing Sarcoma cells EW7, TC-71 and SKNMC were grown in RPMI medium supplemented with 10% fetal bovine serum, 2 mM of L-glutamine, and 1% penicillin/streptomycin (PAA laboratories). Ewing Sarcoma cells A673 were grown in DMEM medium supplemented with 10% fetal bovine serum, 2 mM of L-glutamine, and 1% penicillin/streptomycin (PAA laboratories). cDNA of human VEC fused in-frame with GFP at the COOH-terminus (VEC-GFP), VEC Y658F was a kind of gift from Dr. Masahiro Murakami. These constructs were subcloned into pcDNA3.1 (Invitrogen), and previously validated [20].

### In vitro angiogenesis assay

The effect of PF-271, Belzutifan after 24 h or siVHL, siPHD2, siPHD1, siHIF-1α, siHIF-2α and siENG after 48 h on the formation of tube-like structures in Matrigel (BD Biosciences) was resolved according to the manufacturer’s instructions and previously describe [21]. After 48 h, respectively, of incubation, images were acquired using an Olympus CKX41 microscope (4X and 10X lenses). The formation of tube-like organization was quantified by Wimasis program. Each treatment was realized in triplicate, and the experiments independently repeated at least three times.

### Quantitative RT-PCR

Total RNA was isolated by RNeasy Mini Kit (Qiagen) according to the manufacturer’s recommendations and previously described [20]. Each reaction was performed in triplicate using CFX96 Real-time PCR detection systems. Primer sequences for the targets and the annealing temperature (60 °C): 36B4: Forward 5′-CAGATTGGCTACCCAACTGTT-3′, Reverse 5′-GGCCAGGACTCGTTTGTACC-3, VE-Cad: Forward 5′- AAAGGCTGCTGGAAAATG -3′, Reverse 5′- AACTTCCCCTTCTTCACCC -3′; CCND1: Forward 5′-CCGTCCATGCGGAAGATC-3′, Reverse 5′-GAAGACCTCCTCCTCGCACT-3′.

### Gene editing

MUM2B knockout (ko) cells for the VE-Cad gene were composed using the CRISPR-Cas9 technology. Five different sgRNAs were designed using the Zhang Lab Optimized CRISPR design tool and cloned into the pL-CRISPR.EFS.GFP which purchased from the Addgene public repository (#57818) and previously described [20].

### Transfection of small interfering siRNA

Gene silencing experiments were conducted using siRNA (small interfering RNA) transfection. JetPRIME reagents and buffer from Polyplus were used, following the protocol for transfection in 6-well plates and 0.25 × 106 cells per wells during 48 h. The siRNAs used were ON-TARGETplus siRNAs, SMARTPool 5 nmol, from Dharmacon Reagents, 50 nM working concentration. siRNAs target PHD2 (Human EGLN1, #L-004276-00-0005), PHD1 (Human EGLN2, #L-004277-00-0005), VHL (Human VHL, #L-003936-00-0005), HIF-2α (Human EPAS1, #L-004814-00-0005), HIF-1α (Human HIF1, #L-004018-00-0005), ENG (Human ENG, #L-011026-00-0005) and a control (Non-Targeting Control, #D-001810-01-05). After completing the silencing protocol, total protein extraction from the cultures was performed, or the cells were seeded in Matrigel for 3D growth assays.

### Chromatin Immunoprecipitation (ChIP)

Cells were grown to approximately 80%-90% (18×10^6^ cells per IP) confluence with scb, siPHD2 into 24 h. The culture medium was aspirated and cells were washed twice with cold PBS. ChIP-HIF-2α (5ugr) and IgG (2ugr) was performed following SimpleChIP Enzymatic Chromatin IP Kit (Magnetics beads) (Cell signaling). qPCR promoter specific primers used: VE-Cad promoter, forward 5’-GACCACAAGGCCTGGGCA-3’, Reverse 5’- GCAACGGGTCATGCTAGGAT-3’.

### Immunobloting, immunoprecipitation, subfractionation cytosol-nucleus

Simple coimmunoprecipitation, cells were lysed in lysis buffer (50 mM Tris/HCl ph 8, 120 mM NaCl, 0,1% NP-40, 1 mM EDTA, 10 mM NaF, 1 mM Na3VO4 and supplemented with a protease inhibitor cocktail (1 tablet to 10 ml of lysis buffer, Roche) for 30 minutes at 4°C and previously described [20].

For subfractionation cytosol-nucleus, cells were lysed in lysis buffer (250 mM sucrose, 50 mM Tris-HCl ph 7,4, 5 mM MgCl2, 1 mM Na3VO4, 0,25% NP-40 and supplemented with a protease inhibitor cocktail (1 tablet to 10 ml of lysis buffer, Roche) for 10 minutes at 4 °C.

### LC-MS/ Gene enrichment analysis

Cells were resuspended in 60 μl after IP-CE, NE subfractionation, were precipitated with acetone and resuspended in 50 mM ammonium bicarbonate solution containing 8 M urea. After reduction and alkylation, samples were digested with trypsin and desalted with Clean-Up. Enriched samples were analyzed on nano-LC-MS in Easy-nLC 1000 (Proxeon) coupled to an ionic source with nanoelectrospray (ThermoScientific). Raw files were analyzed in Uniprot database using Sequest in Proteome Discoverer (ThermoScientific) (Proteomic department from UCO). Peptides identification was validated by Percolator using *q* value ≤ 0.01.

### Tumor Xenograft assay

Male Swiss Nude (SwN) mice were purchased from Charles River Laboratories and housed at IBIS animal facility according to institutional guidelines (Approved Ethical Committee). For xenograft generation, 1 × 10^6^ MUM 2B or MUM 2C cells in 100 µL PBS were subcutaneously injected in the flank of 7-weeks old mice. Animals (*n* = 10 per group) were monitored every two days after cell injection until final time point, when they were sacrificed and tumors were dissected for further analyses. Tumor volume was calculated as: in progress tumor volume = (π × length × width^2^)/6 and final tumor volume = (π × length × width × height)/6).

### Cytoscan 750 K microarray assay

DNA was extracted from MUM 2B and MUM 2C cells line using the DNeasy Blood and tissue kit (QIAGEN) according to the manufacturer’s instructions. The concentration and quality of DNA samples were evaluated by Nanodrop 2000 spectrophotometer (Thermo Scientific). DNA integrity was assessed by 1% agarose gel electrophoresis. The quality controls (QC) of Thermo Scientific CytoScan 750 K microarray required that DNA concentration should be no less than 50 ng/µL, OD260/280 is about 1.9, OD260/230 is about 2.0. The Thermo Scientific Cytoscan 750 K Microarray offers comprehensive genome-wide coverage, focusing on cytogenetically relevant regions with 550,000 markers for detecting copy number variations (CNVs) and 200,000 high-performing SNP probes with genotype accuracy over 99%. The protocol processes 8 to 24 samples simultaneously to obtain whole-genome CNV and SNP information. The workflow involves digesting gDNA, ligating with adaptors, amplifying via PCR, purifying PCR products, quantifying purified samples, fragmenting PCR products, labeling fragmented DNA, hybridizing with the microarray, washing and staining genechips, and scanning arrays. Data were analyzed using Chromosome Analysis Suite Version 2.0 with QC thresholds of SNPQC ≥ 15.0, MAPD ≤ 0.25, and Waviness SD ≤ 0.12, evaluating SNP array data quality. The microarray data were interpreted according to genome version GRCh37 (hg38). Only samples meeting QC criteria and identifying CNVs over 100 Kb with at least 10 aberrant probes were selected for further analysis. Identified CNVs were compared with the Database of Genomic Variants to exclude polymorphic variations. For UPD, an algorithm considering location and size of >5 Mb aberrations was used to exclude non-clonal regions. The total size of genomic alterations, including CNV and UPD, was calculated for each cell lines.

### Immunohistochemistry

Three-micrometer-thick tissue sections from paraffin blocks were dewaxed in xylene and rehydrated in a series of graded alcohols. Sections were immersed in 3% H2O2 aqueous solution for 30 min to exhaust endogenous peroxidase activity, then covered with blocking reagent (IHC Select HRP/DAB, thermofisher), to block nonspecific binding sites. Sections were incubated with primary antibodies for 24 h for CD31 rabbit (1:100 IHC, Clone D8V9E, cell signaling), HIF-2α (1:100 IHC) and p-FAK antibody (1:100 IHC). The sections were incubated with PAS solution for 5 min, and further stained with Schiff reagent for 15 min, followed by rinsing in distilled water and following the protocols by (Periodic Acid-Schiff (PAS) Staining System, thermofisher), and stained with haematoxylin solutions during 3 min The percentage of immunostained tumor cells was scored as follows: 0%, 0: negative; <19%, 1: weak, >20%, 2: middle and >50%, 3: positive quantified by pathologist (Supplementary Table S1). Representative images were acquired in a microscope (Olympus BX-61).

## Supplementary information

Supplementary material file

Table S1

WB original
