# Supplementary material for: Chromosomal 3p loss and 8q gain drive vasculogenic mimicry via HIF-2α and VE-cadherin activation in uveal melanoma
Source: Cell Death Differ. 2025 Feb 26;32(8):1473–83. doi: 10.1038/s41418-025-01469-9 (PMC12325912; doi:10.1038/s41418-025-01469-9)
Supplement: Supplementary file 1 — Supplementary material file [file 41418_2025_1469_MOESM1_ESM.pdf]

Fig S1

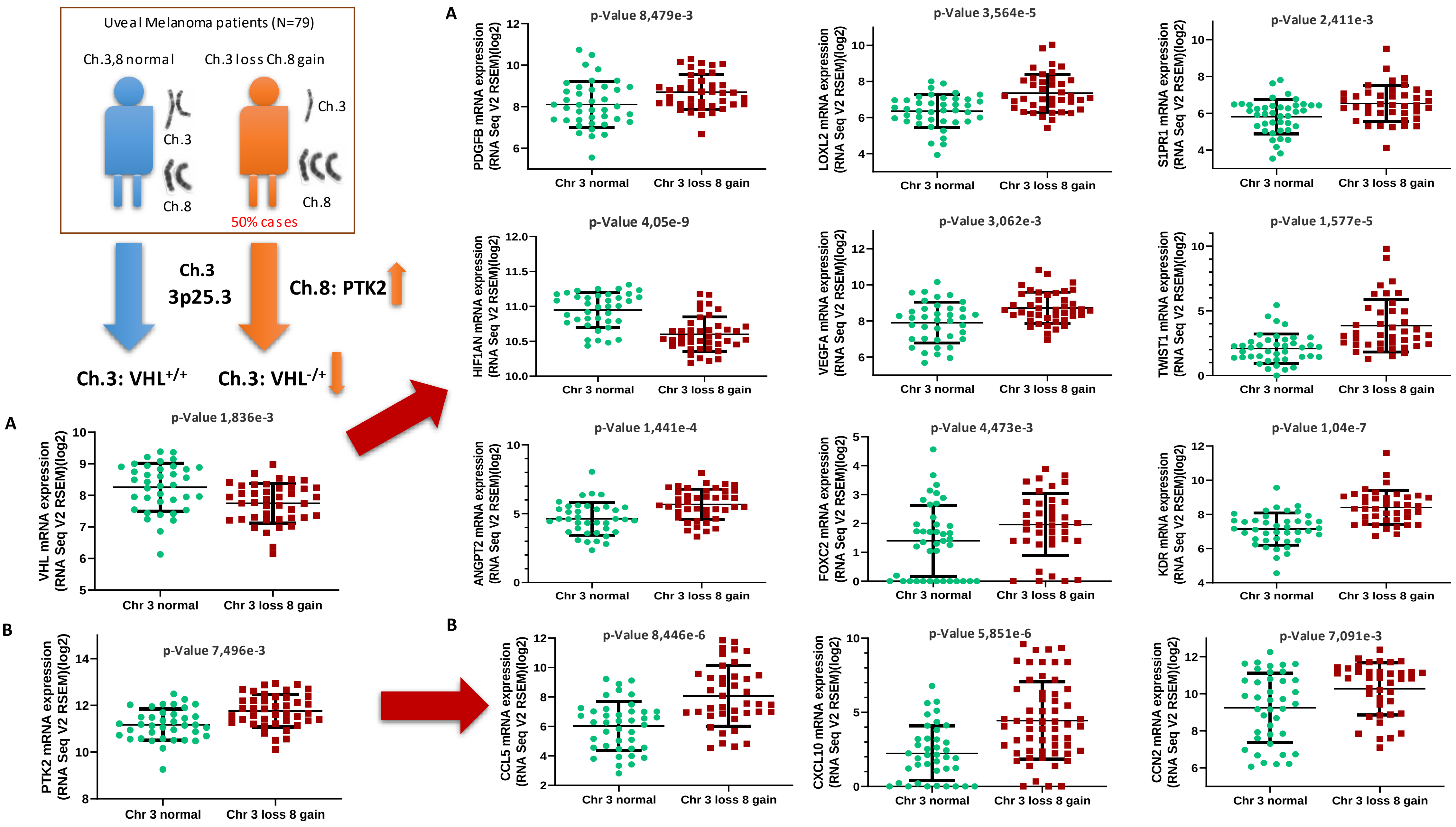
Uveal Melanoma patients (N=79)
Ch.3,8 normal
Ch.3 loss Ch.8 gain
Ch.3
Ch.8
Ch.3
Ch.8
50% cases
Ch.3 3p25.3
Ch.8: PTK2
Ch.3: VHL+/+
Ch.3: VHL-/+
A
p-Value 1,836e-3
VHL mRNA expression (RNA Seq V2 RSEM)(log2)
B
p-Value 7,496e-3
PTK2 mRNA expression (RNA Seq V2 RSEM)(log2)
A
p-Value 8,479e-3
PDGFB mRNA expression (RNA Seq V2 RSEM)(log2)
p-Value 3,564e-5
LOXL2 mRNA expression (RNA Seq V2 RSEM)(log2)
p-Value 2,411e-3
S1PR1 mRNA expression (RNA Seq V2 RSEM)(log2)
p-Value 4,05e-9
HIF1AN mRNA expression (RNA Seq V2 RSEM)(log2)
p-Value 3,062e-3
VEGFA mRNA expression (RNA Seq V2 RSEM)(log2)
p-Value 1,577e-5
TWIST1 mRNA expression (RNA Seq V2 RSEM)(log2)
p-Value 1,441e-4
ANGPT2 mRNA expression (RNA Seq V2 RSEM)(log2)
p-Value 4,473e-3
FOXC2 mRNA expression (RNA Seq V2 RSEM)(log2)
p-Value 1,04e-7
KDR mRNA expression (RNA Seq V2 RSEM)(log2)
B
p-Value 8,446e-6
CCL5 mRNA expression (RNA Seq V2 RSEM)(log2)
p-Value 5,851e-6
CXCL10 mRNA expression (RNA Seq V2 RSEM)(log2)
p-Value 7,091e-3
CCN2 mRNA expression (RNA Seq V2 RSEM)(log2)
Chr 3 normal
Chr 3 loss 8 gain

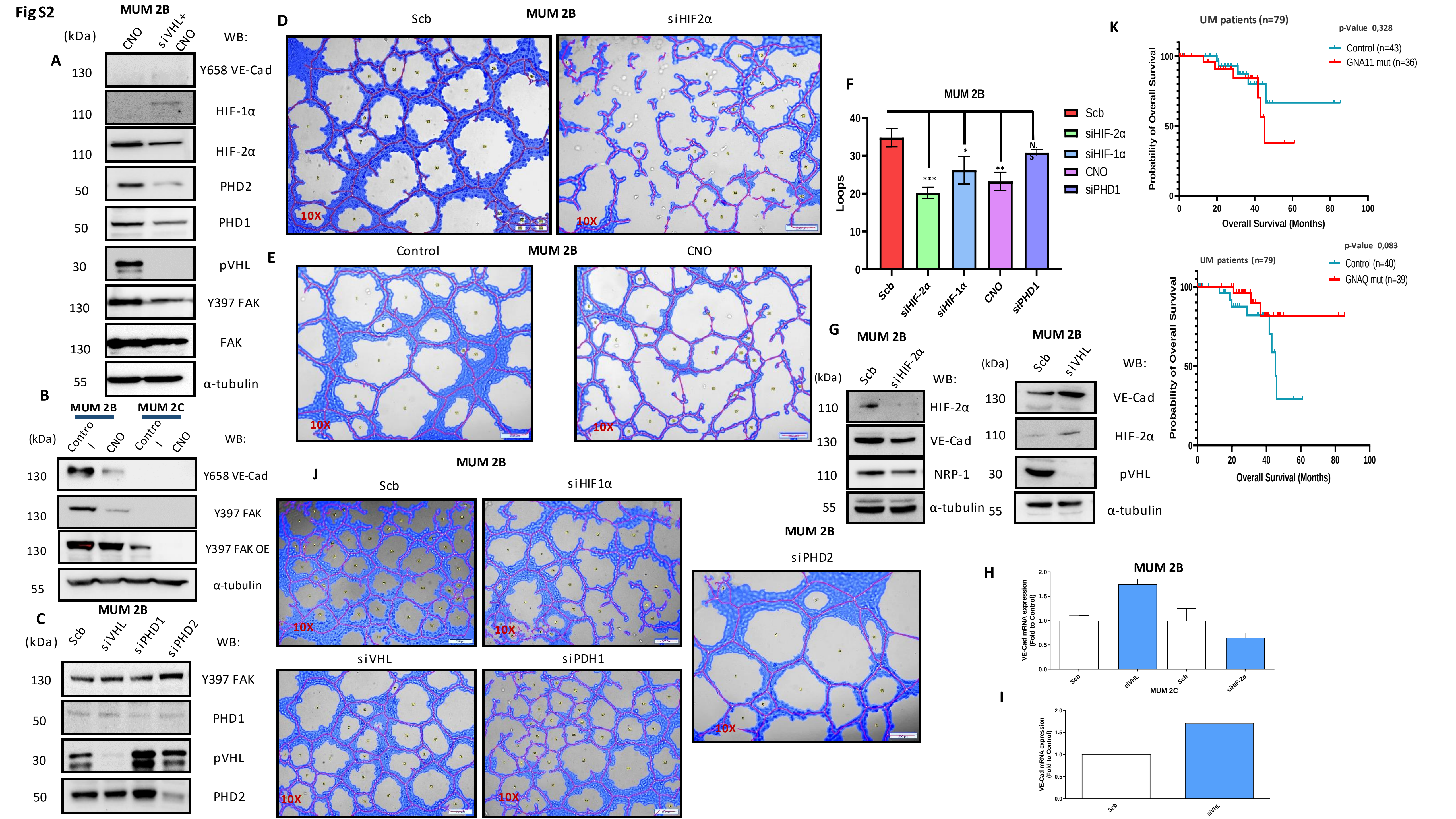
Fig S2
MUM 2B
(kDa)
CNO
siVHL+ CNO
WB:
A
130 Y658 VE-Cad
110 HIF-1α
110 HIF-2α
50 PHD2
50 PHD1
30 pVHL
130 Y397 FAK
130 FAK
55 α-tubulin
B
MUM 2B
MUM 2C
Control
CNO
130 Y658 VE-Cad
130 Y397 FAK
130 Y397 FAK OE
55 α-tubulin
C
MUM 2B
Scb
siVHL
siPHD1
siPHD2
130 Y397 FAK
50 PHD1
30 pVHL
50 PHD2
D
Scb
siHIF2α
10X
E
Control
CNO
F
Loops
Scb
siHIF-2α
siHIF-1α
CNO
siPHD1
***
*
**
N.S
G
110 HIF-2α
130 VE-Cad
110 NRP-1
55 α-tubulin
siVHL
130 VE-Cad
110 HIF-2α
30 pVHL
55 α-tubulin
H
VE-Cad mRNA expression (Fold to Control)
MUM 2C
I
J
siHIF1α
siVHL
siPDH1
siPHD2
K
UM patients (n=79)
p-Value 0,328
Control (n=43)
GNA11 mut (n=36)
Probability of Overall Survival
Overall Survival (Months)
p-Value 0,083
Control (n=40)
GNAQ mut (n=39)

Fig S3

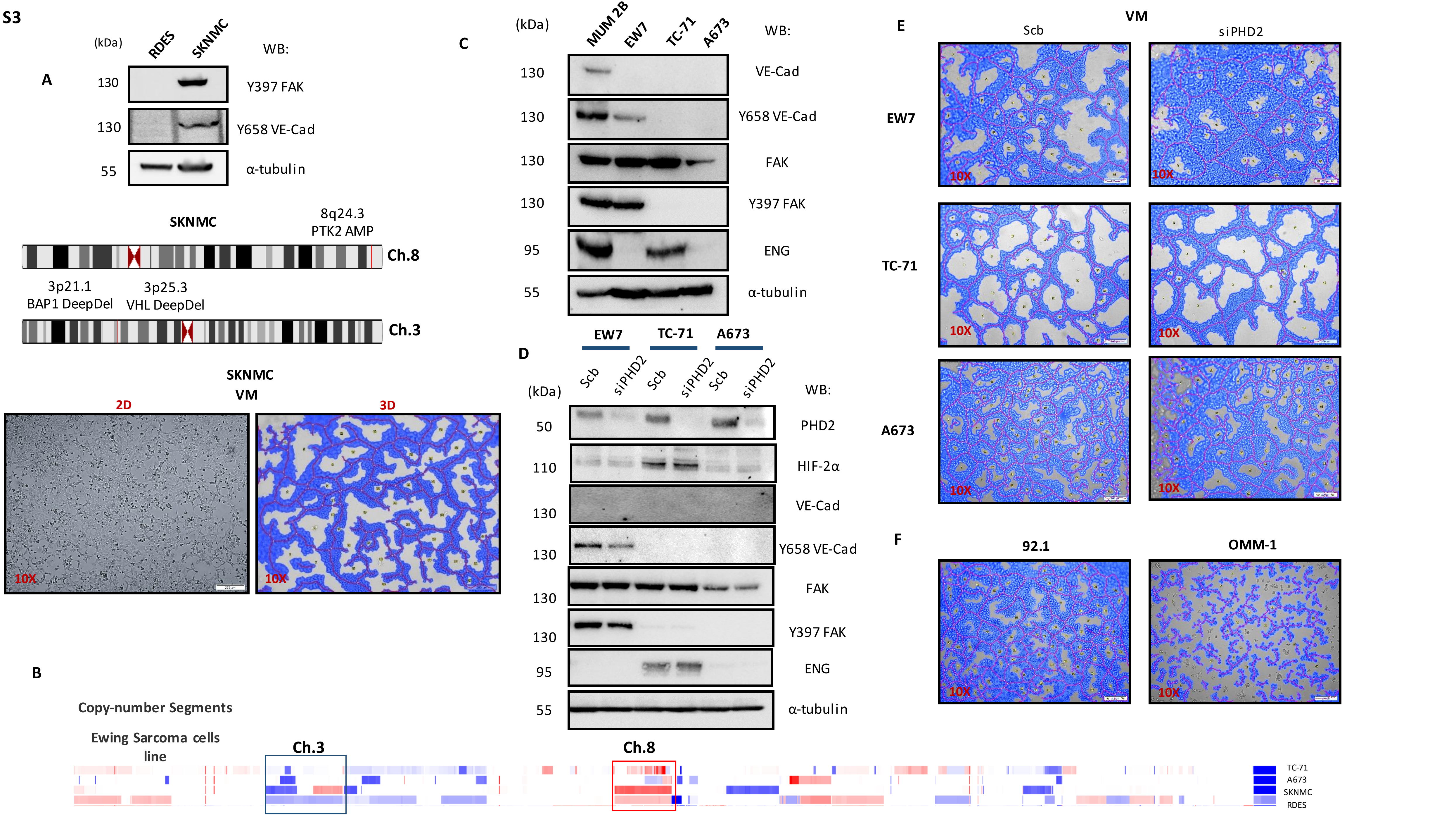
A
(kDa)
RDES
SKNMC
WB:
130
Y397 FAK
130
Y658 VE-Cad
55
α-tubulin
SKNMC
8q24.3
PTK2 AMP
Ch.8
3p21.1
BAP1 DeepDel
3p25.3
VHL DeepDel
Ch.3
SKNMC
VM
2D
3D
10X
10X
B
Copy-number Segments
Ewing Sarcoma cells line
Ch.3
Ch.8
TC-71
A673
SKNMC
RDES
C
(kDa)
MUM 2B
EW7
TC-71
A673
WB:
130
VE-Cad
130
Y658 VE-Cad
130
FAK
130
Y397 FAK
95
ENG
55
α-tubulin
D
EW7
TC-71
A673
Scb
siPHD2
Scb
siPHD2
Scb
siPHD2
(kDa)
WB:
50
PHD2
110
HIF-2α
130
VE-Cad
130
Y658 VE-Cad
130
FAK
130
Y397 FAK
95
ENG
55
α-tubulin
E
VM
Scb
siPHD2
EW7
TC-71
A673
10X
F
92.1
OMM-1
10X

Fig S4

MUM 2B

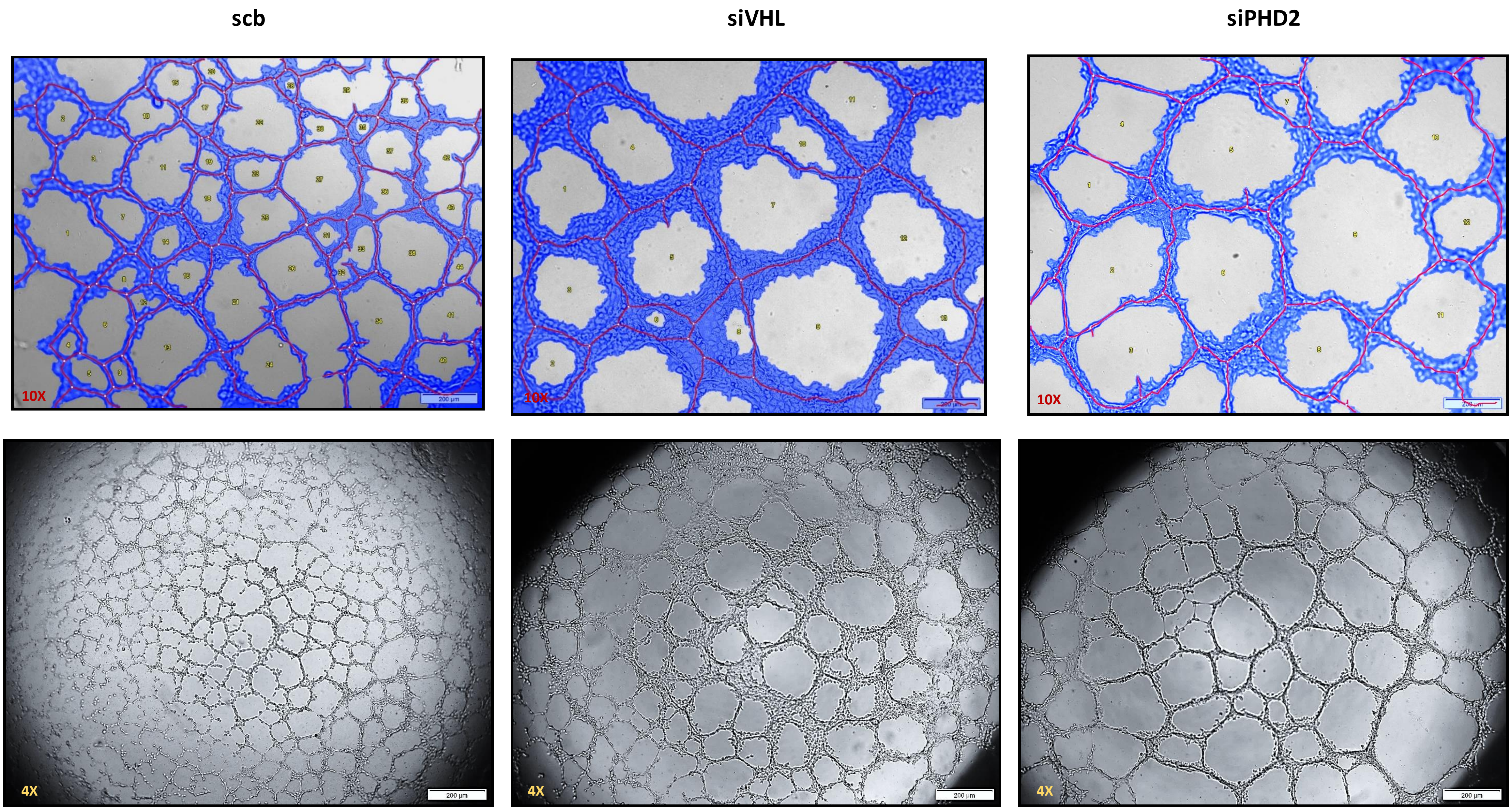

Fig S5

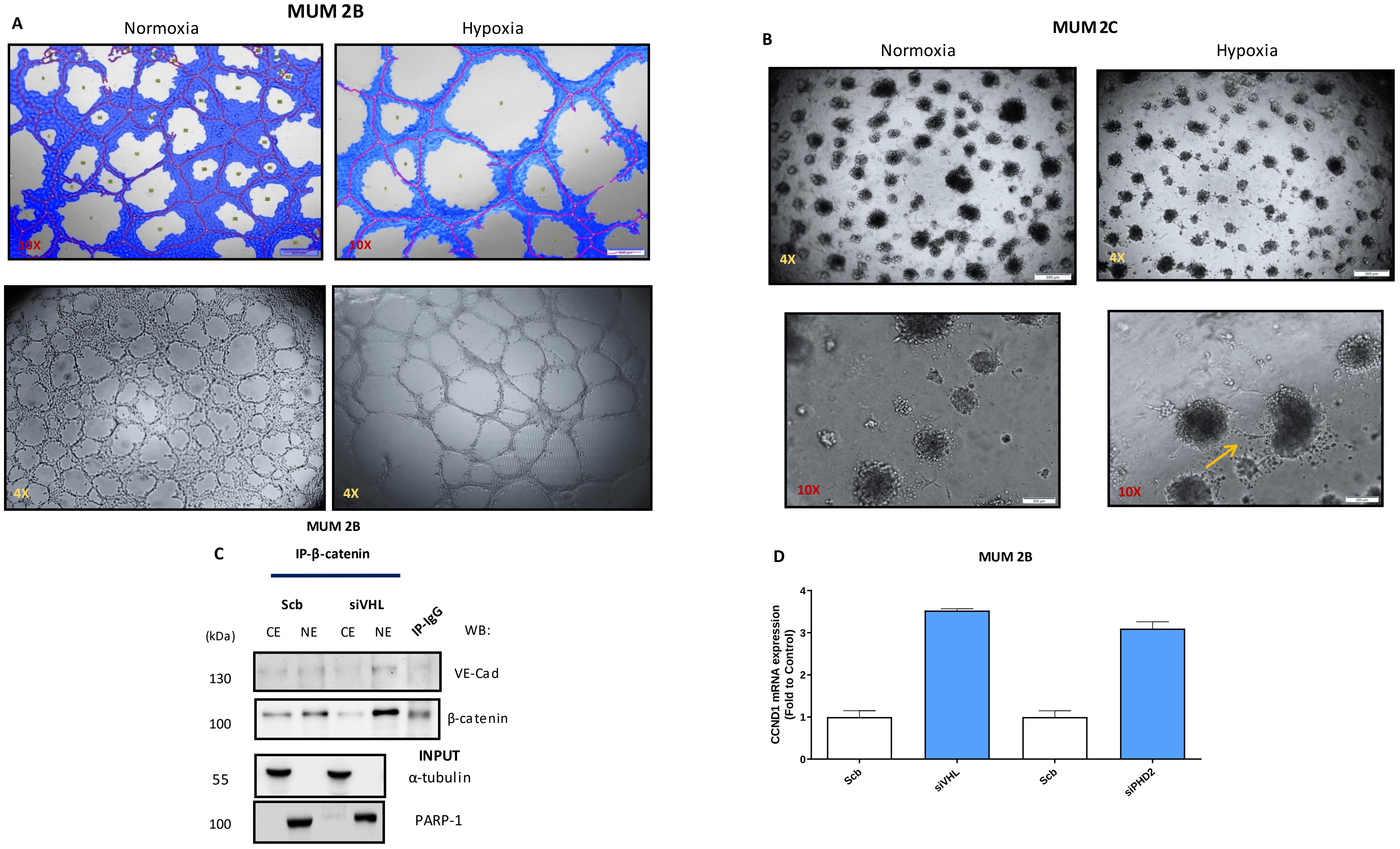
MUM 2B
A
Normoxia
Hypoxia
10X
10X
4X
4X
MUM 2C
B
Normoxia
Hypoxia
4X
4X
10X
10X
MUM 2B
C
IP-β-catenin
Scb
siVHL
IP-IgG
(kDa)
CE
NE
CE
NE
WB:
130
VE-Cad
100
β-catenin
INPUT
55
α-tubulin
100
PARP-1
D
MUM 2B
CCND1 mRNA expression
(Fold to Control)
4
3
2
1
0
Scb
siVHL
Scb
siPHD2

Fig S6

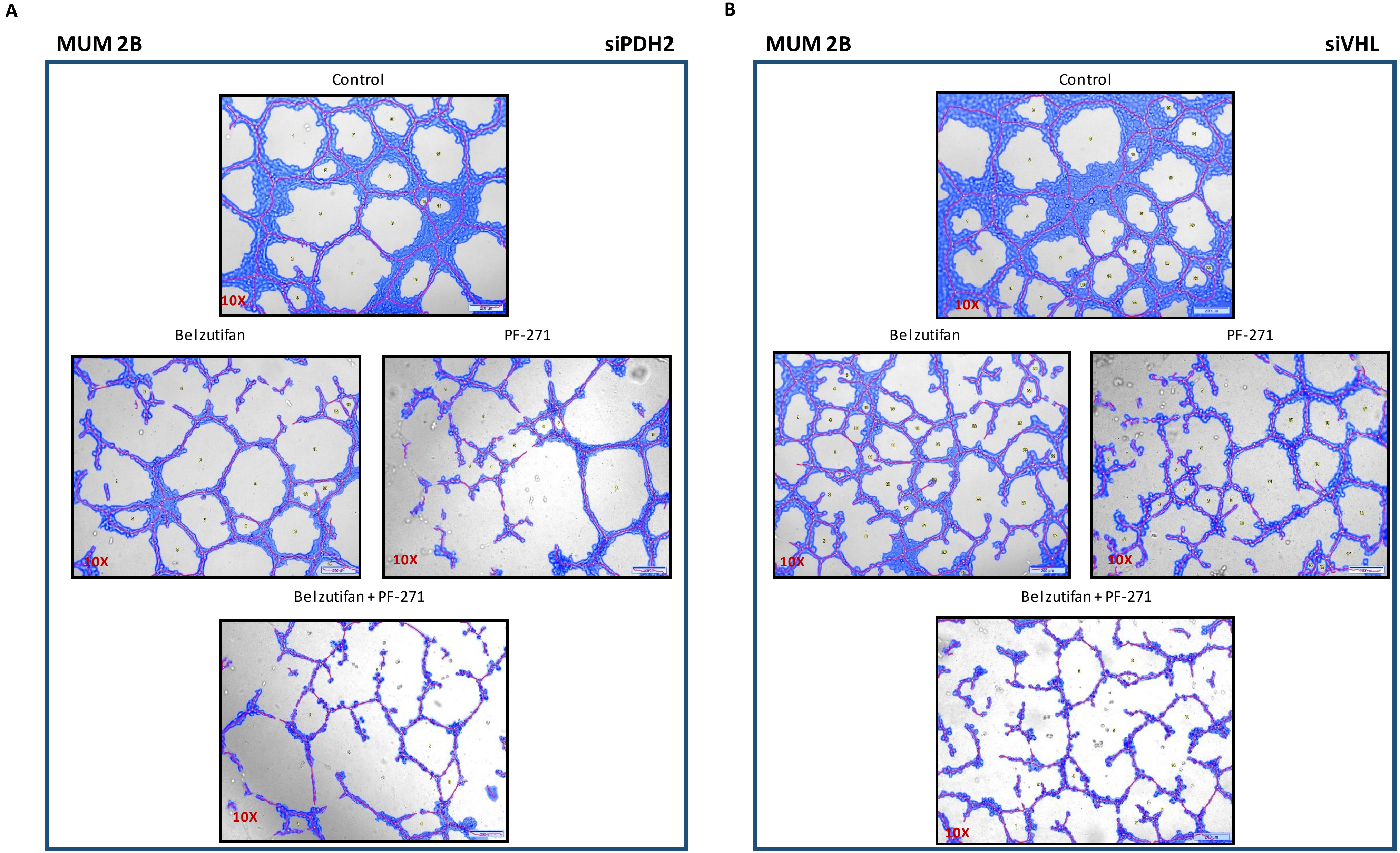
A
MUM 2B
siPDH2
Control
10X
Belzutifan
10X
PF-271
10X
Belzutifan + PF-271
10X
B
MUM 2B
siVHL
Control
10X
Belzutifan
10X
PF-271
10X
Belzutifan + PF-271
10X

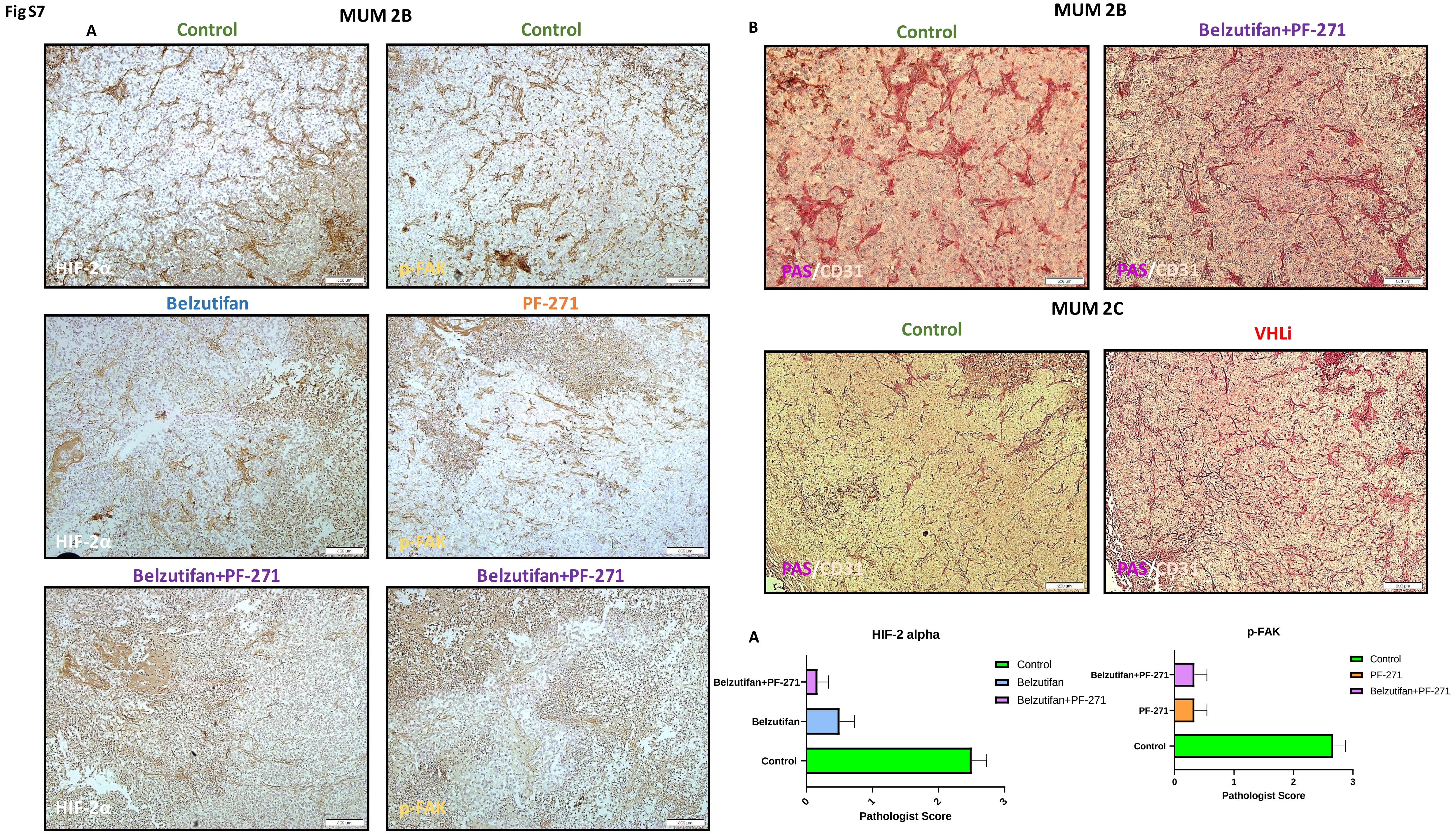
Fig S7
MUM 2B
A
Control
Control
HIF-2α
p-FAK
Belzutifan
PF-271
HIF-2α
p-FAK
Belzutifan+PF-271
Belzutifan+PF-271
HIF-2α
p-FAK
B
MUM 2B
Control
Belzutifan+PF-271
PAS/CD31
PAS/CD31
MUM 2C
Control
VHLi
PAS/CD31
PAS/CD31
A
HIF-2 alpha
Belzutifan+PF-271
Belzutifan
Control
Control
Belzutifan
Belzutifan+PF-271
0
1
2
3
Pathologist Score
p-FAK
Belzutifan+PF-271
PF-271
Control
Control
PF-271
Belzutifan+PF-271
0
1
2
3
Pathologist Score

Fig S8

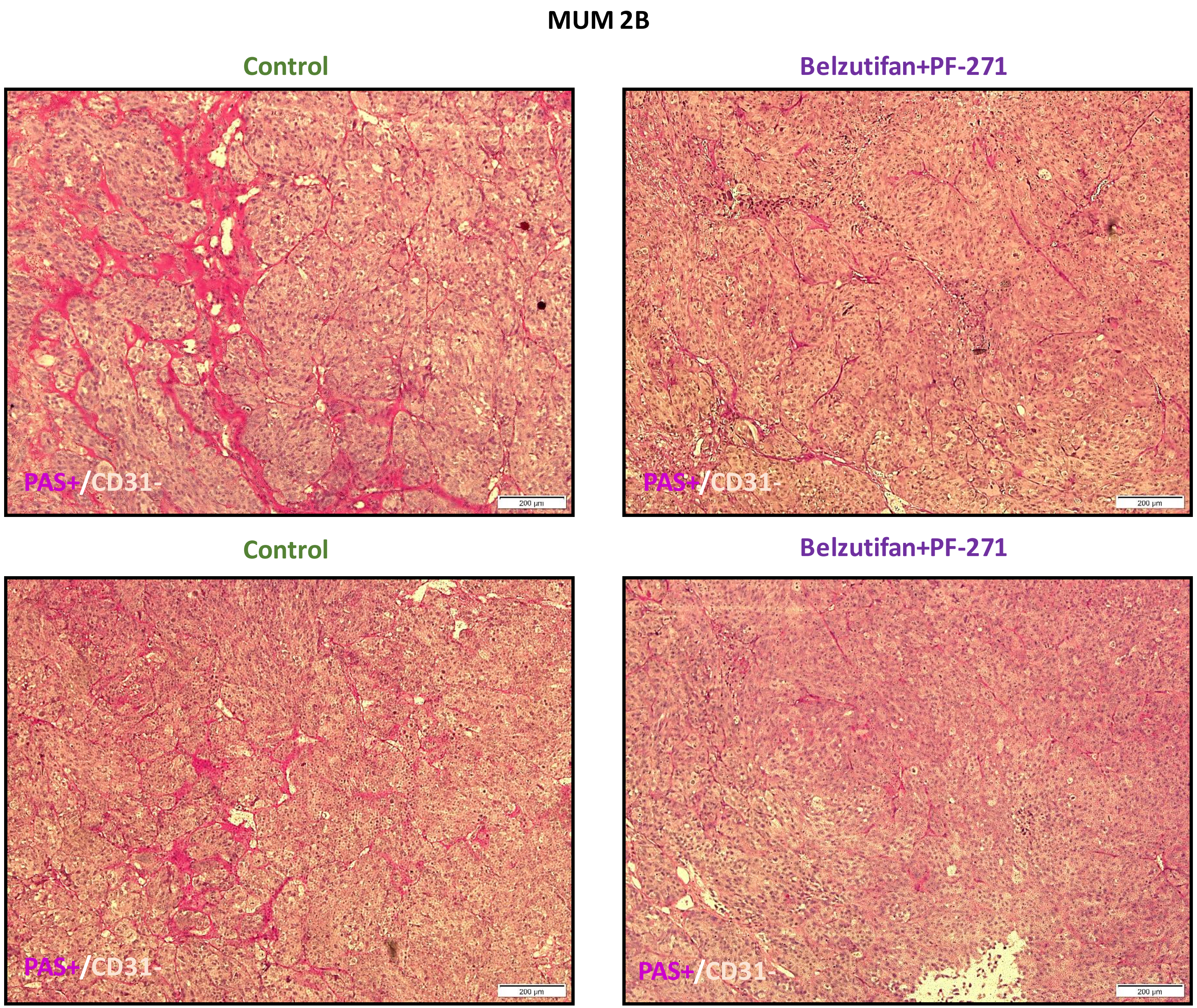
MUM 2B
Control
Belzutifan+PF-271
PAS+/CD31-
200 µm
PAS+/CD31-
200 µm
Control
Belzutifan+PF-271
PAS+/CD31-
200 µm
PAS+/CD31-
200 µm

Supplementary figure legends

Fig S1: Evaluation of the correlation between Ch3,8 normal vs Ch3 loss 8 gain uveal melanoma patients in VHL, PTK2 related genes: **A)** PDGFB, HIF-1AN, ANGPT2, LOXL2, VEGFA, FOXC2, S1PR1, TWIST1 and KDR, **B)** CCL5, CXCL10 and CCN2 respectively, mRNA levels in a cohort of uveal melanoma (n = 79, TCGA Firehose legacy) using cBioPortal database.

Fig S2: A) western blot experiments in MUM 2B after CNO (1 µM during 24 h) plus siVHL and **B)** MUM 2C cells (OE: overexposed). **C)** western blot experiments in MUM 2B after siPHD1, siPHD2 and siVHL. **D, E, J)** In vitro angiogenesis assay with Matrigel in MUM 2B showed the effect of siHIF2-alpha, siHIF-1 alpha, siPHD1, siPHD2, siVHL and CNO, images were acquired using an Olympus CKX41 microscope (10X lens) (bars 50 µm) and the formation of tube-like structures was then quantified by Wimasis program. **G-H)** western blot experiments and qPCR experiments with FAK-inhibitors, siHIF-2 alpha, siVHL (50 nM during 48 h) in MUM 2B and MUM 2C. Statistical analyses were conducted using Graph Pad Prism software. Statistical significance was calculated using a Student's t-test (unpaired, two-tailed) with measurements from at least three independent trials. **K)** overall survival in Control vs GNAQ or GNA11 mutant in n=79 cohort's uveal melanoma patients.

Fig S3: A) western blot experiments in RDES and SKNMC, chromosome 3,8 representation with deep deletion of VHL, Bap1 and amplification of Ch8 in SKNMC cells (cBioPortal database, Cancer Cell Line Encyclopedia), In vitro angiogenesis assay with Matrigel 3D and 2D, images were acquired using an Olympus CKX41 microscope (10X lens) (bars 50 µm). **B)** copy number alterations in cBioPortal database, Cancer Cell Line Encyclopedia from Ewing sarcoma cells. **C, D)** western blot experiments in MUM 2B, EW7, TC-71 and A673 cell lines after siPHD2 and **E)** in vitro angiogenesis assay with Matrigel, images were acquired using an Olympus CKX41 microscope (10X lens) (bars 50 µm). **F)** in vitro angiogenesis assay with Matrigel in uveal melanoma cells line, 92.1 and OMM-1.

Fig S4: in vitro angiogenesis assay with Matrigel in MUM 2B cell lines after siVHl and siPHD2, images were acquired using an Olympus CKX41 microscope (4X and10X lens) (bars 50 µm).

Fig S5: in vitro angiogenesis assay with Matrigel in MUM 2B or MUM 2C cell lines after **A)** hypoxia images were acquired using an Olympus CKX41 microscope (4X and10X lens) (bars 50 µm). **C)** Immunoprecipitation of VE-Cad after siVHL cytosol-nucleus fractionation of VE-Cadherin in MUM 2B cells. **D)** qPCR experiments with siVHL (50 nM during 48 h) in MUM 2B. Statistical analyses were conducted using Graph Pad Prism software. Statistical significance was calculated using a Student's t-test (unpaired, two-tailed) with measurements from at least three independent trials.

Fig S6: A, B) in vitro angiogenesis assay with Matrigel in MUM 2B cell lines after siPHD2 or siVHL plus Belzutifan and PF-271 (1 µM during 24 h), images were acquired using an Olympus CKX41 microscope (4X and10X lens) (bars 50 µm).

Fig S7: A) Immunohistochemistry analysis is performed to observe HIF-2 alpha and p-FAK expression from xenografts experiments. Consecutive sections are shown. Bars: 200µm. **B)** PAS/CD31 Immunohistochemistry analysis is performed to observe VM regions in MUM 2B and MUM 2C xenograft experiments, Consecutive sections are shown. Bars: 200µm. The percentage of immunostained tumor cells was scored as follows: 0%, 0: negative; <19%, 1: weak, >20%, 2: middle and >50%, 3: positive quantified by pathologist. Representative images were acquired in a microscope (Olympus BX-61).

Fig S8: PAS Immunohistochemistry analysis is performed to observe PAS+/CD31- regions in MUM 2B xenograft experiments, Consecutive sections are shown. Bars: 200µm. Quantified by pathologist the PAS+/CD31- regions. Representative images were acquired in a microscope (Olympus BX-61).
