# Supplementary material for: Chromosomal 3p loss and 8q gain drive vasculogenic mimicry via HIF-2α and VE-cadherin activation in uveal melanoma
Source: Cell Death Differ. 2025 Feb 26;32(8):1473–83. doi: 10.1038/s41418-025-01469-9 (PMC12325912; doi:10.1038/s41418-025-01469-9)

01/03/2024

MUM2B

Cont CNO 1h

FAK Y397

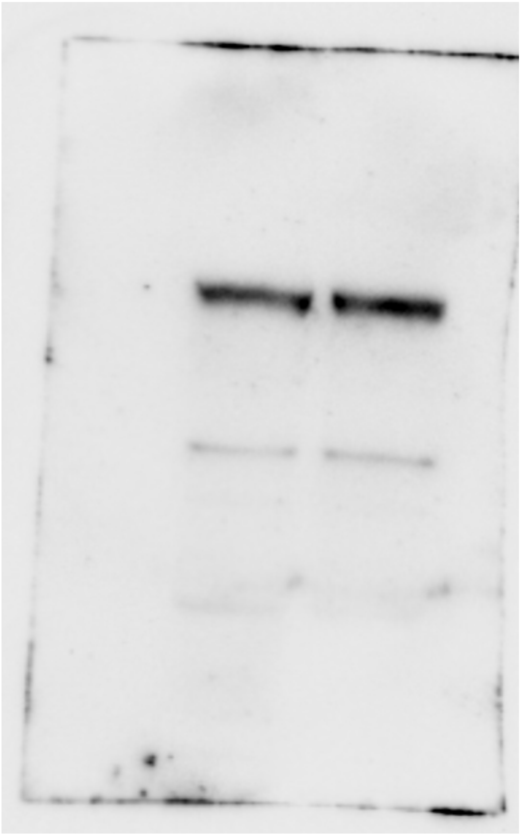

05/03/2024

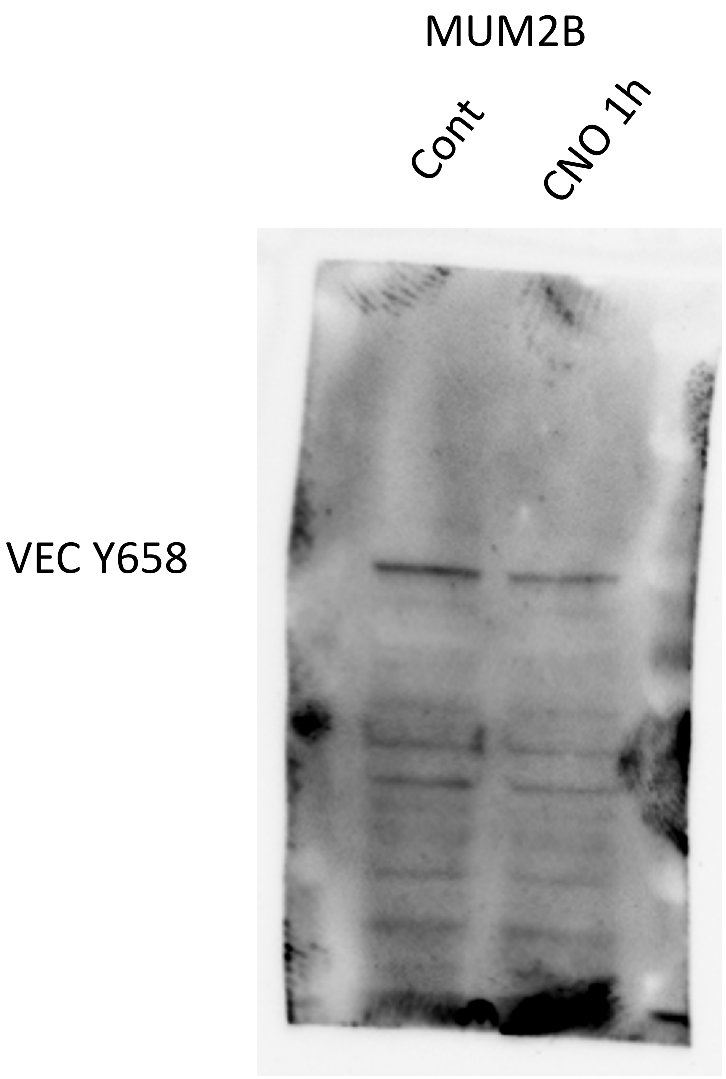

05/03/2024

MUM2B

Cont CNO 1h

Tubulina

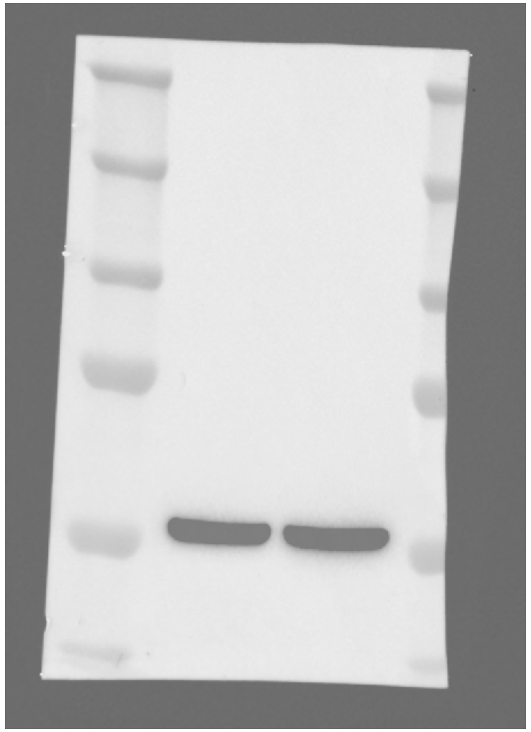

05/03/2024

MUM2C

Cont CNO

scb siVHL scb siVHL

HIF2a

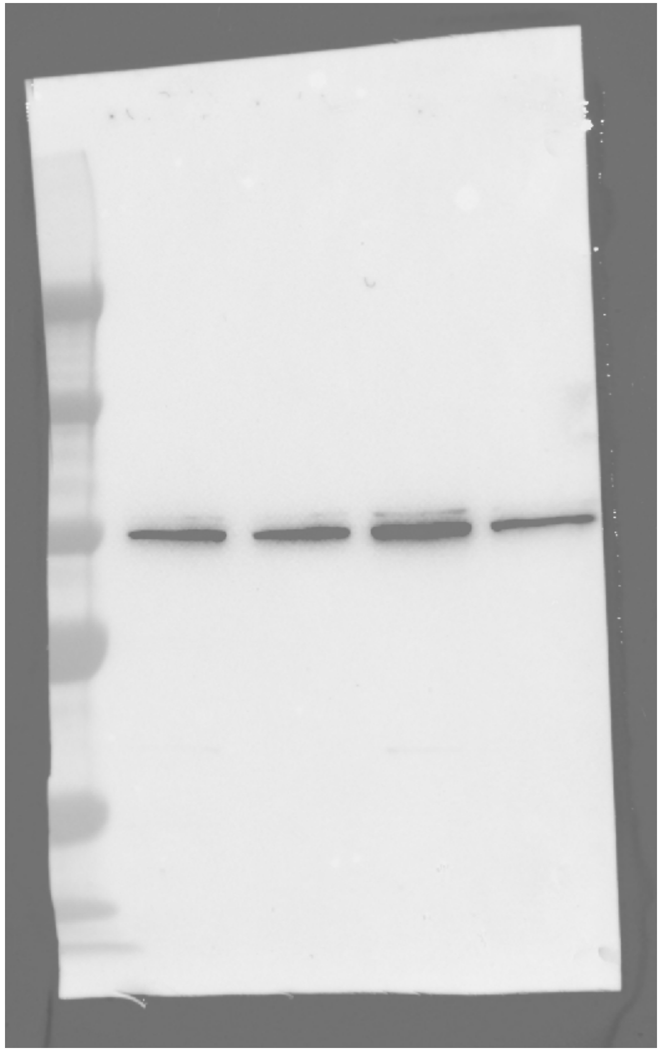

06/03/2024

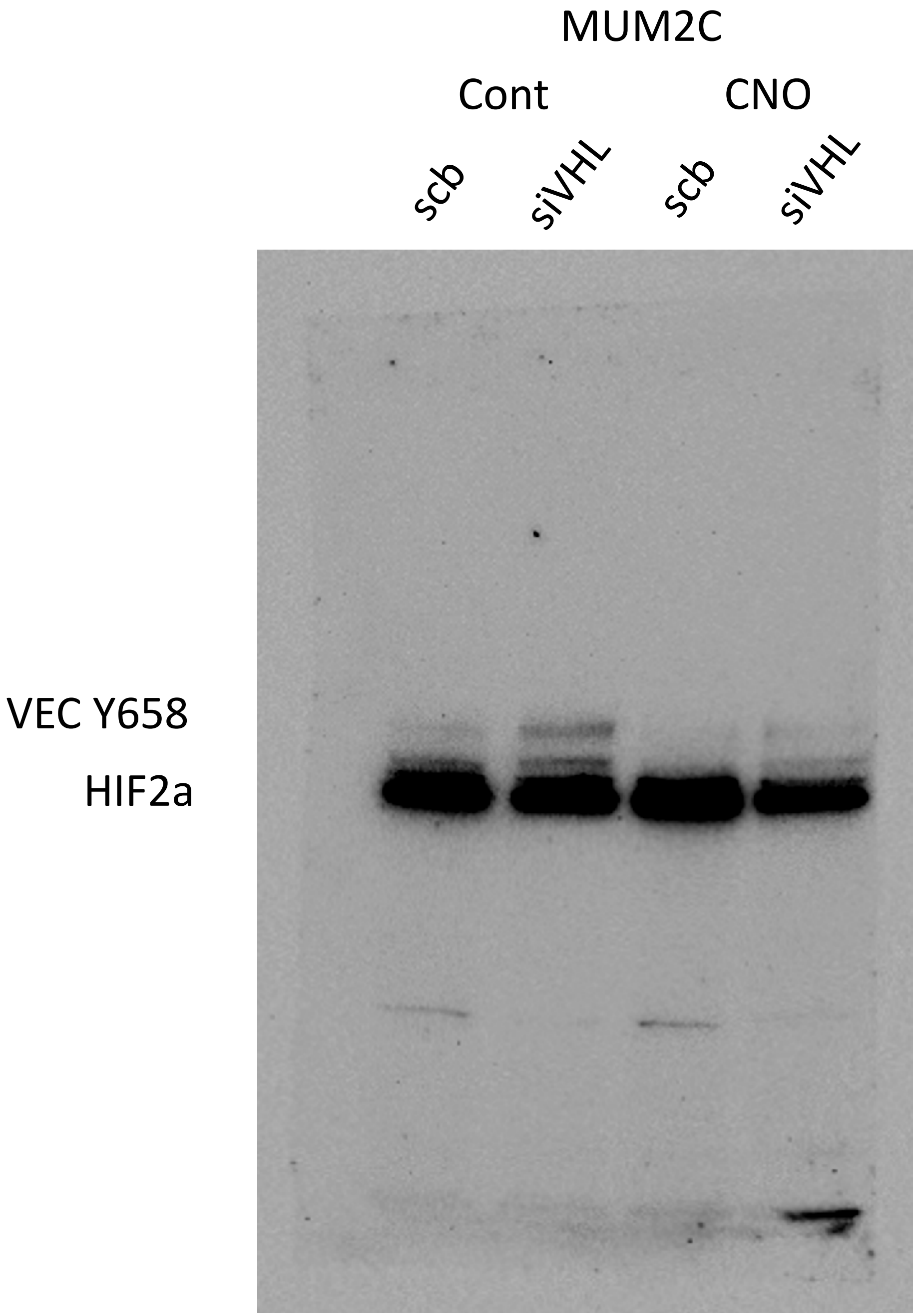

06/03/2024

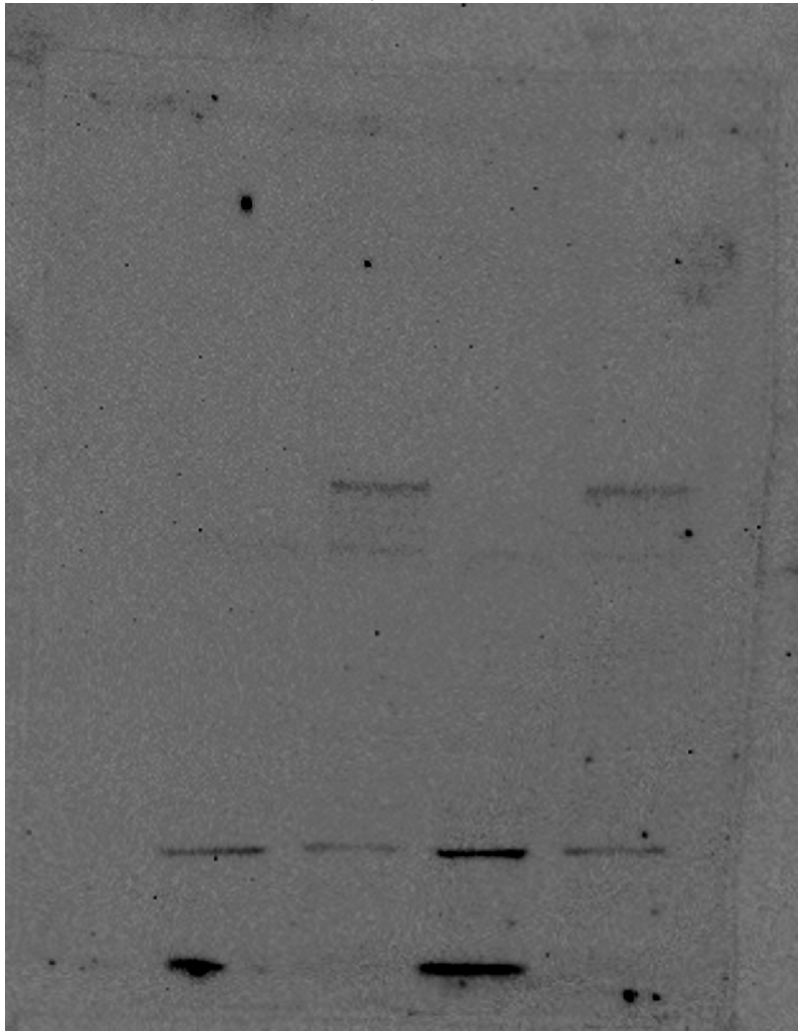

07/03/2024

MUM2C

Cont CNO

scb siVHL scb siVHL

HIF1a

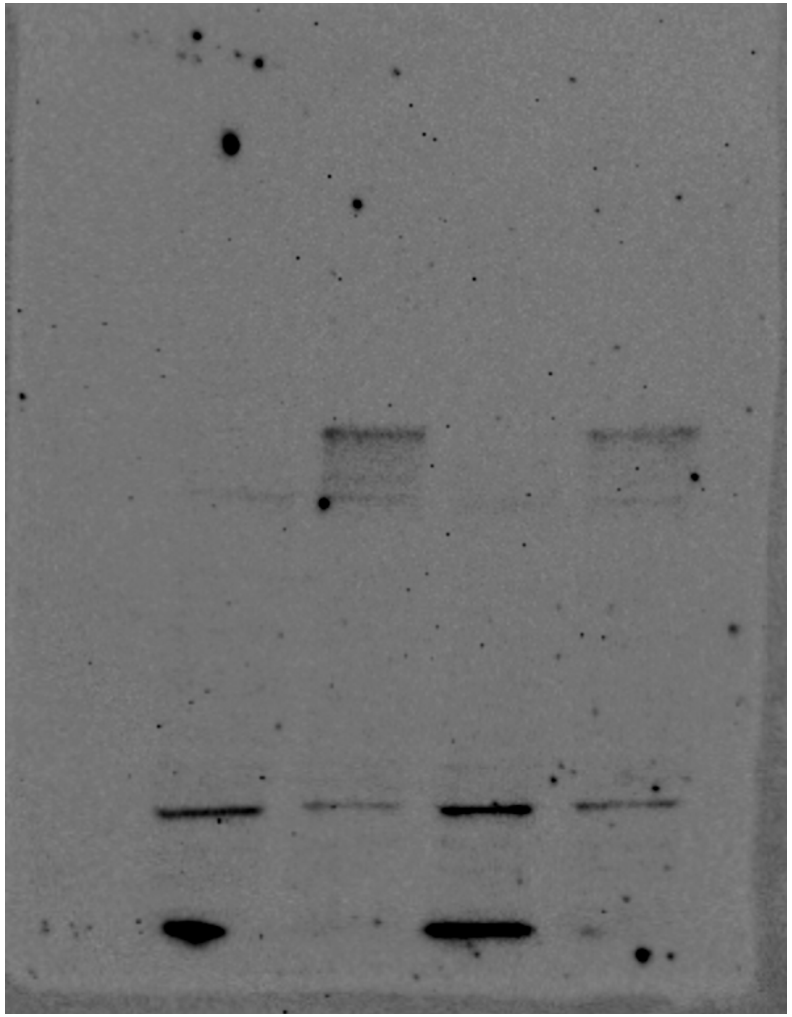

07/03/2024

MUM2C

Cont CNO

scb siVHL scb siVHL

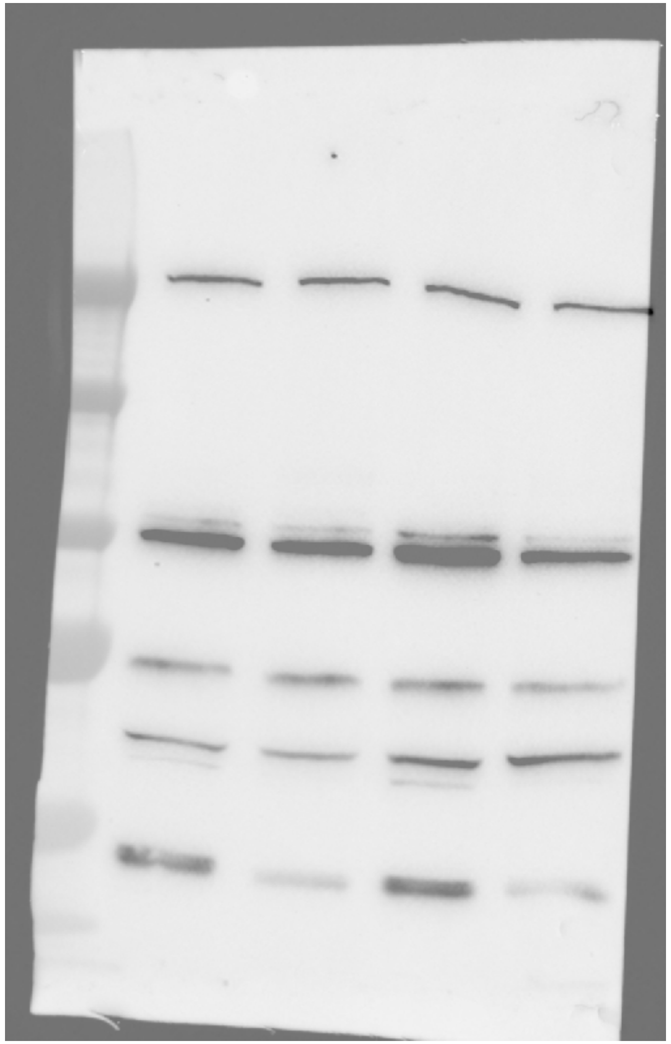

PHD2

12/03/2024

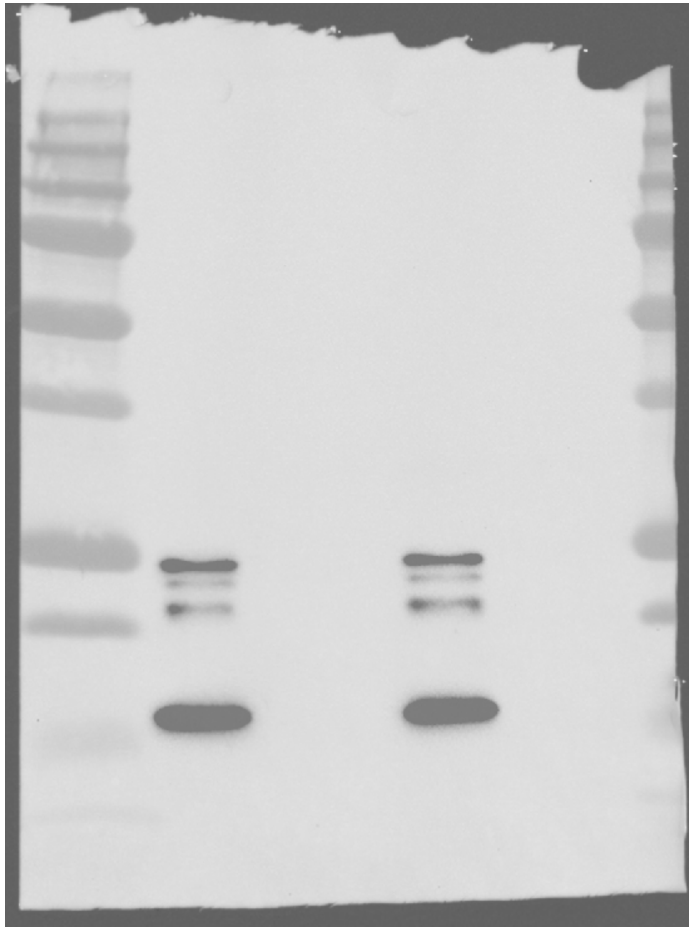

12/03/2024

MUM2B MUM2C

Cont CNO 20h Cont CNO 20h

VEC Y658

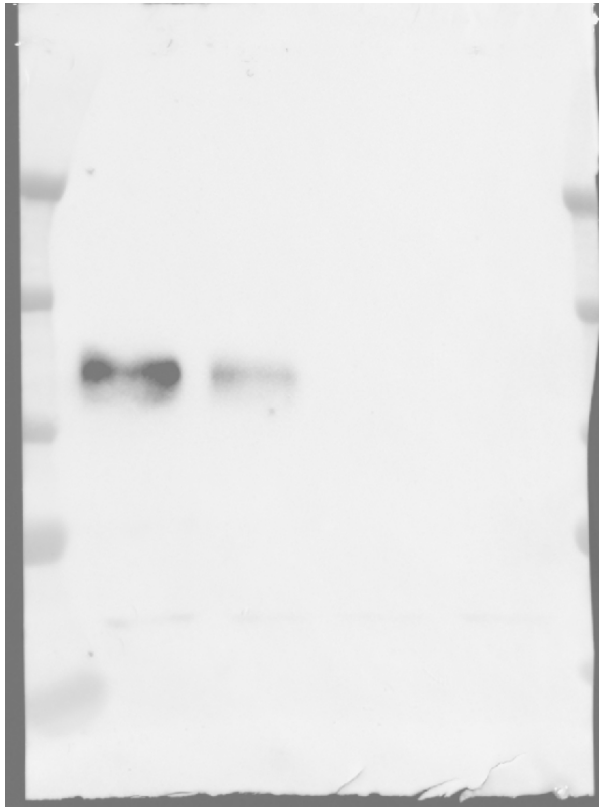

12/03/2024

MUM2B MUM2C

Cont CNO 20h Cont CNO 20h

FAK Y397

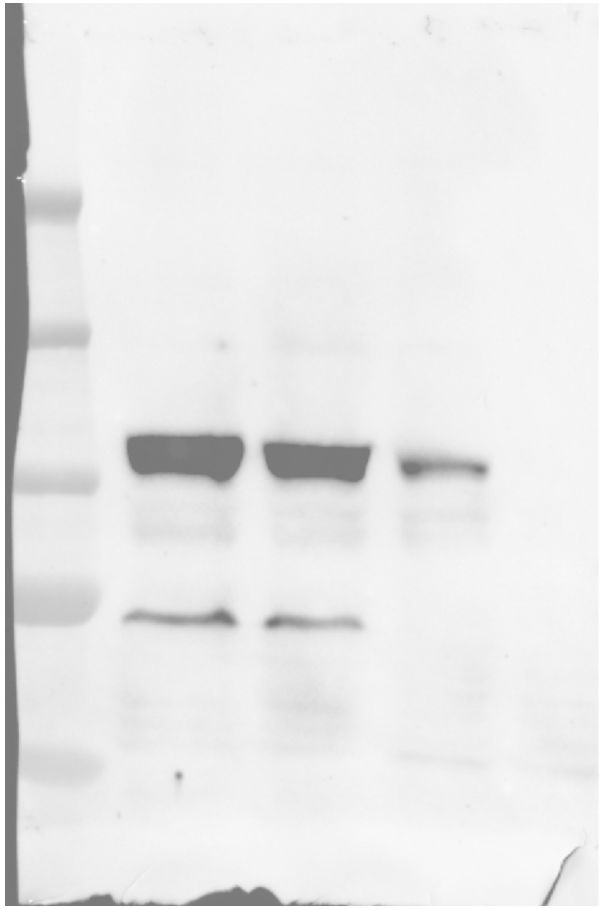

13/03/2024

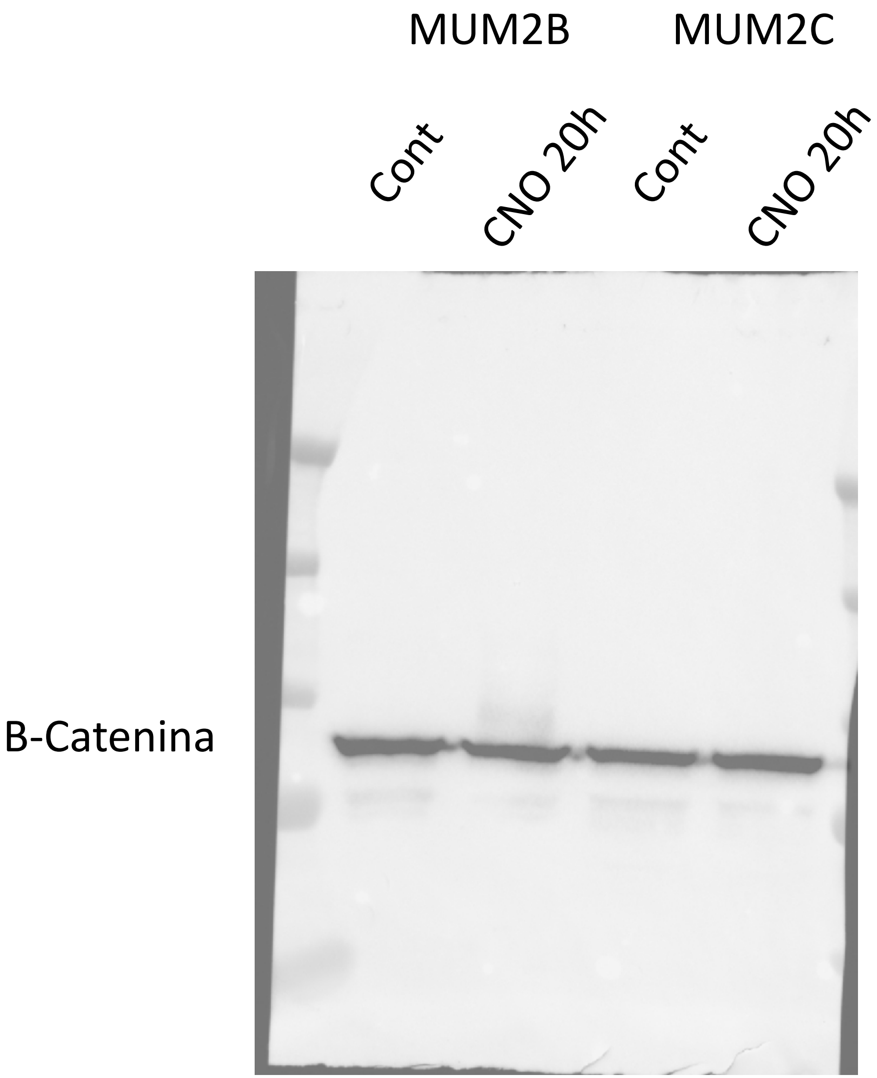

13/03/2024

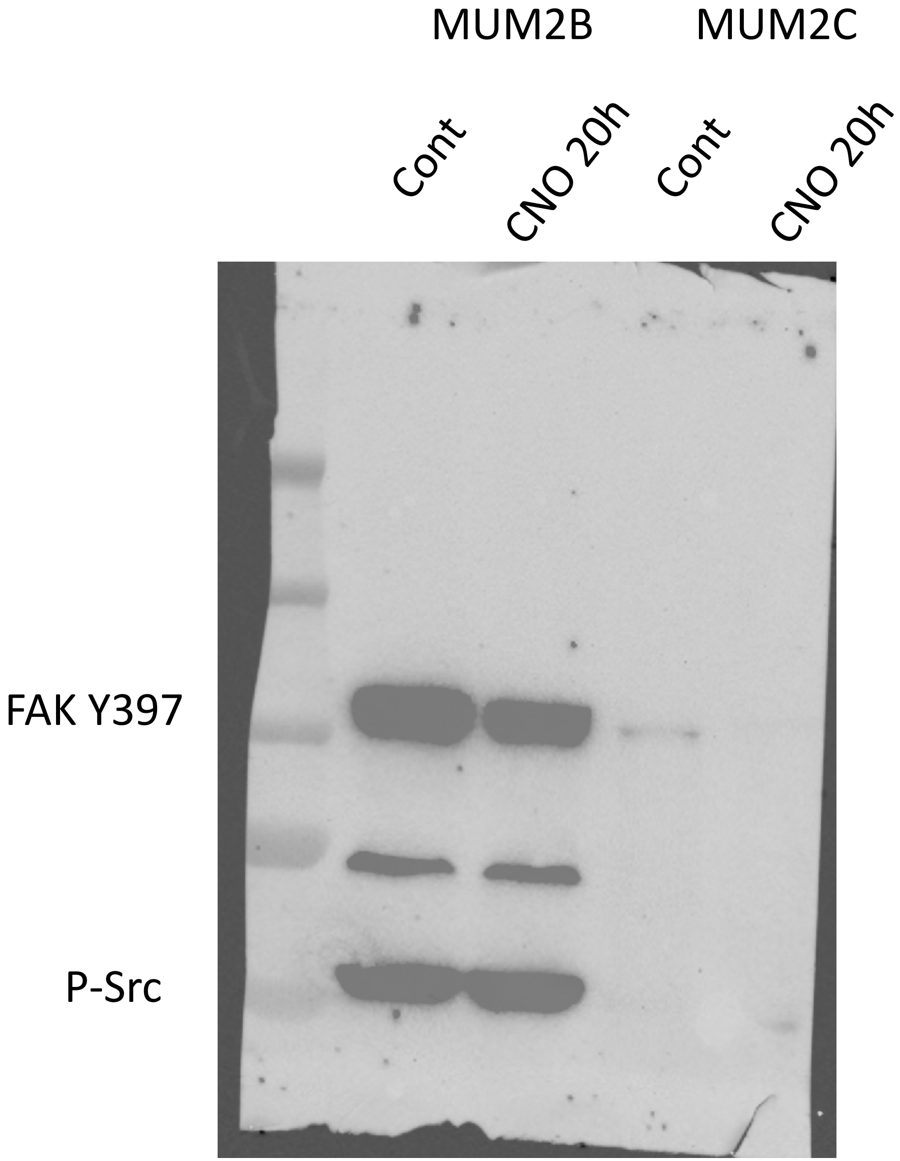

13/03/2024

MUM2B MUM2C

Cont CNO 20h Cont CNO 20h

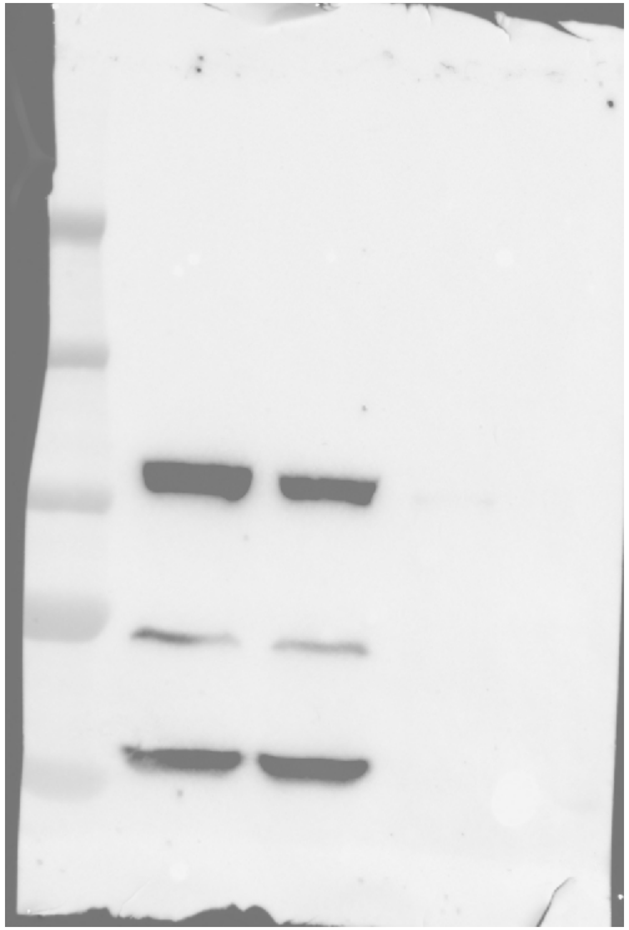

FAK Y397

P-Src

14/03/2024

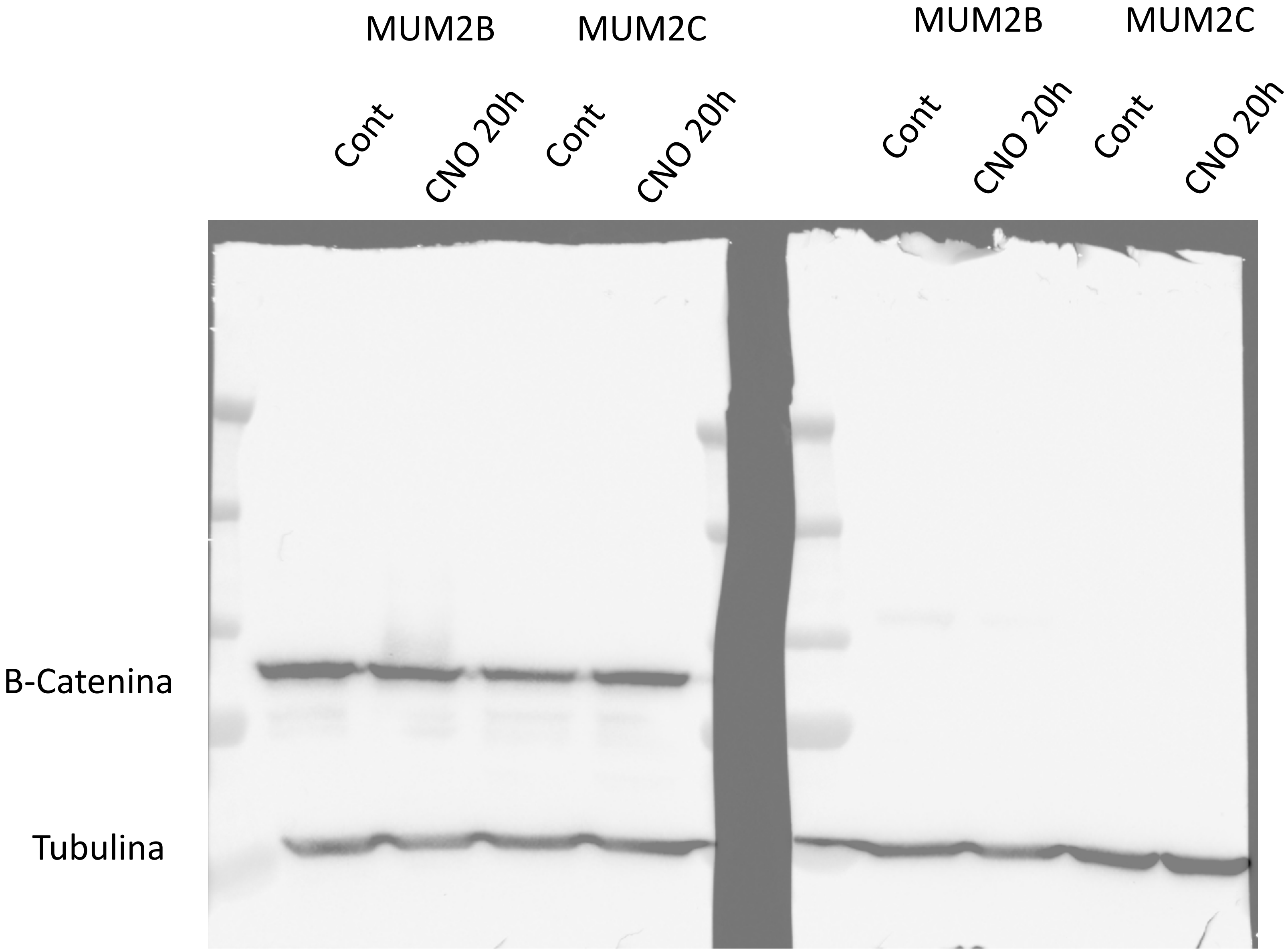
MUM2B
MUM2C
MUM2B
MUM2C
Cont
CNO 20h
Cont
CNO 20h
Cont
CNO 20h
Cont
CNO 20h
B-Catenina
Tubulina

14/03/2024

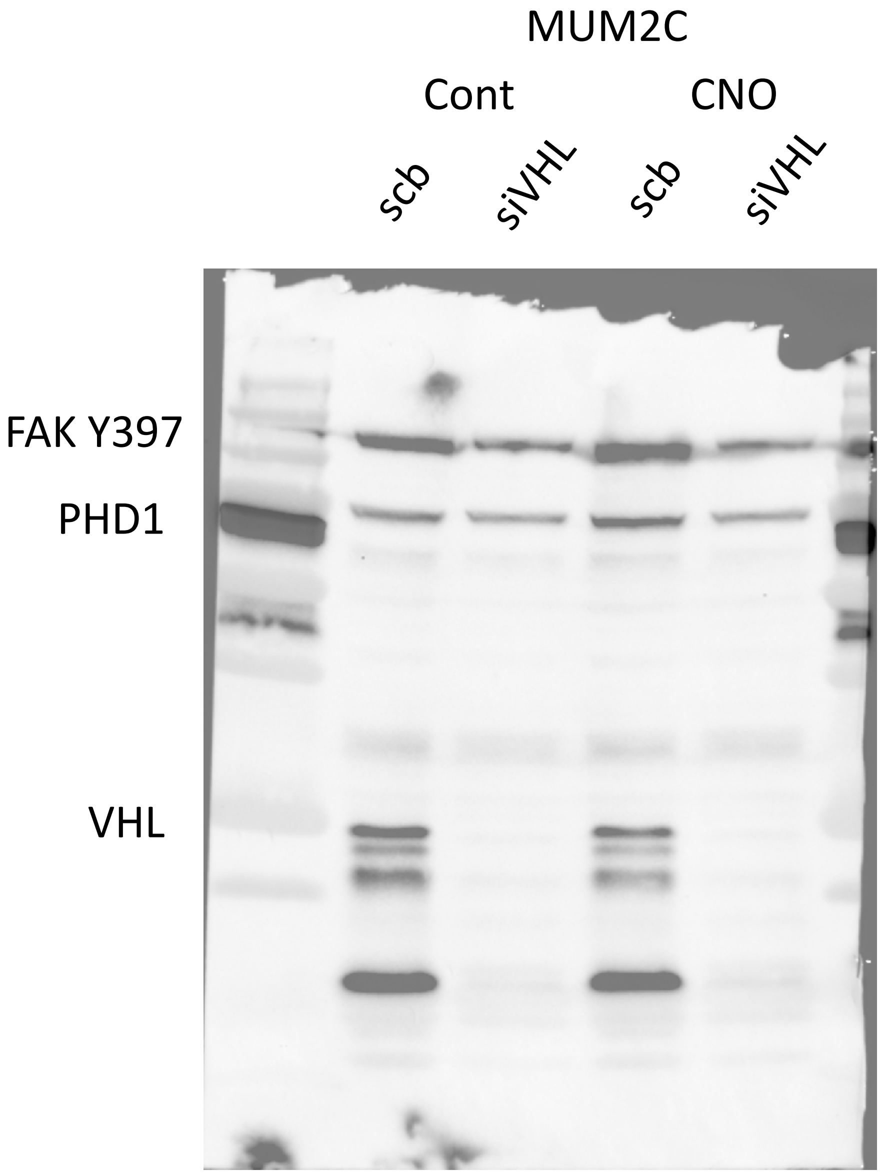

14/03/2024

MUM2C

Cont CNO

scb siVHL scb siVHL

FAK

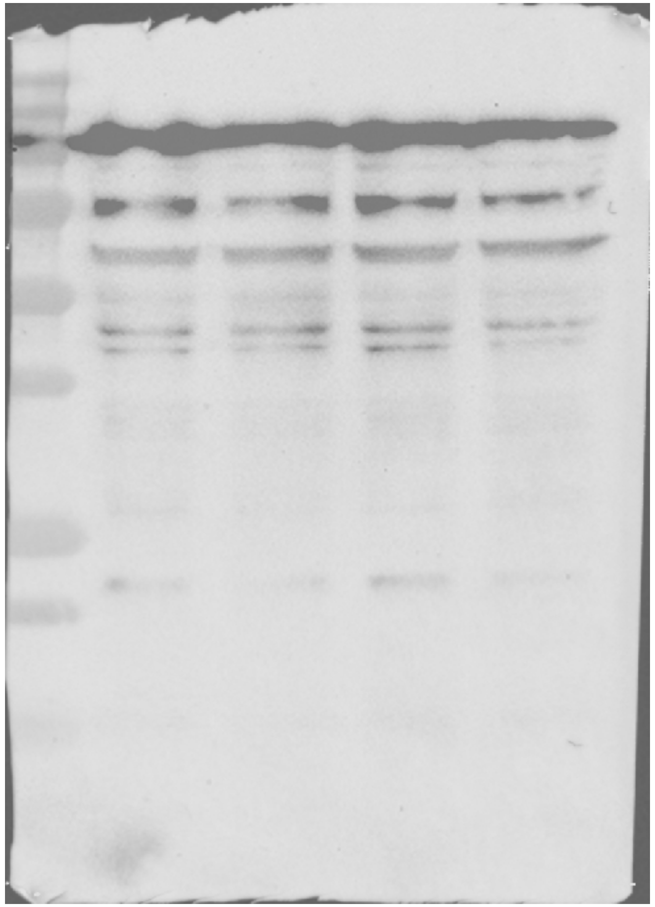

15/03/2024

MUM2C

Cont CNO

scb siVHL scb siVHL

Tubulina

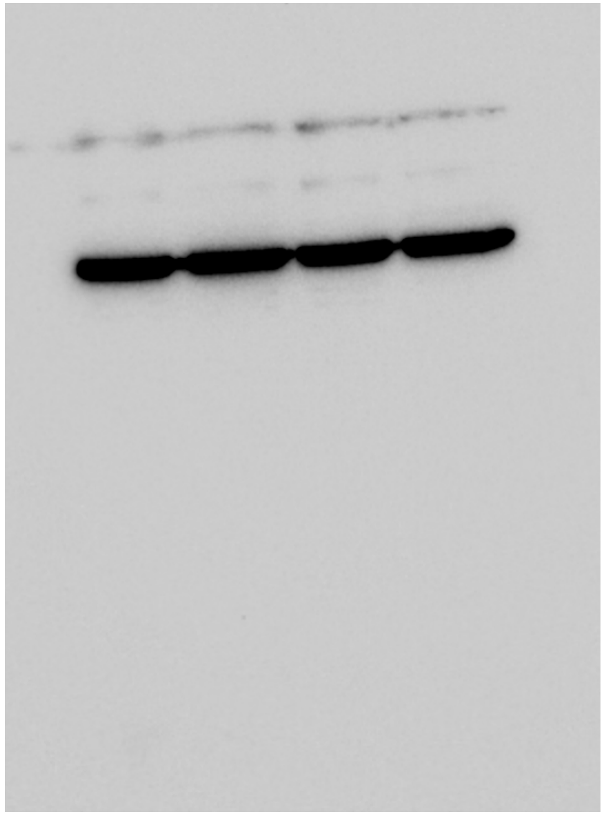

03/04/2024

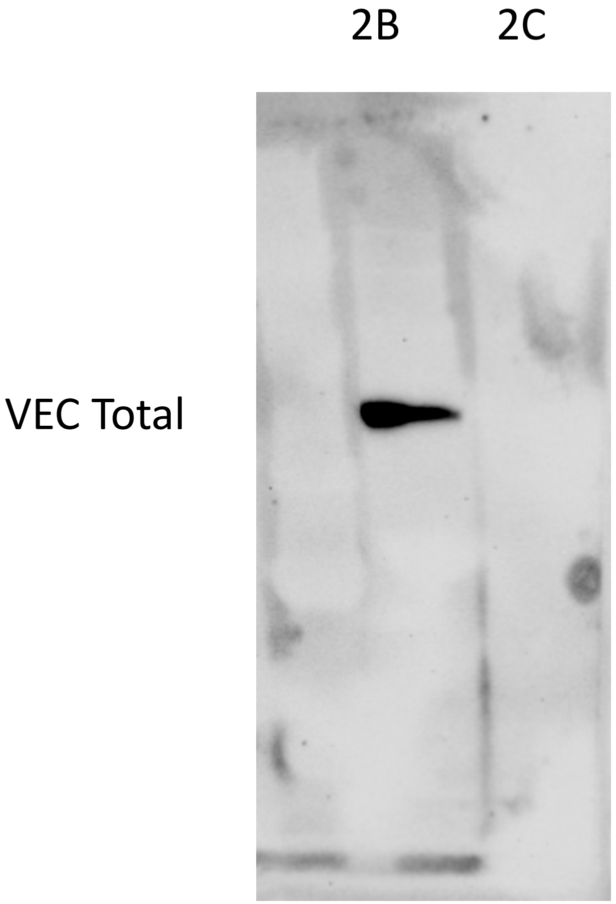

03/04/2024

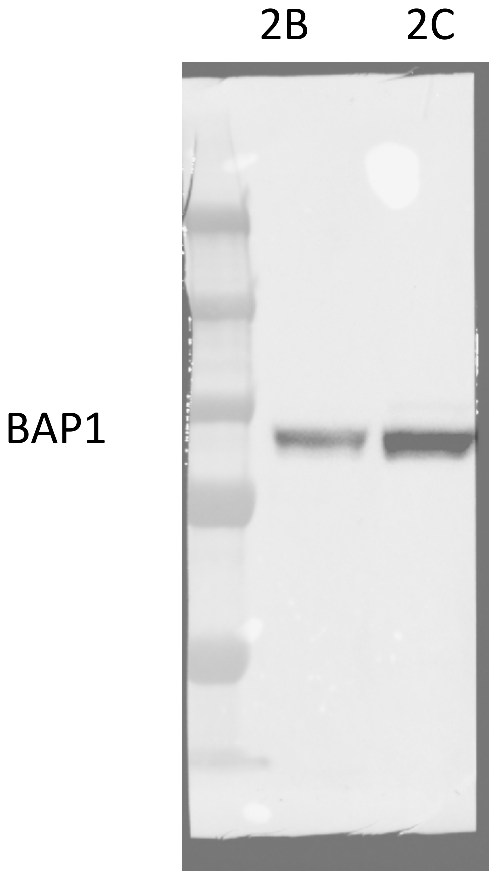

03/04/2024

MUM2B IP b-catenina

scb | siVHL | scb + CNO | siVHL + CNO

CE NE CE NE CE NE CE NE IgG NE

VEC Total

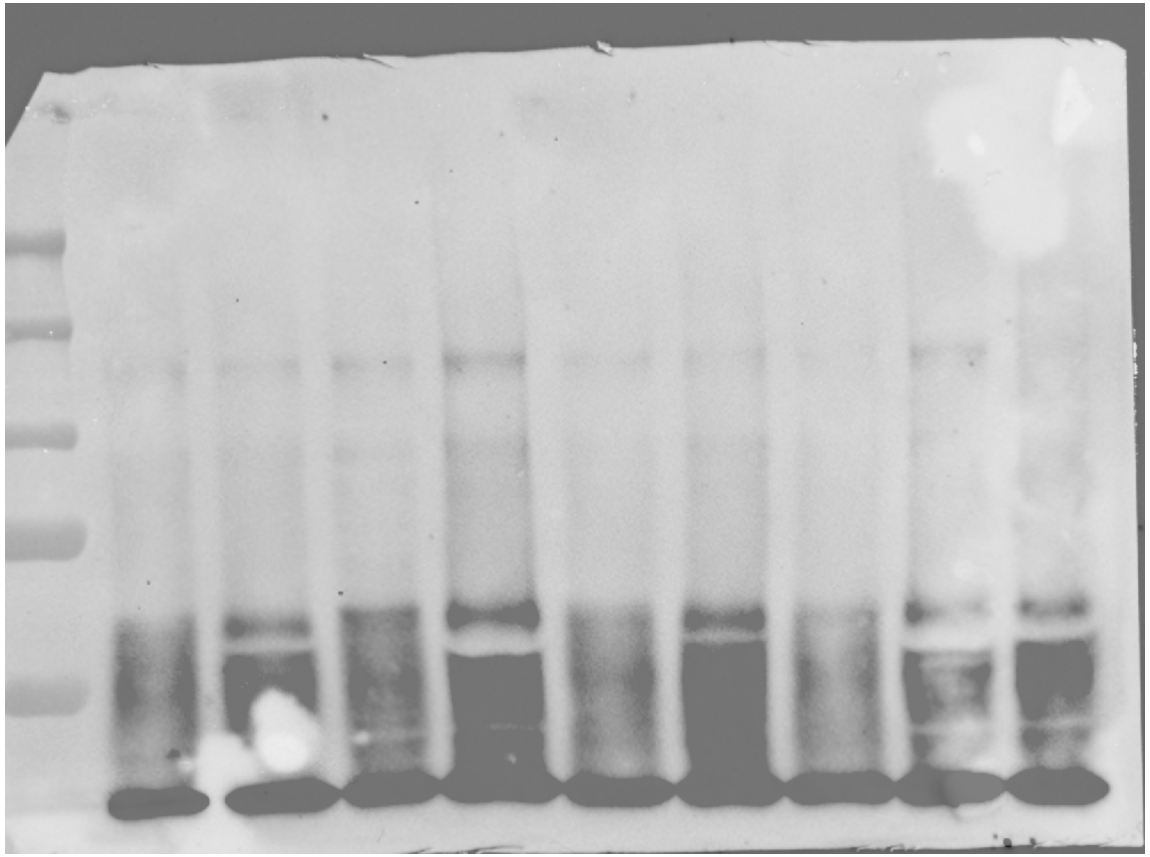

03/04/2024

MUM2B

scb siVHL siPHD1 siPHD2 siPHIF1 siHIF2

Y658 VEC

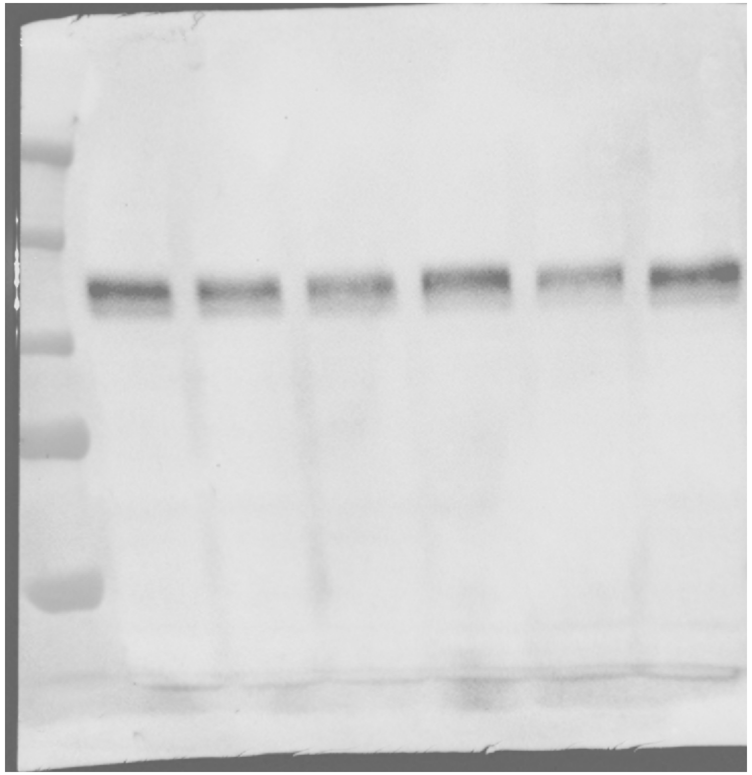

04/04/2024

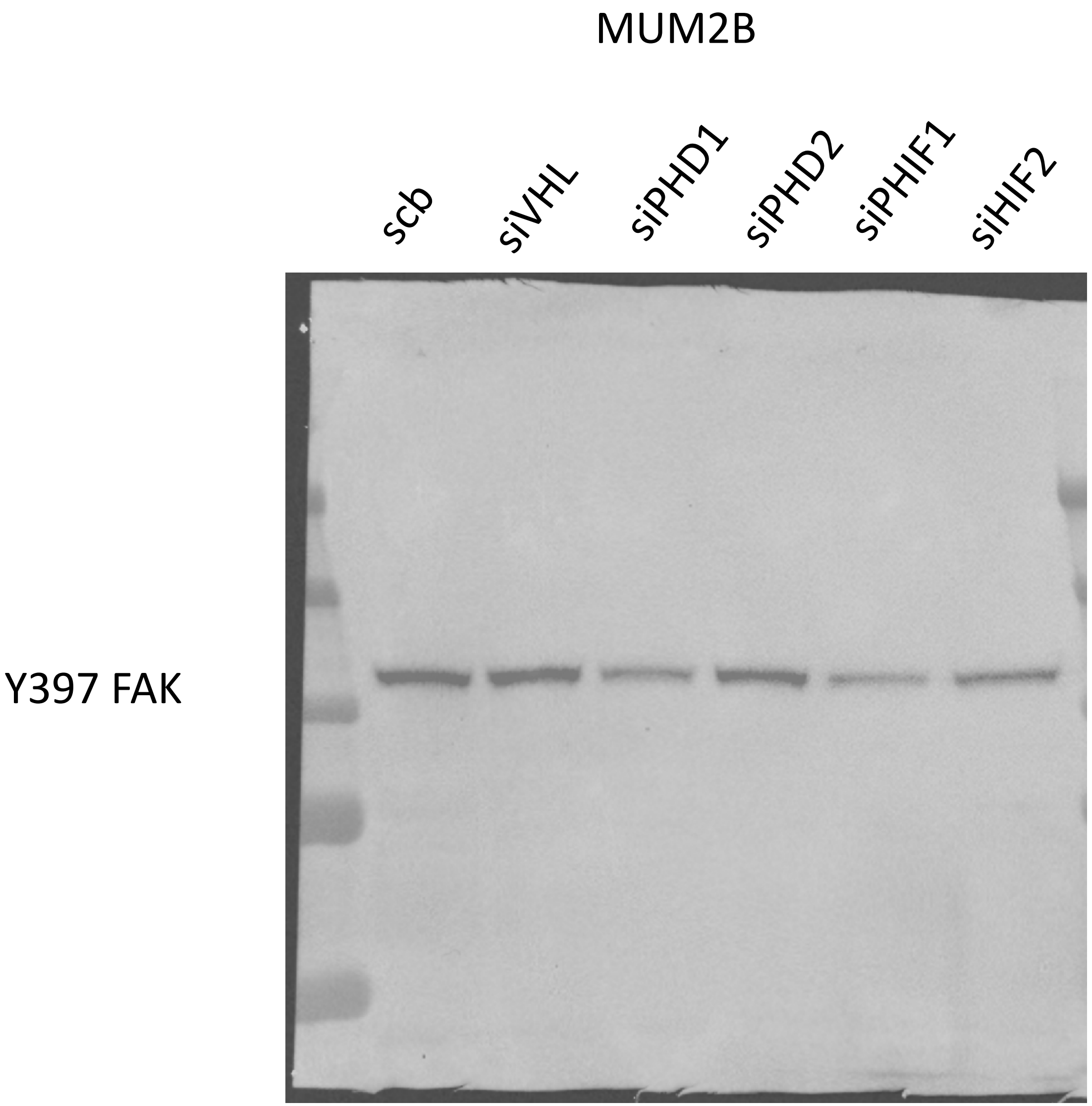

04/04/2024

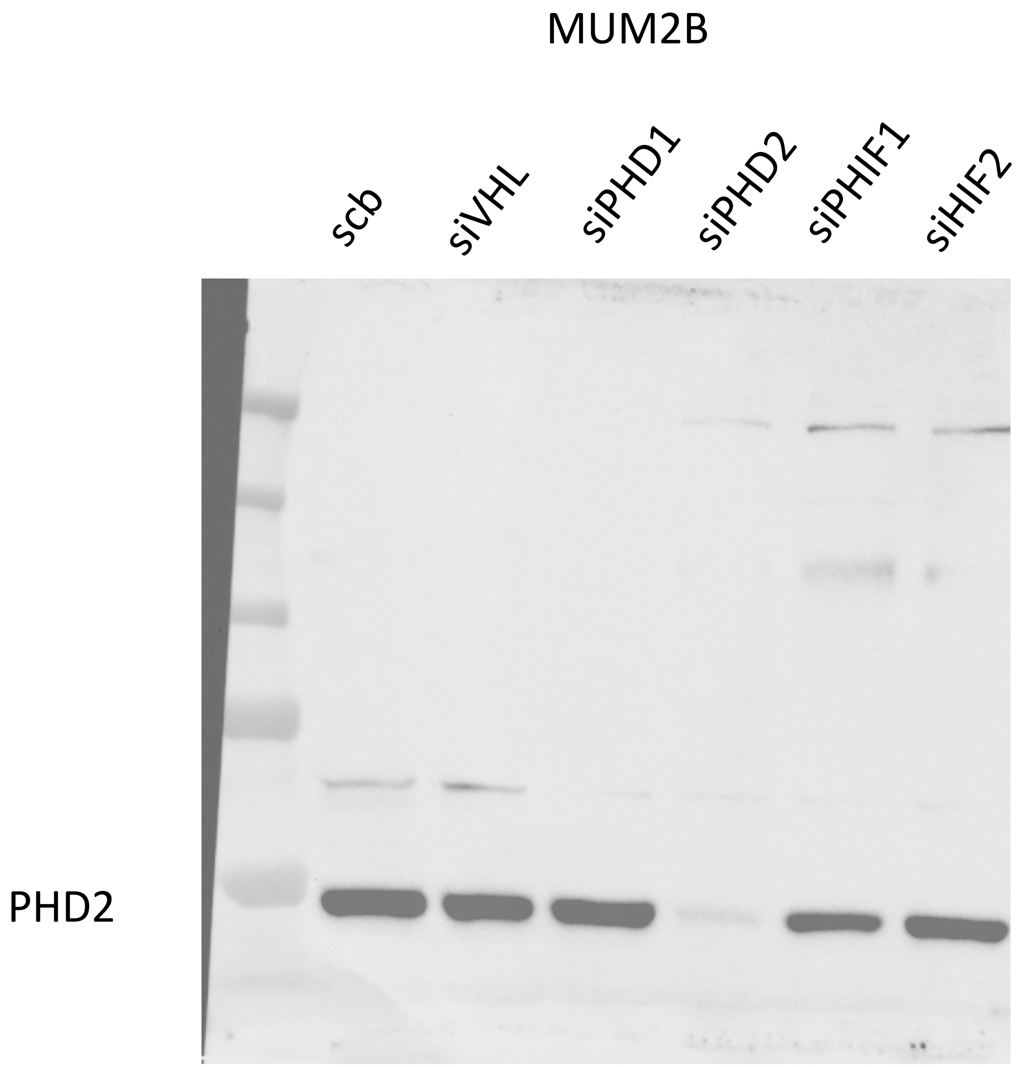

04/04/2024

VEC Total Y397 FAK

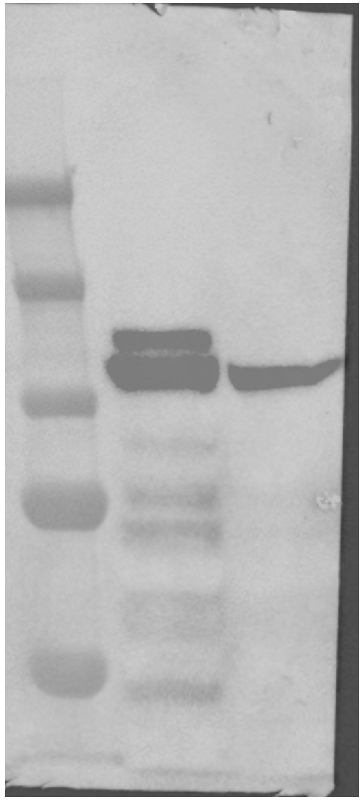

04/04/2024

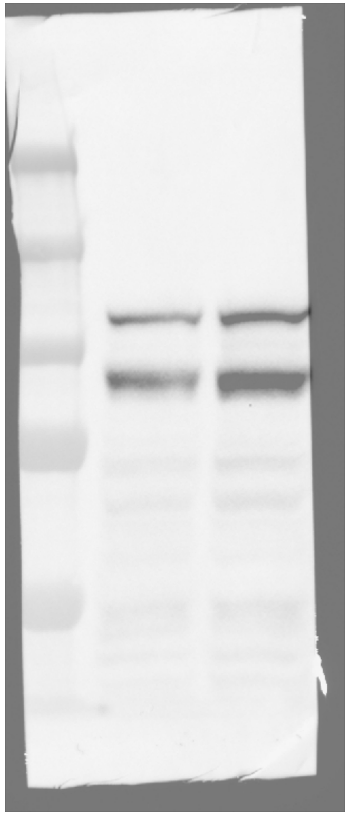

04/04/2024

MUM2B IP b-catenina

scb siVHL scb + CNO siVHL + CNO

CE NE CE NE CE NE CE NE IgG NE

B-catenina

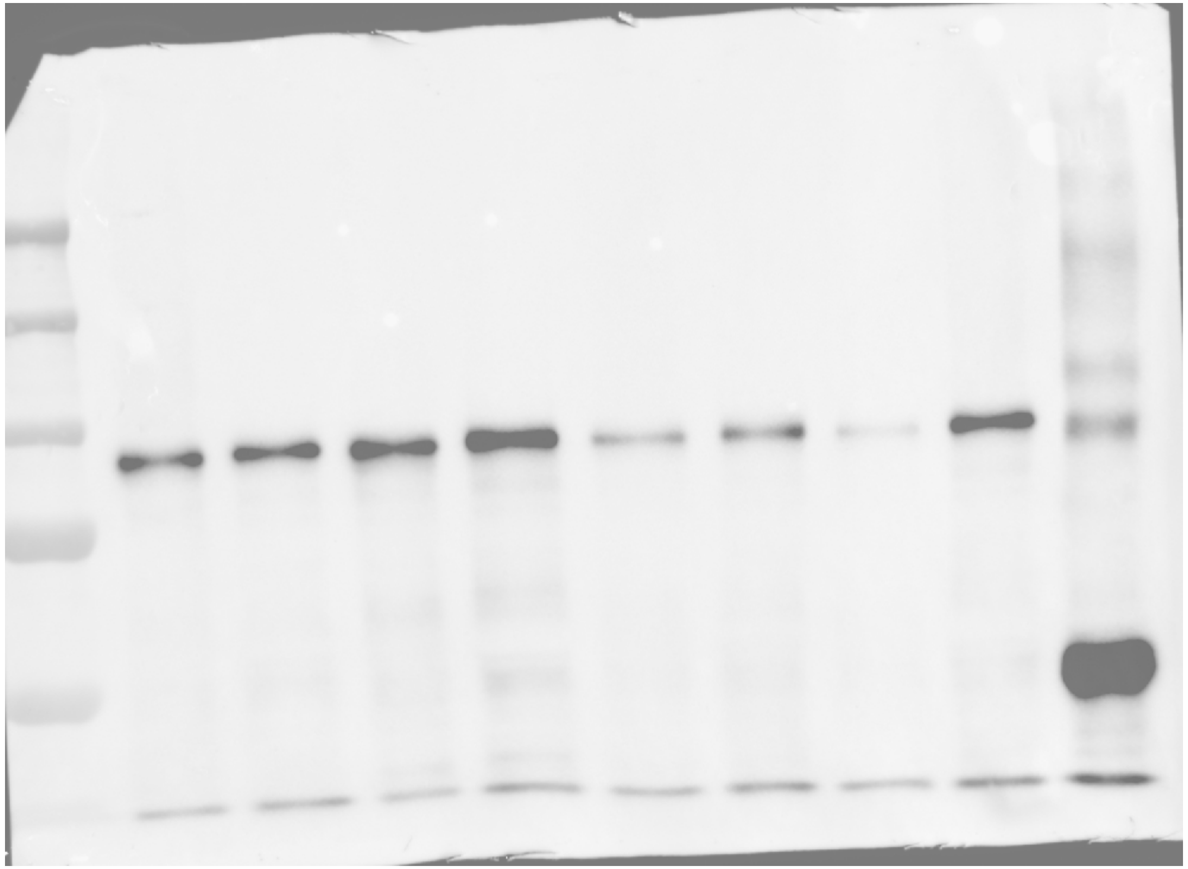

04/04/2024

MUM2C IP b-catenina

scb | siVHL | scb + CNO | siVHL + CNO

CE NE CE NE CE NE CE NE IgG NE

Y658 VEC

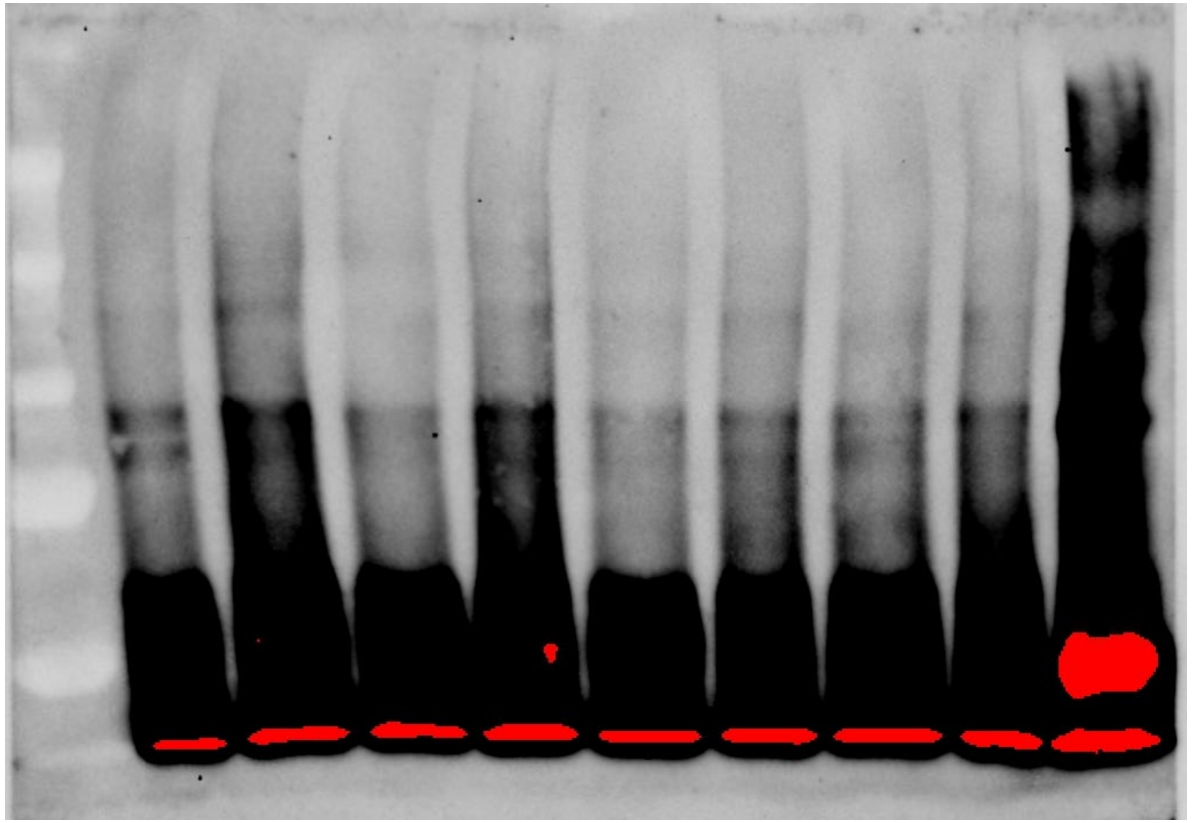

05/04/2024

MUM2B

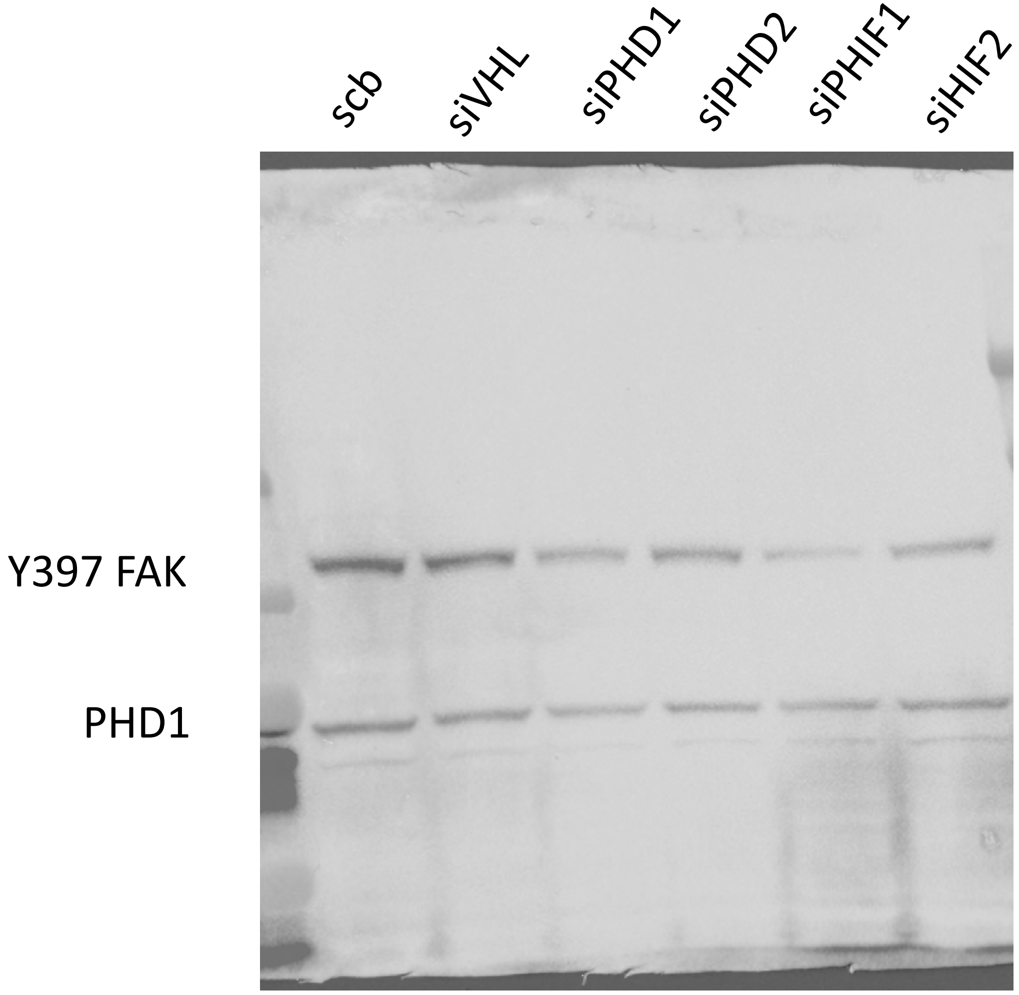

05/04/2024

MUM2B

scb siVHL siPHD1 siPHD2 siPHIF1 siHIF2

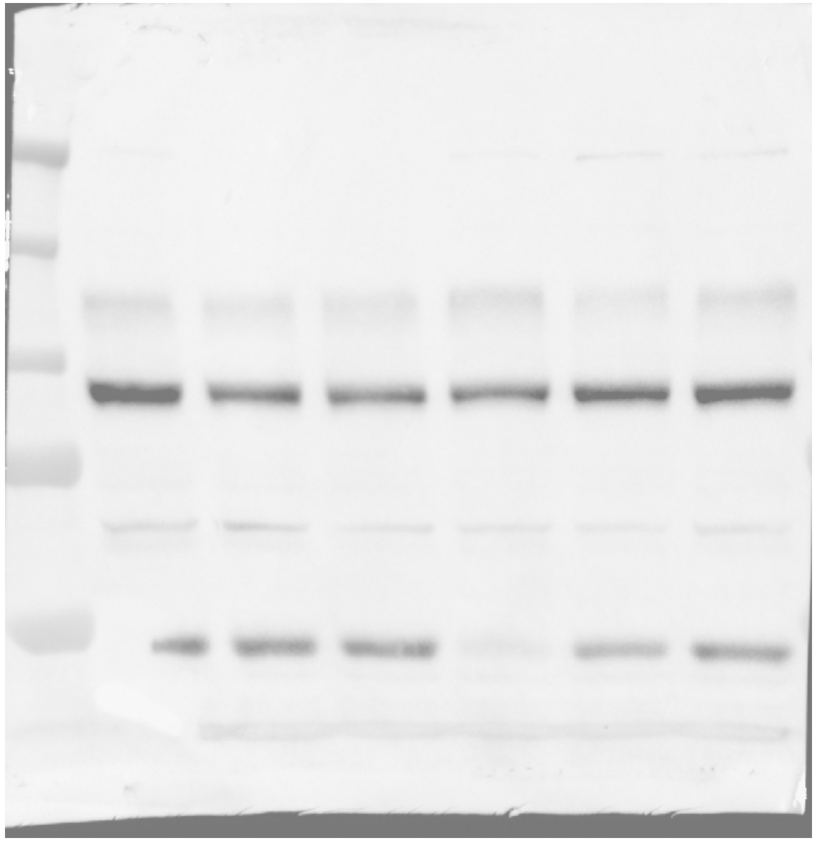

BAP1

PHD2

05/04/2024

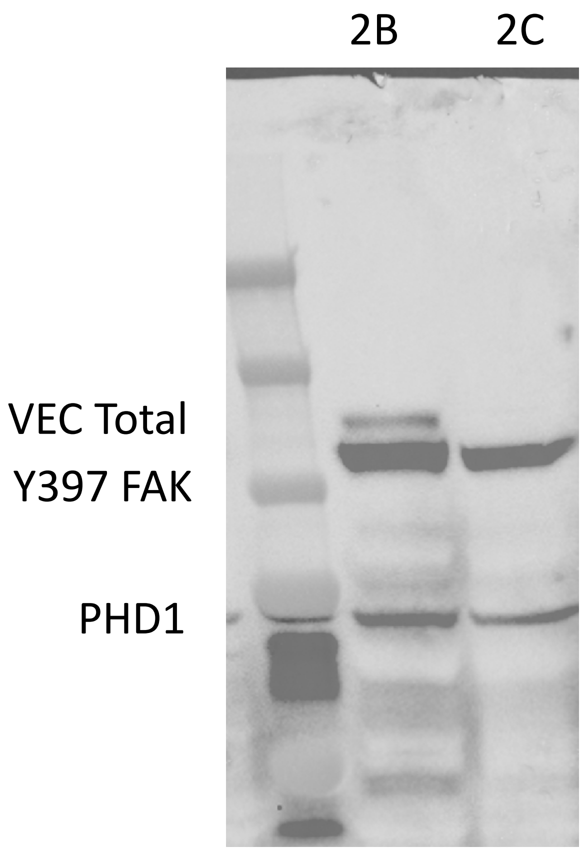

05/04/2024

2B 2C

FAK Total

BAP1

P-Src

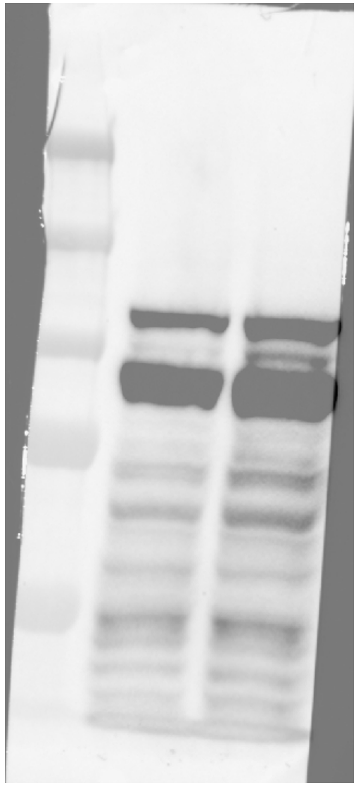

05/04/2024

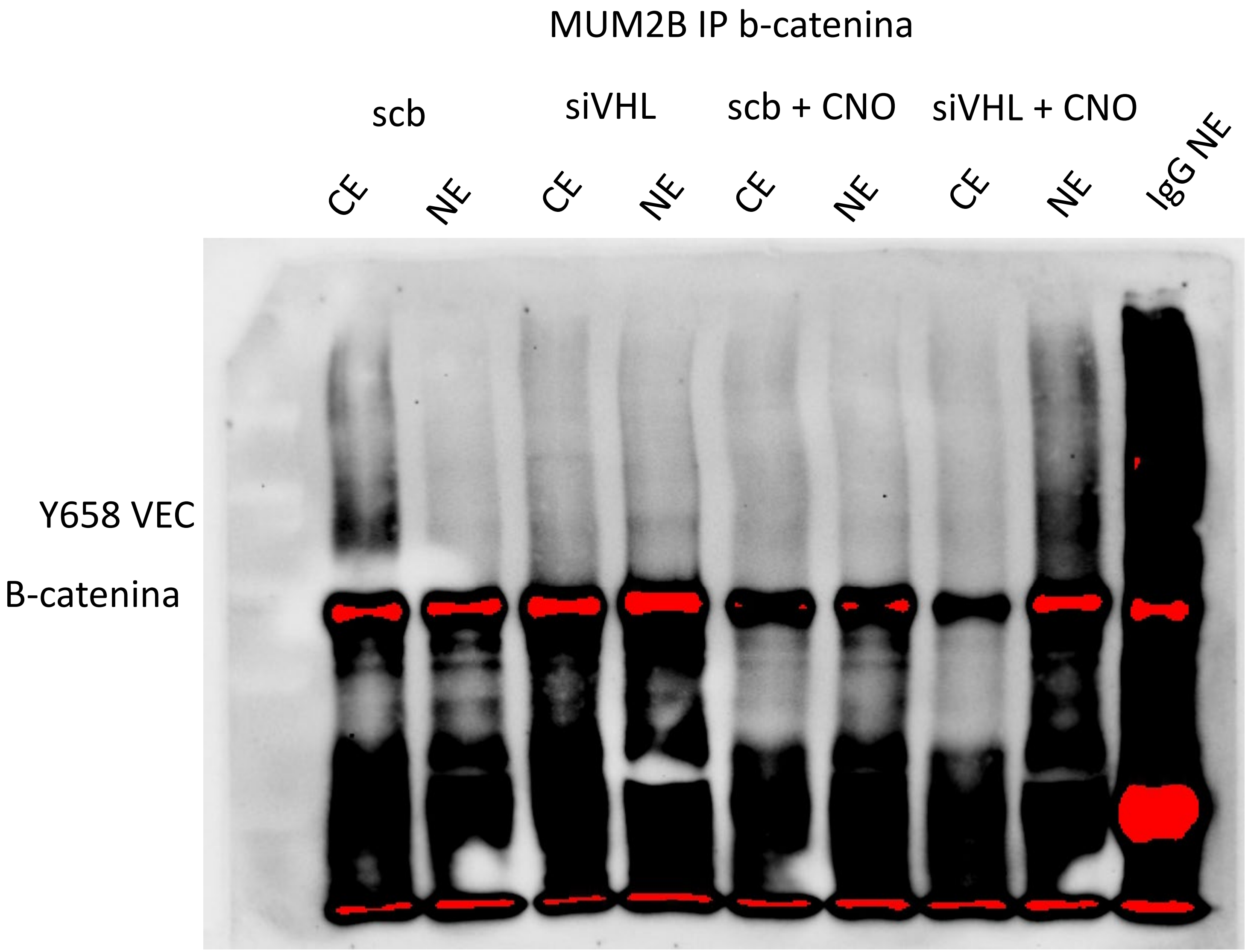

MUM2B IP b-catenina
scb
siVHL
scb + CNO
siVHL + CNO
CE
NE
CE
NE
CE
NE
CE
NE
IgG NE
Y658 VEC
B-catenina

05/04/2024

MUM2C IP b-catenina

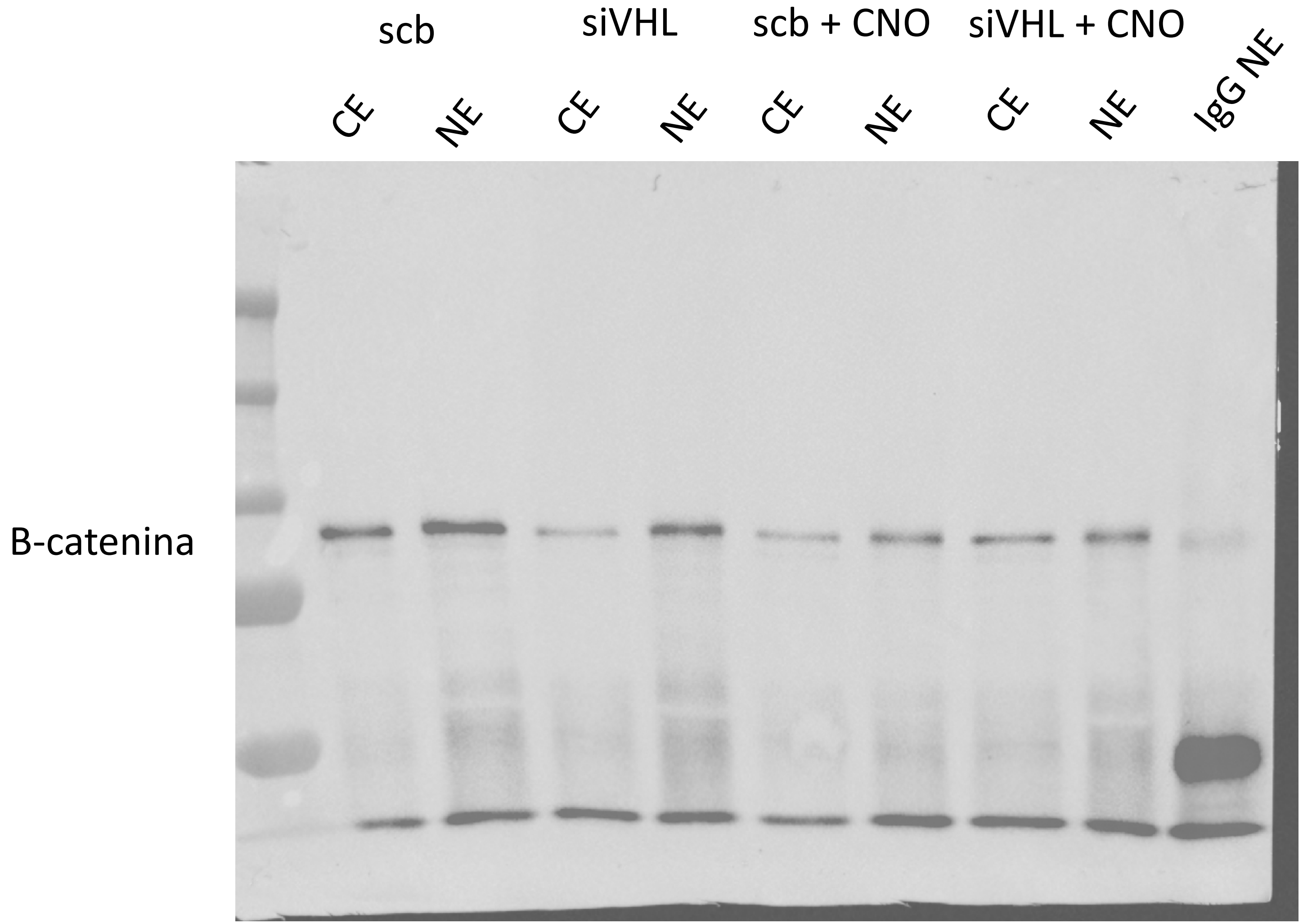

09/04/2024

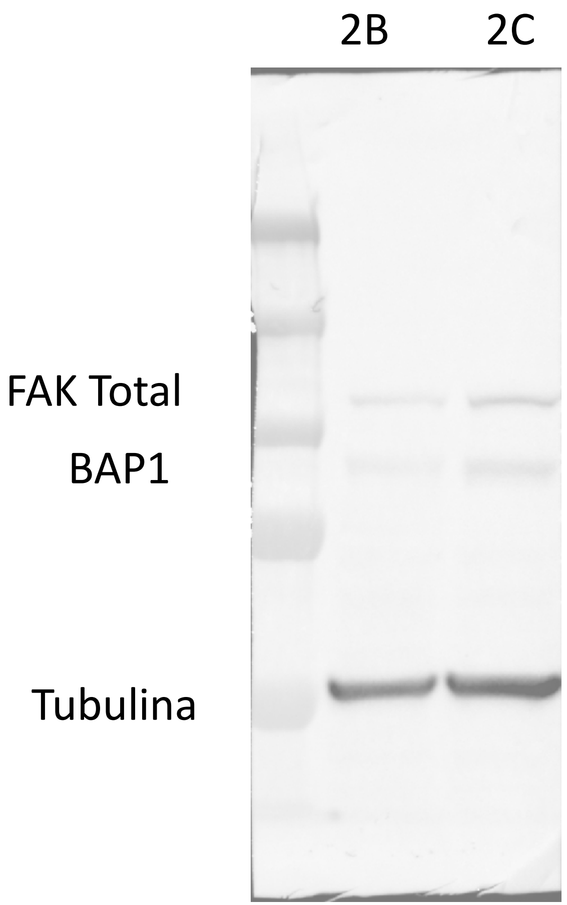

10/04/2024

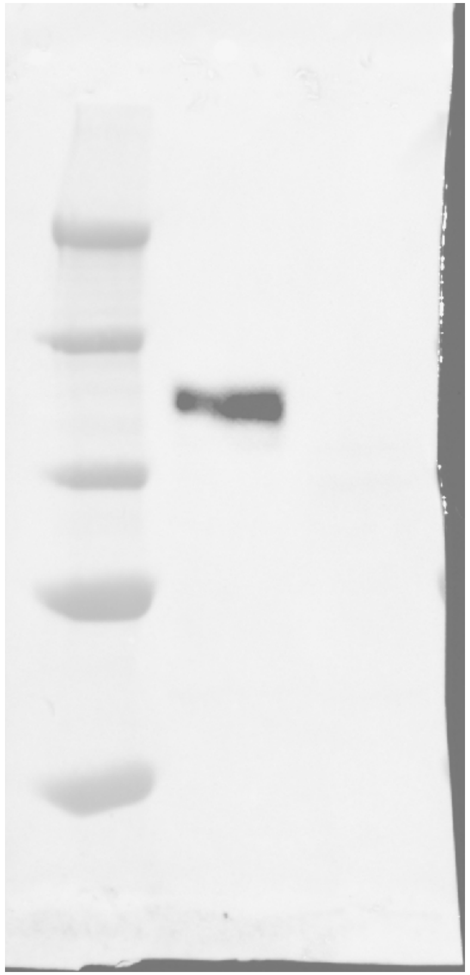

10/04/2024

Endoglina

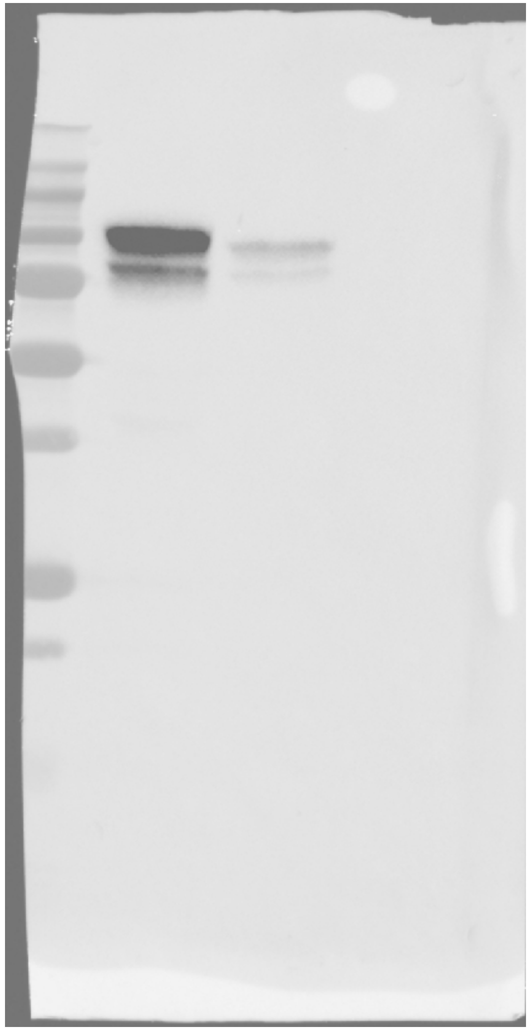

10/04/2024

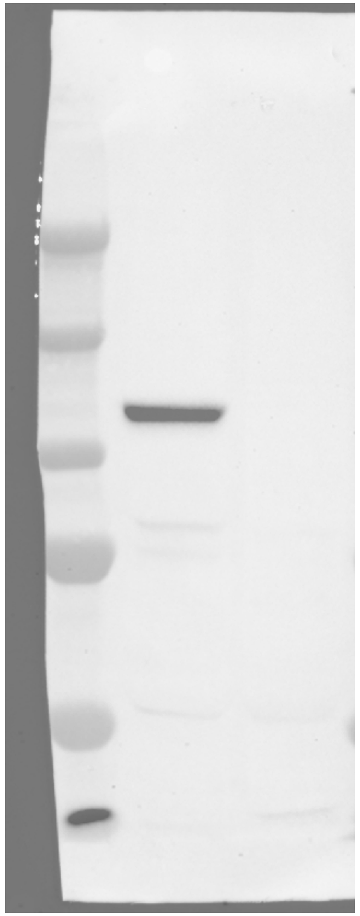

10/04/2024

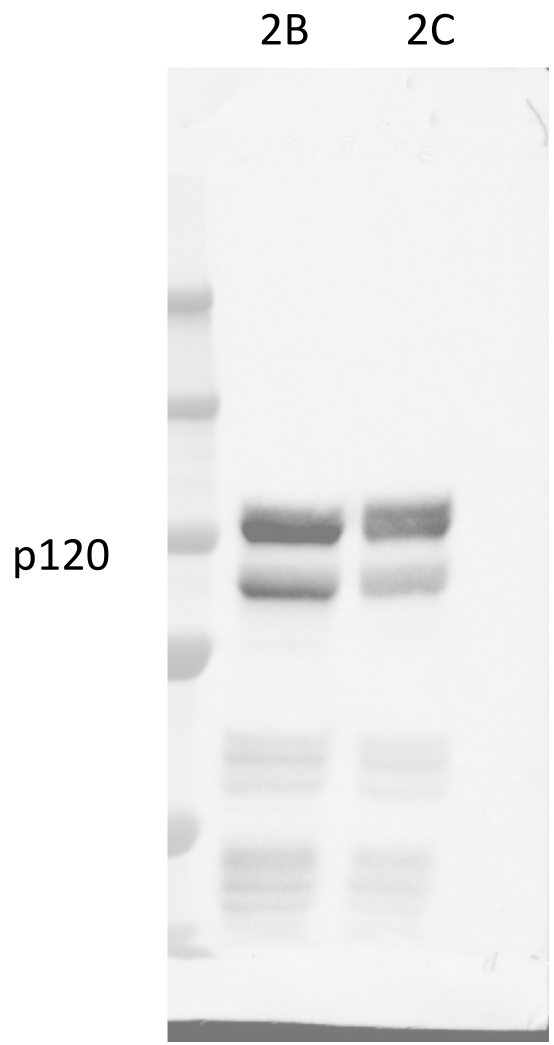

10/04/2024

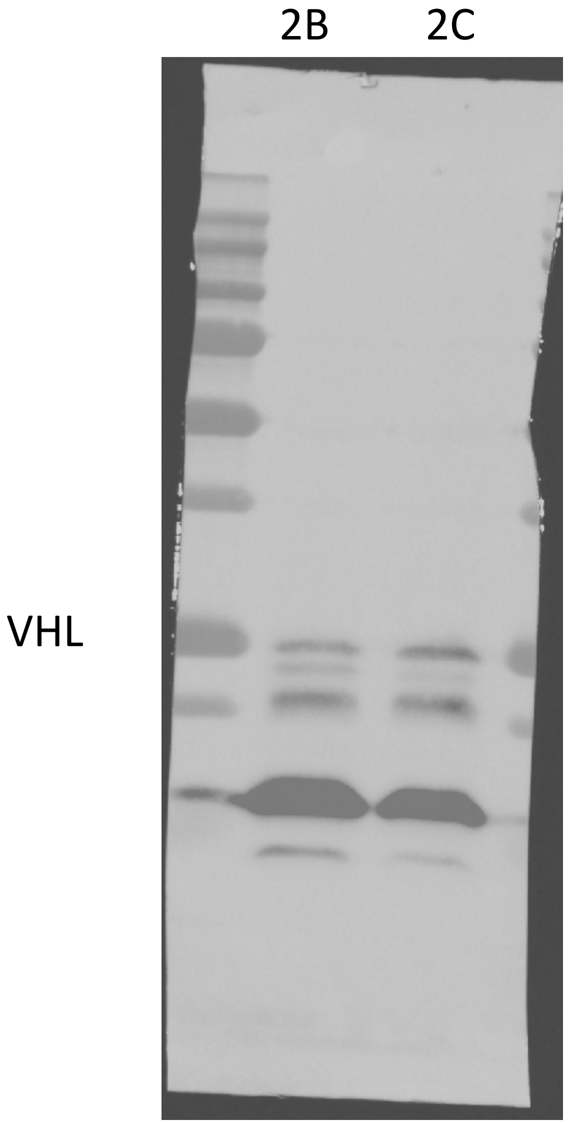

10/04/2024

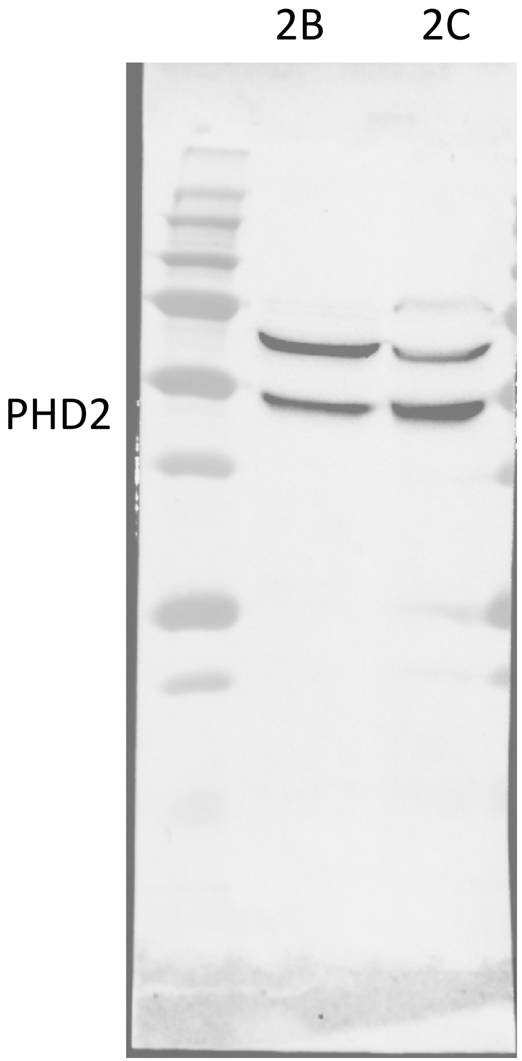

11/04/2024

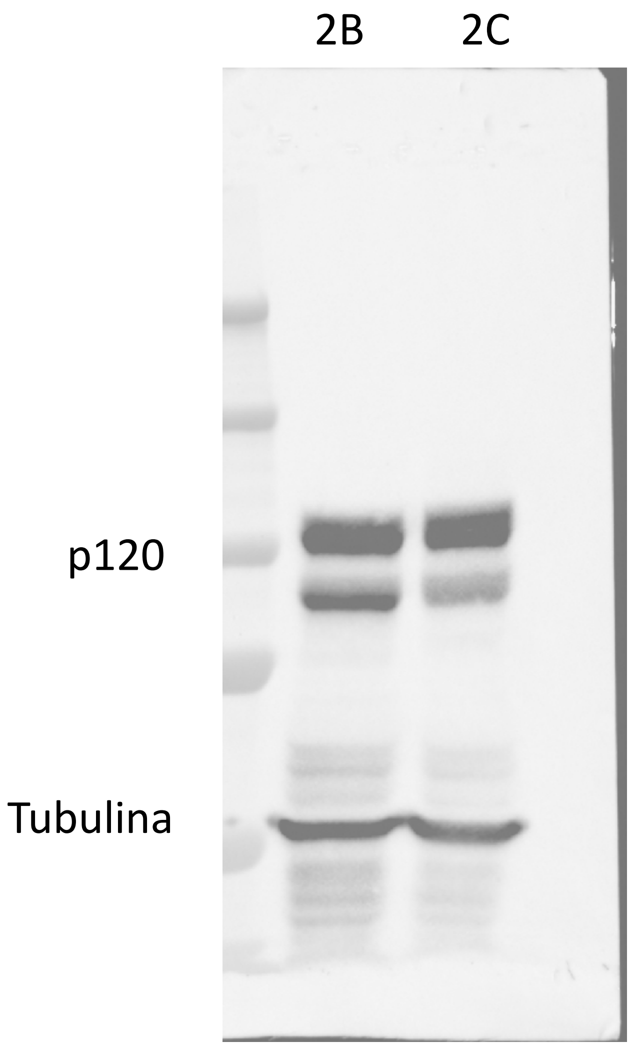

11/04/2024

2B 2C

TIE-1

Endoglina

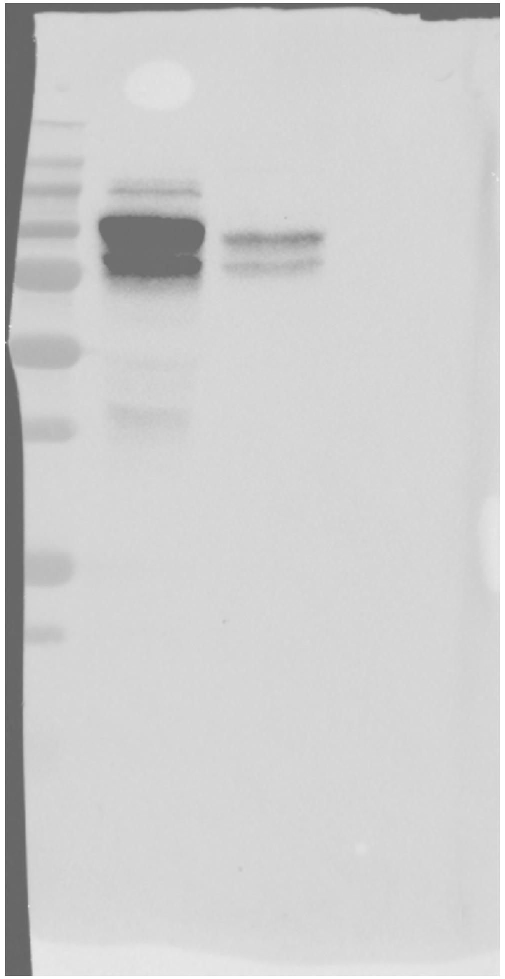

11/04/2024

2B 2C

Y658 VEC

B-catenina

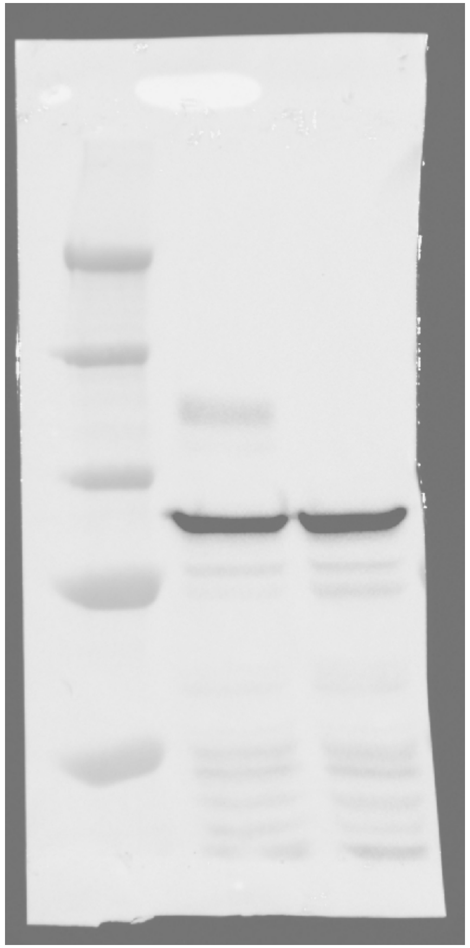

11/04/2024

MUM2B IP b-catenina

scb | siVHL | scb + CNO | siVHL + CNO

CE NE CE NE CE NE CE NE IgG NE

VEC Total

B-catenina

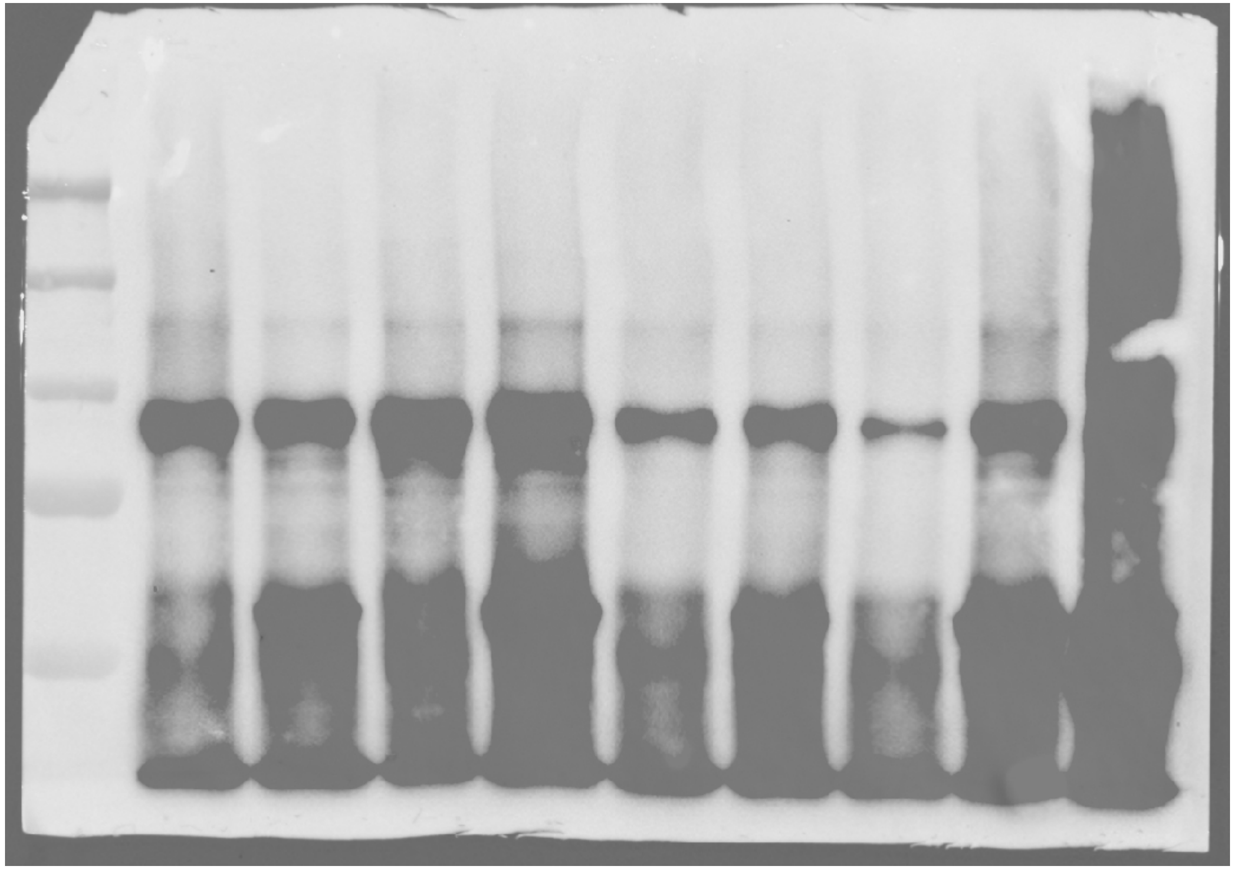

12/04/2024

2B 2C

Y658 VEC

B-catenina

Tubulina

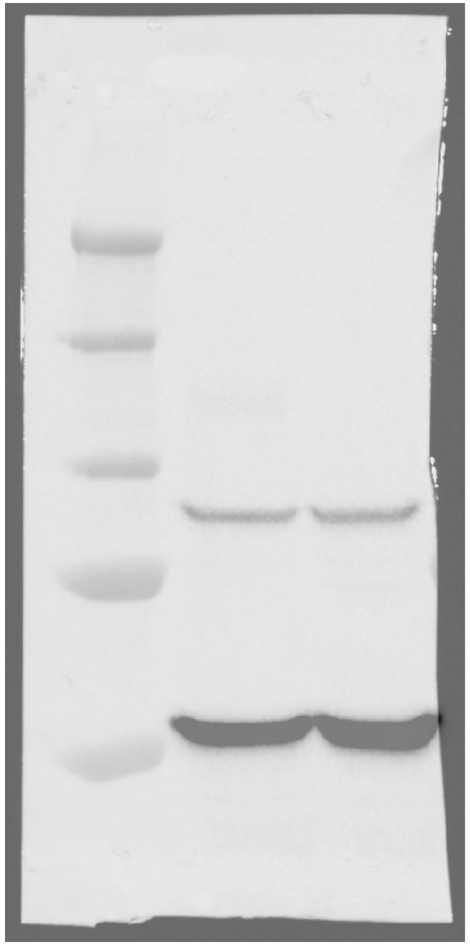

12/04/2024

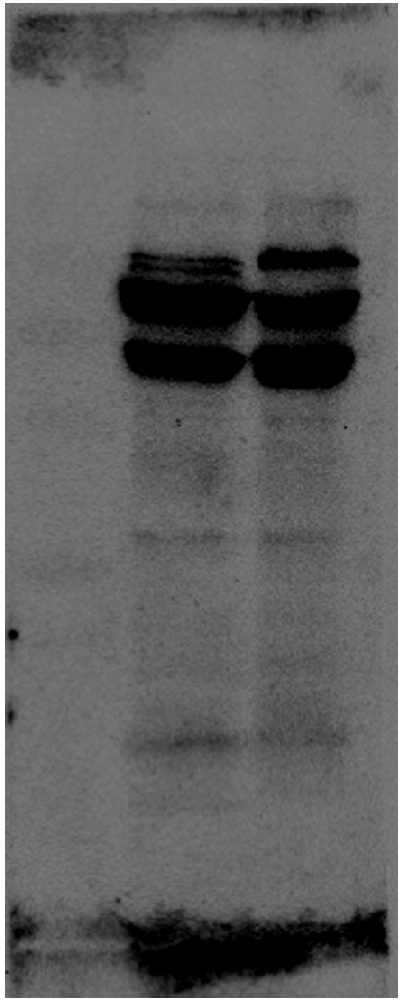

12/04/2024

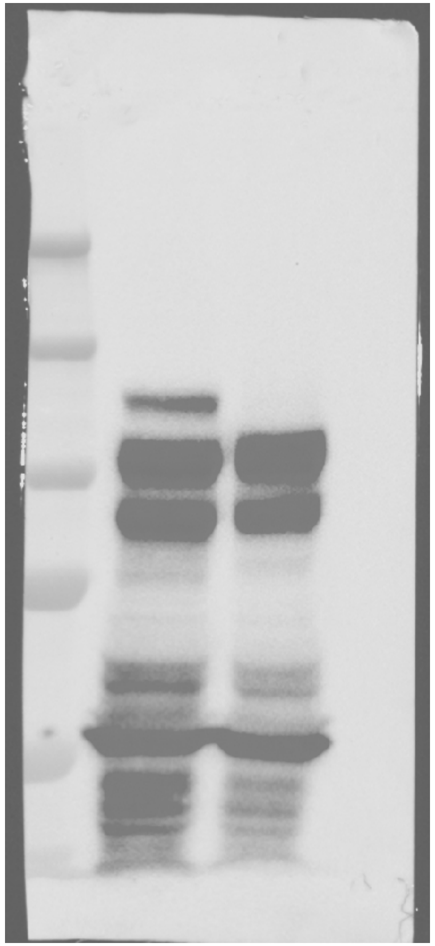

23/04/2024

2B 2C

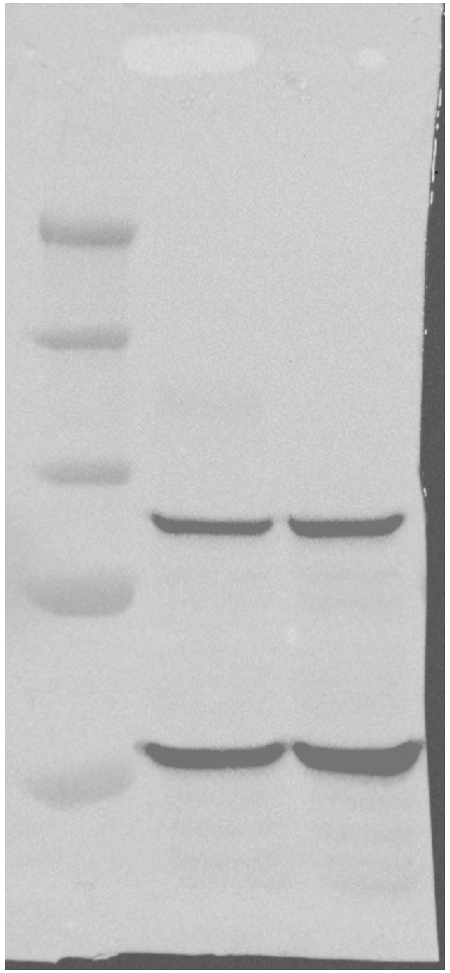

Y658 VEC

B-catenina

Tubulina

24/04/2024

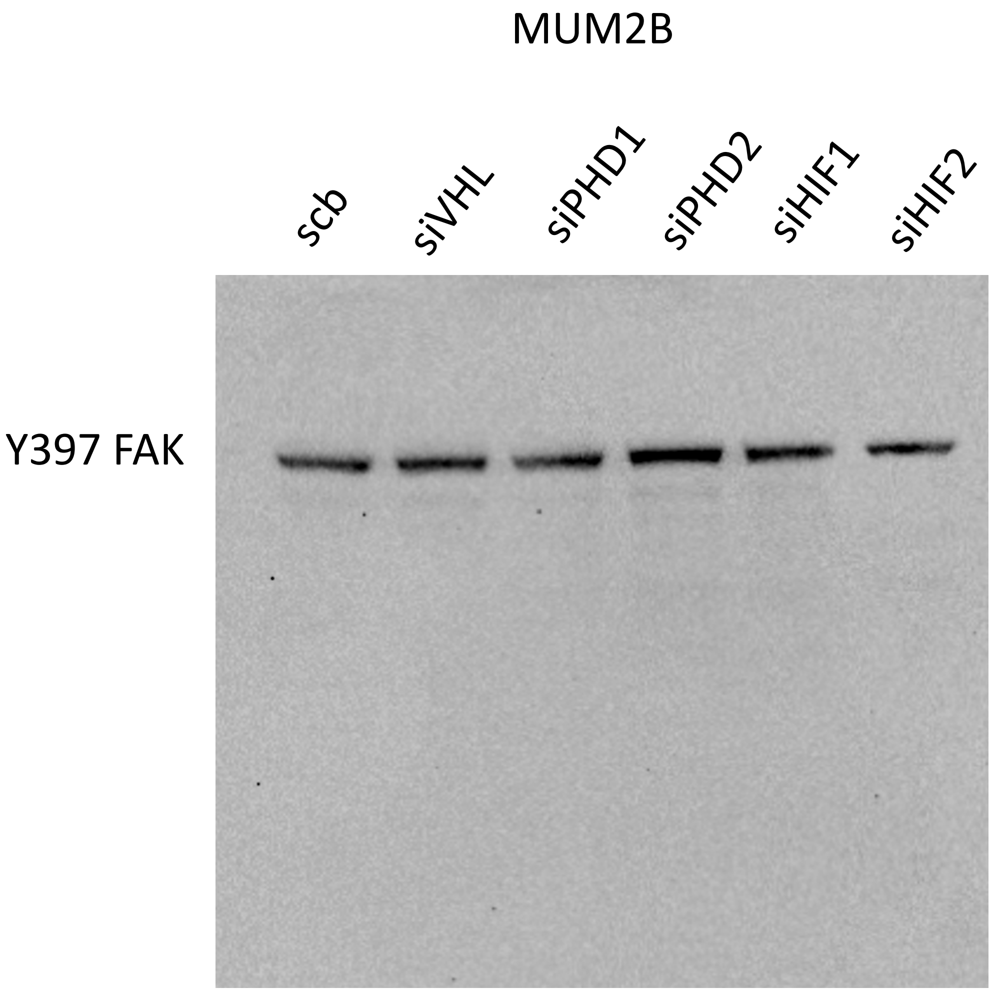

24/04/2024

MUM2C

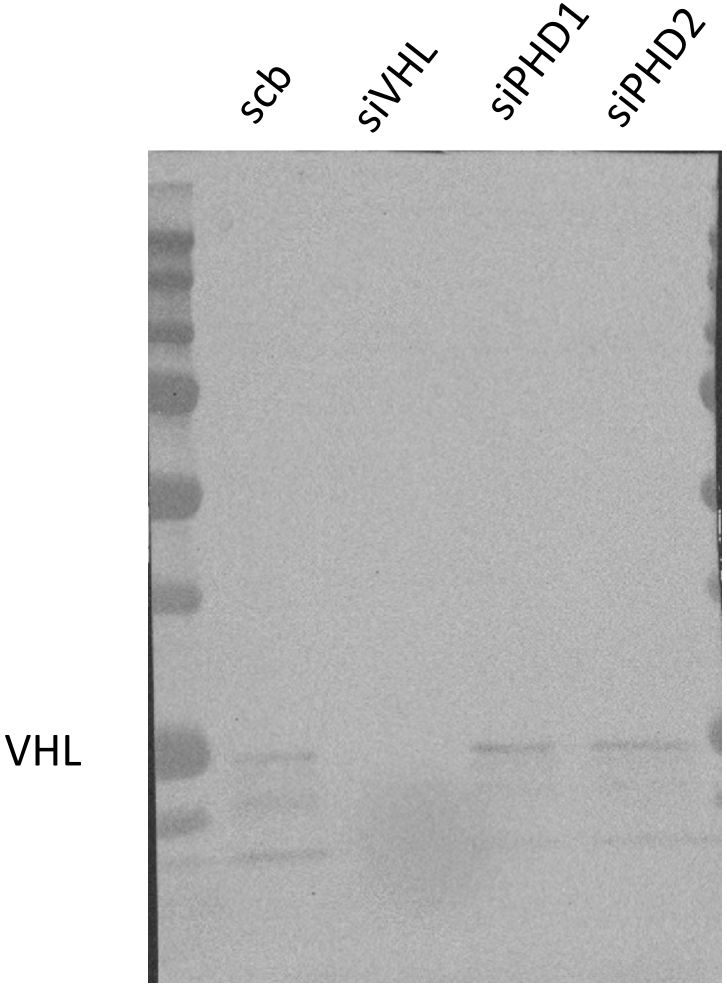

24/04/2024

MUM2C

Y397 FAK

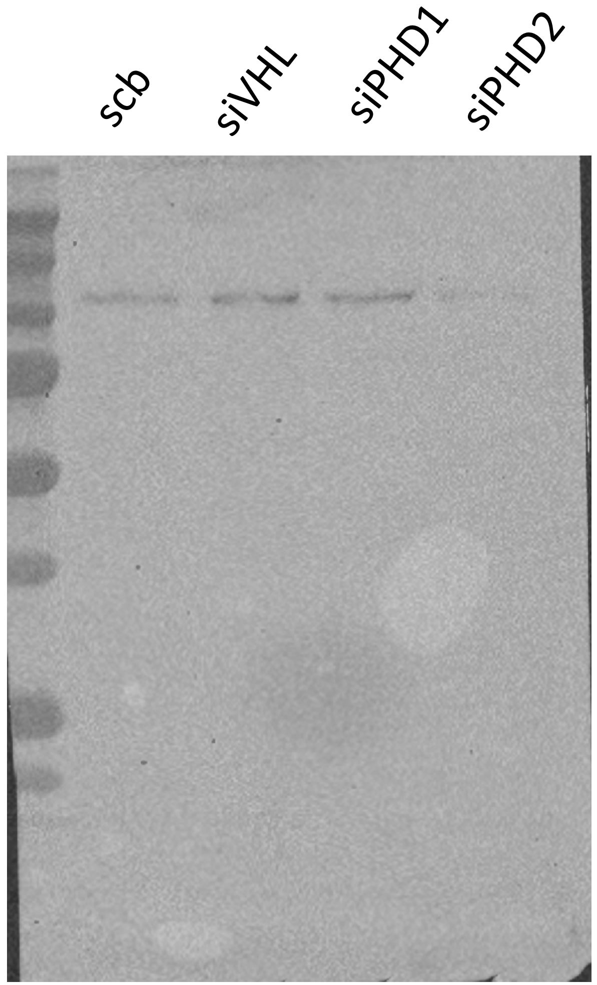

25/04/2024

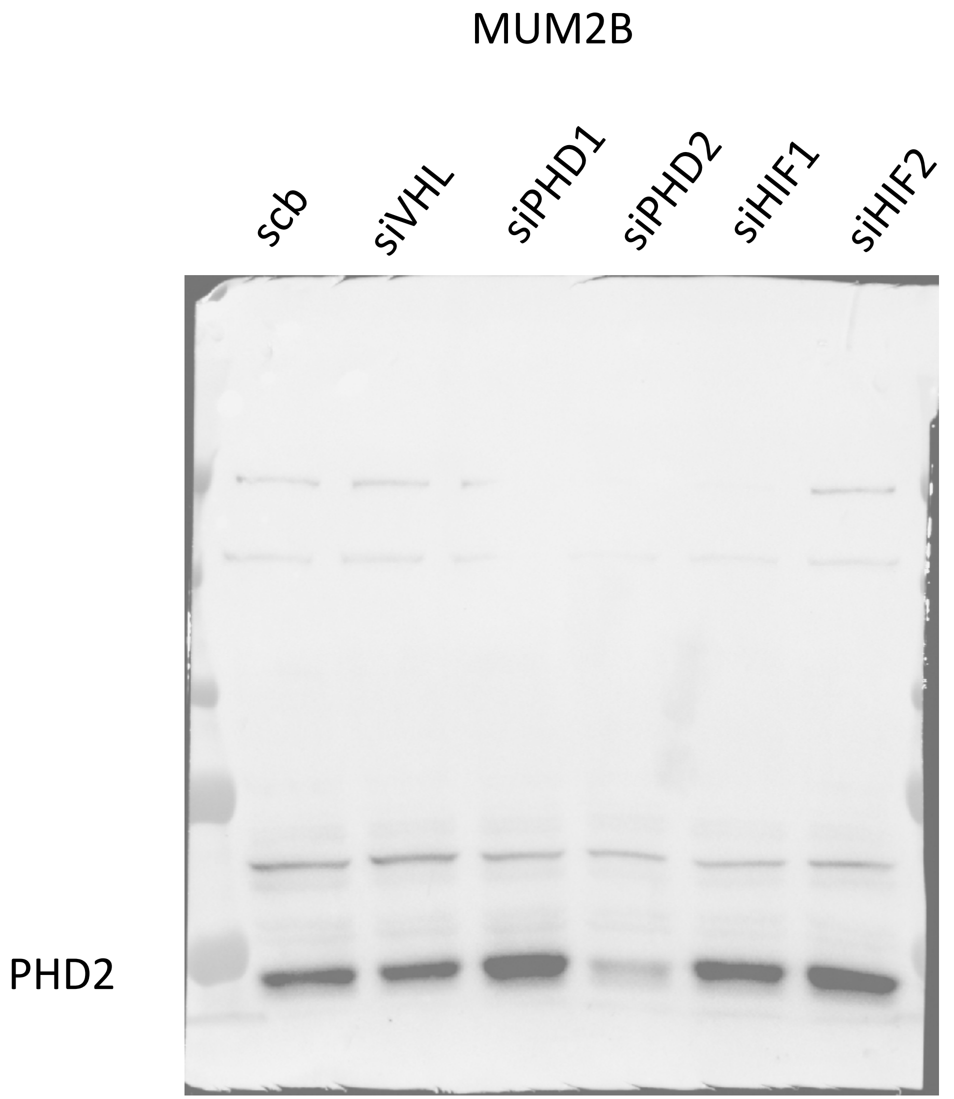

25/04/2024

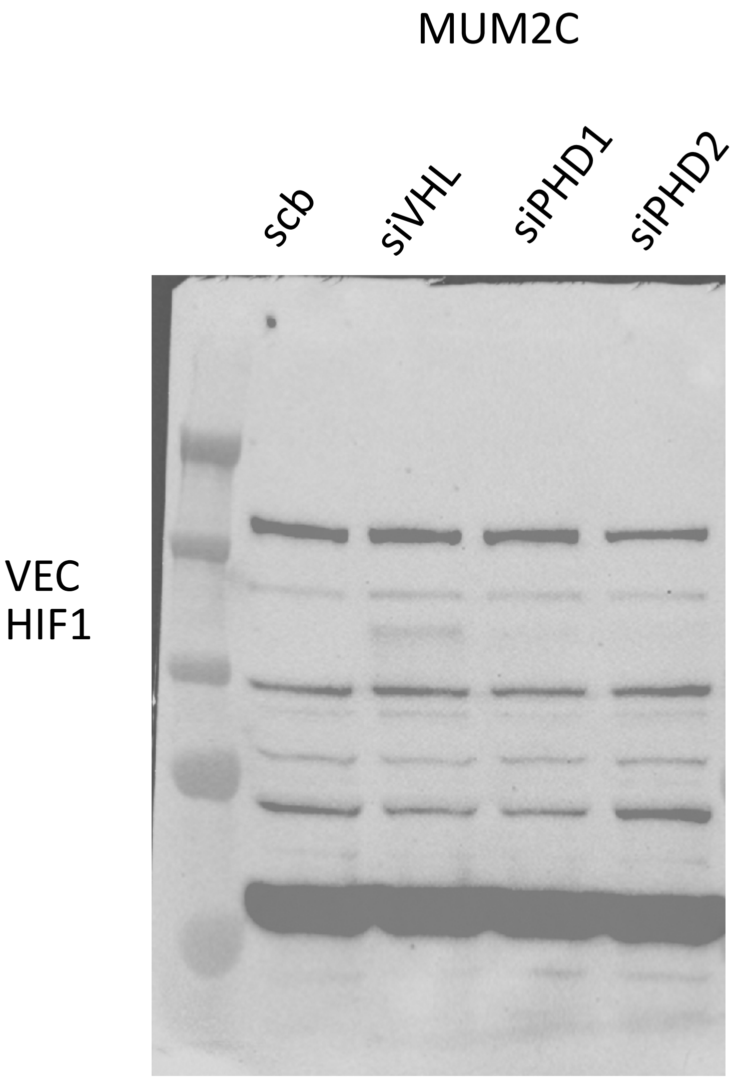

25/04/2024

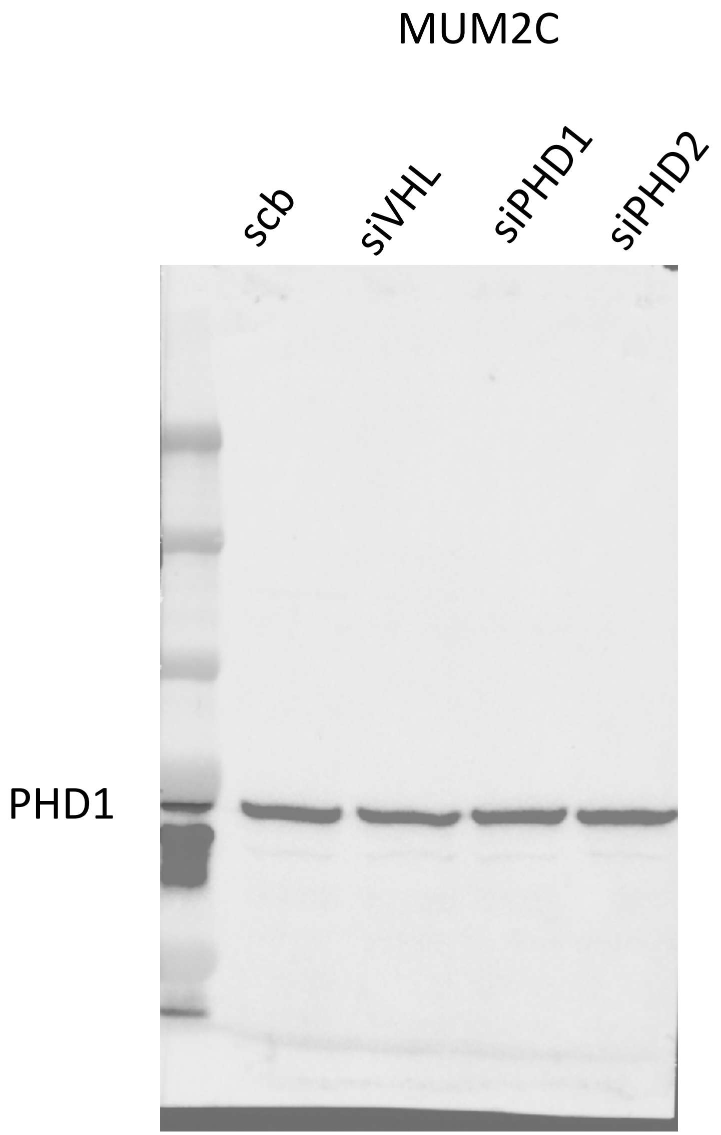

25/04/2024

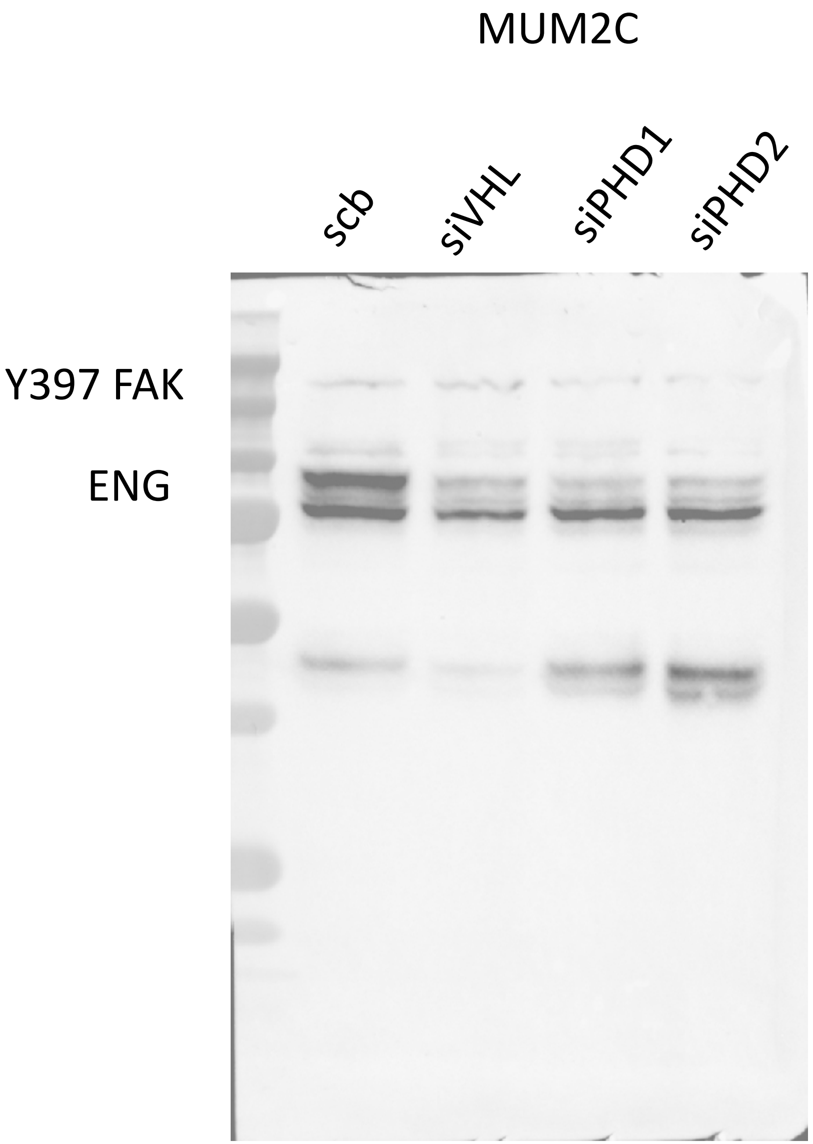

25/04/2024

MUM2C

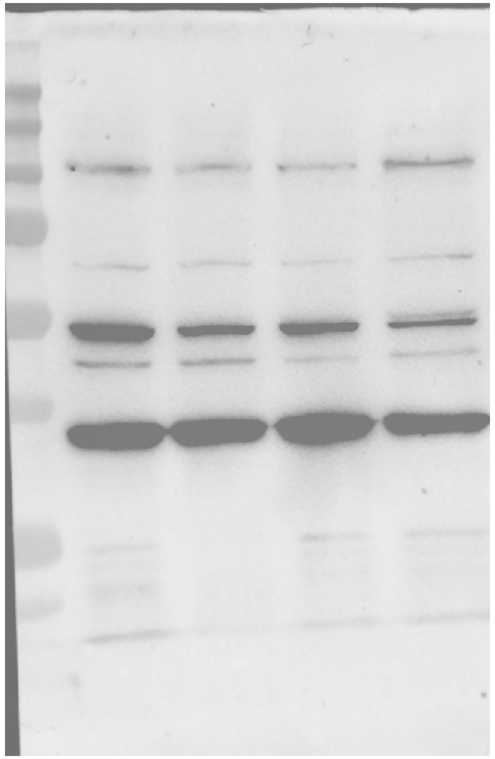

26/04/2024

MUM2B

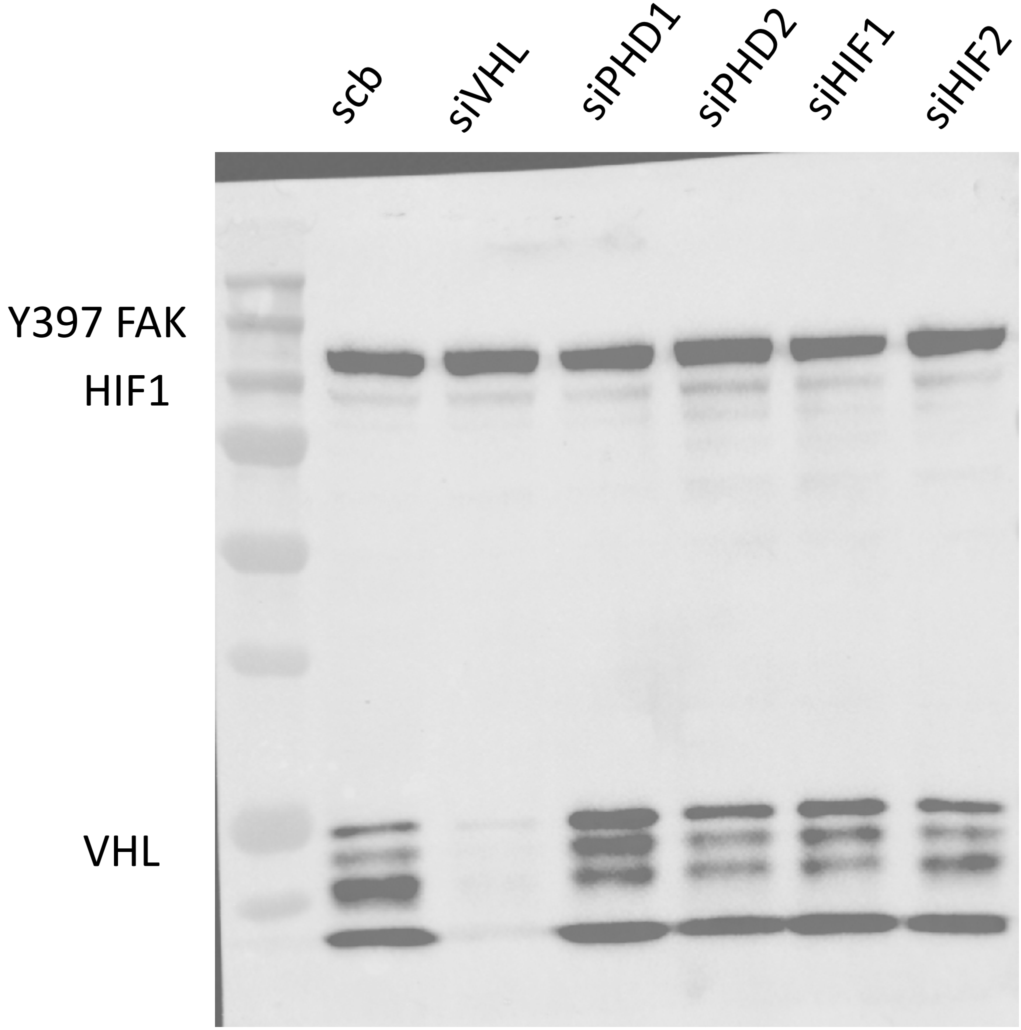

26/04/2024

MUM2B

scb siVHL siPHD1 siPHD2 siHIF1 siHIF2

VEC
HIF2

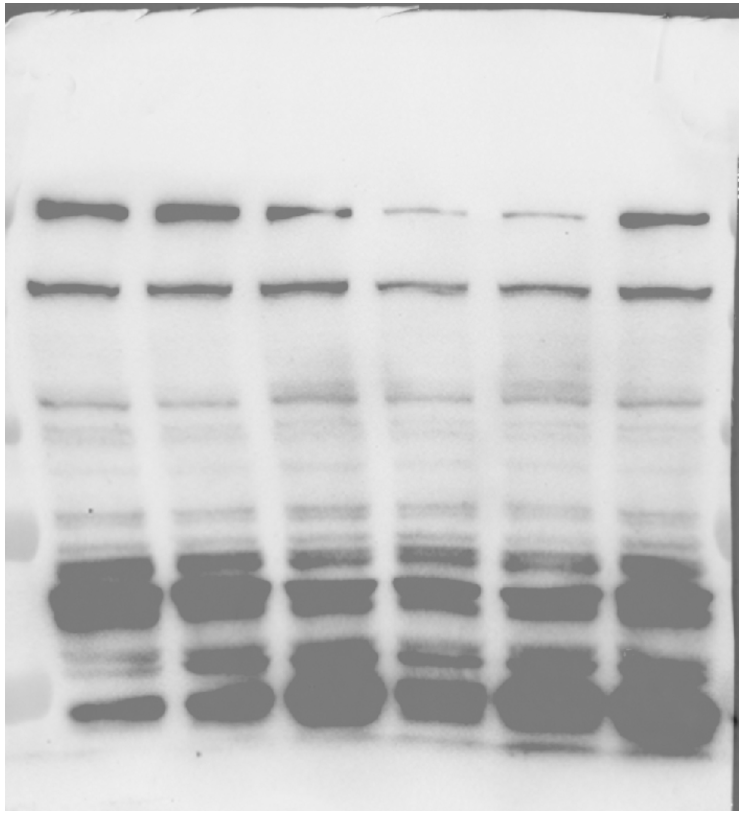

26/04/2024

MUM2C

scb siVHL siPHD1 siPHD2

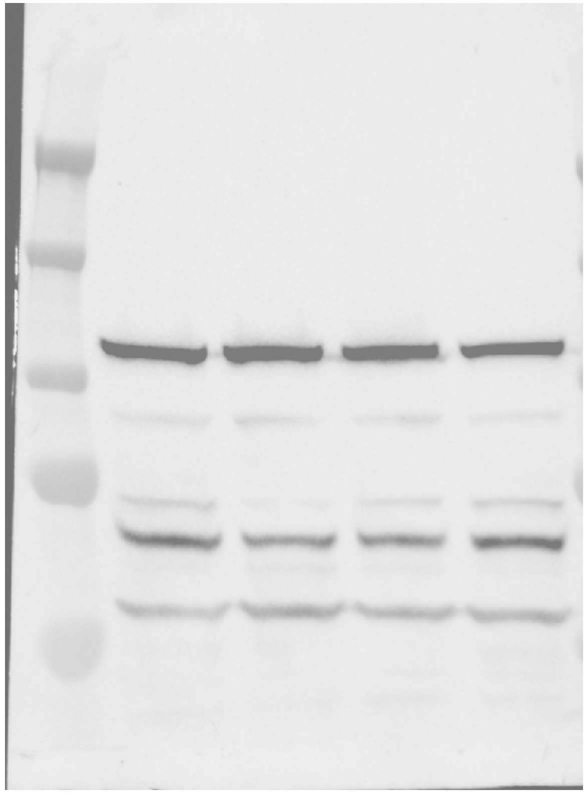

FAK

26/04/2024

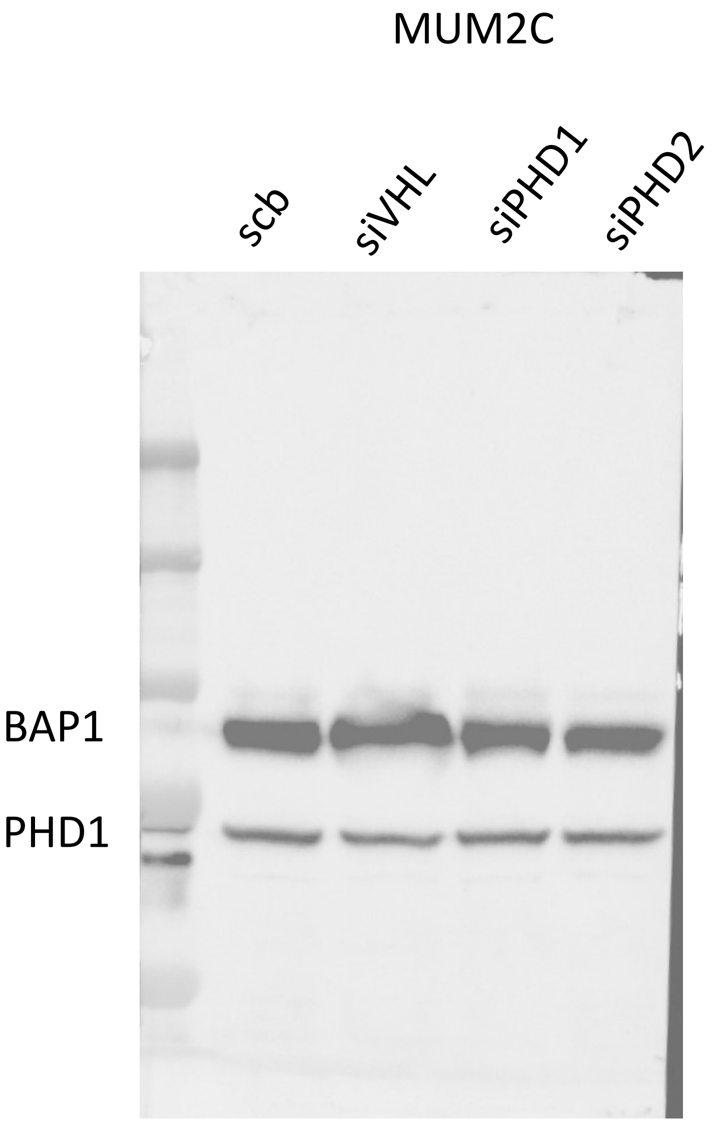

26/04/2024

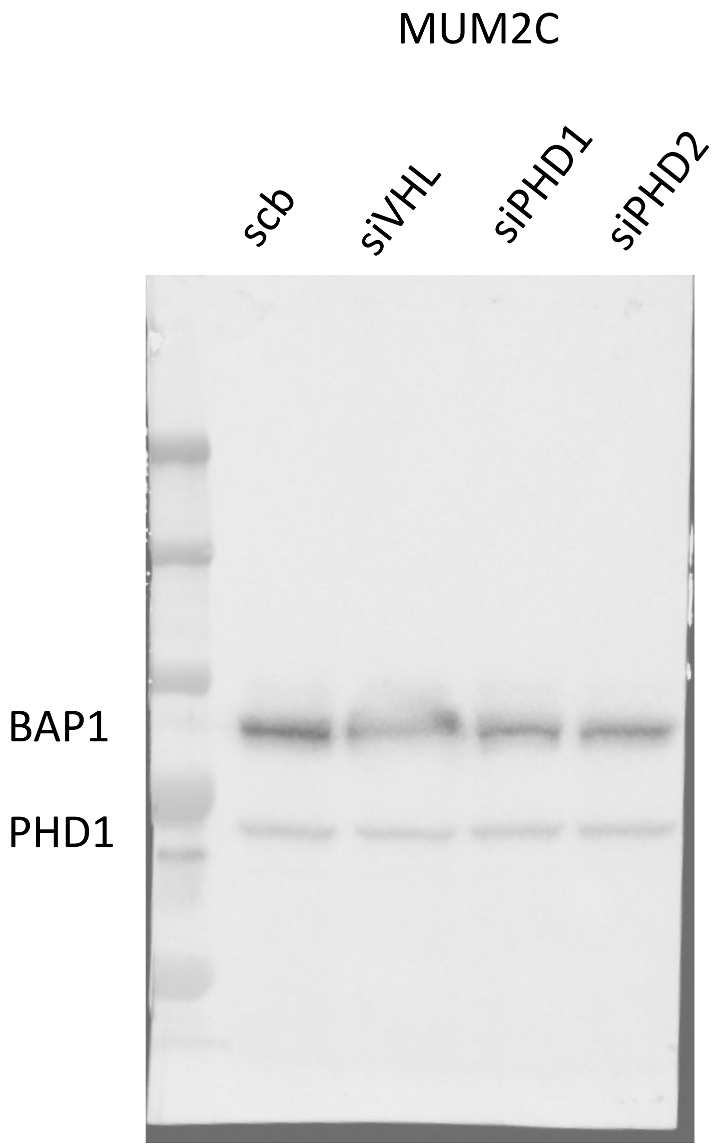

26/04/2024

MUM2C

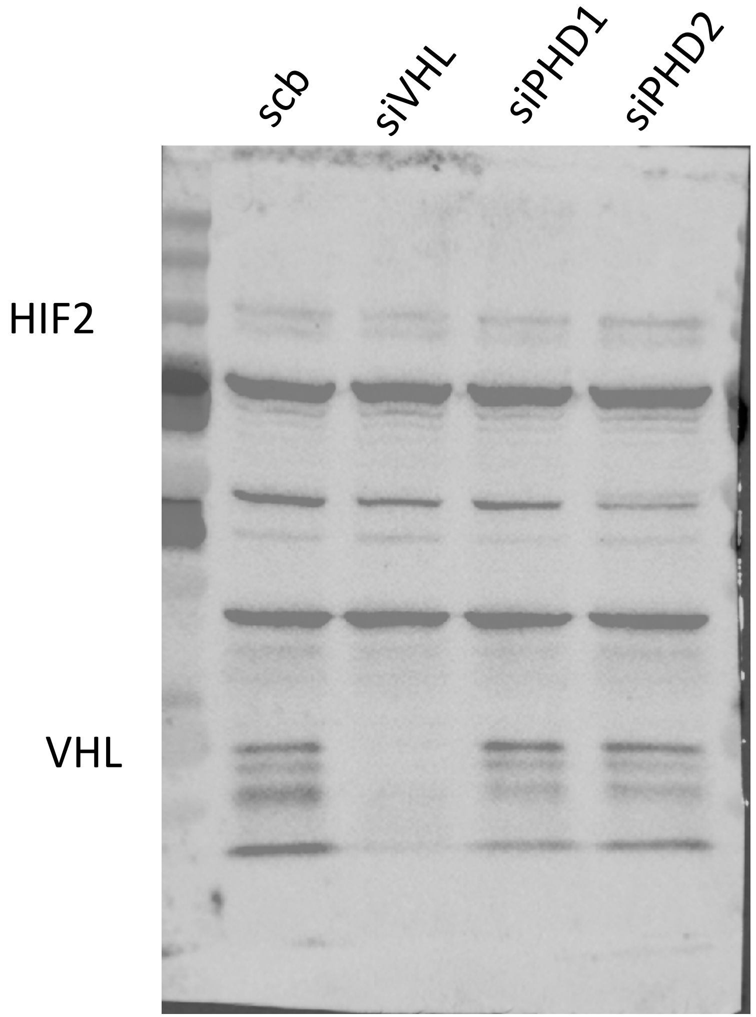

29/04/2024

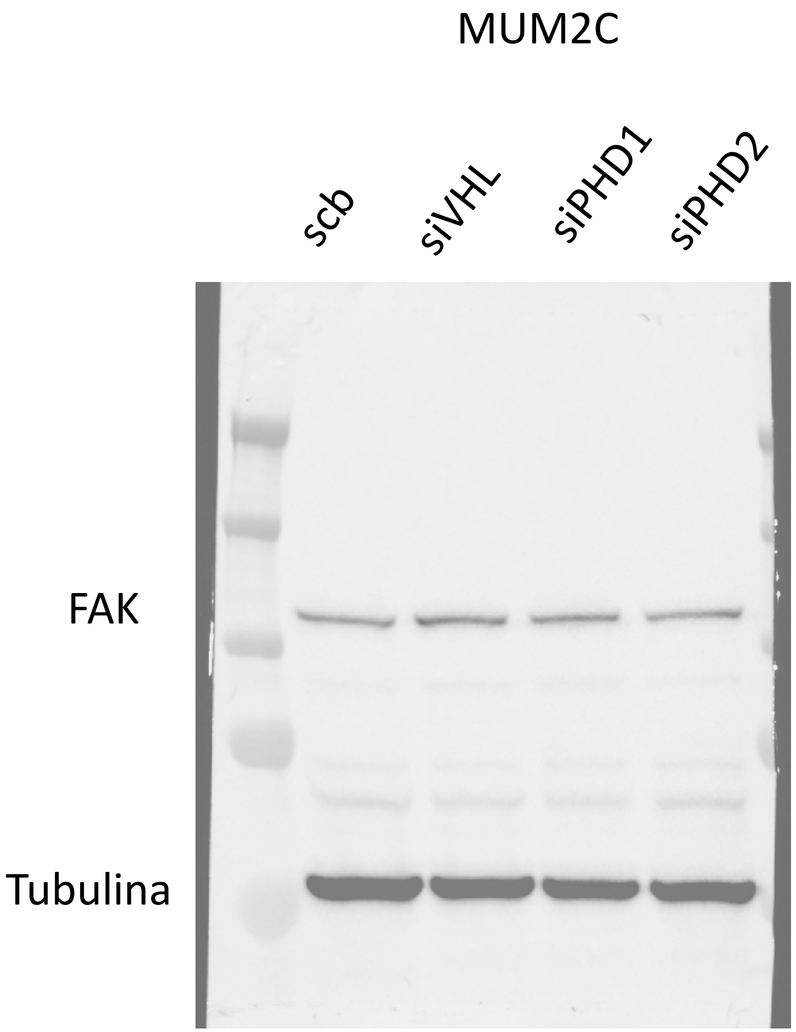

29/04/2024

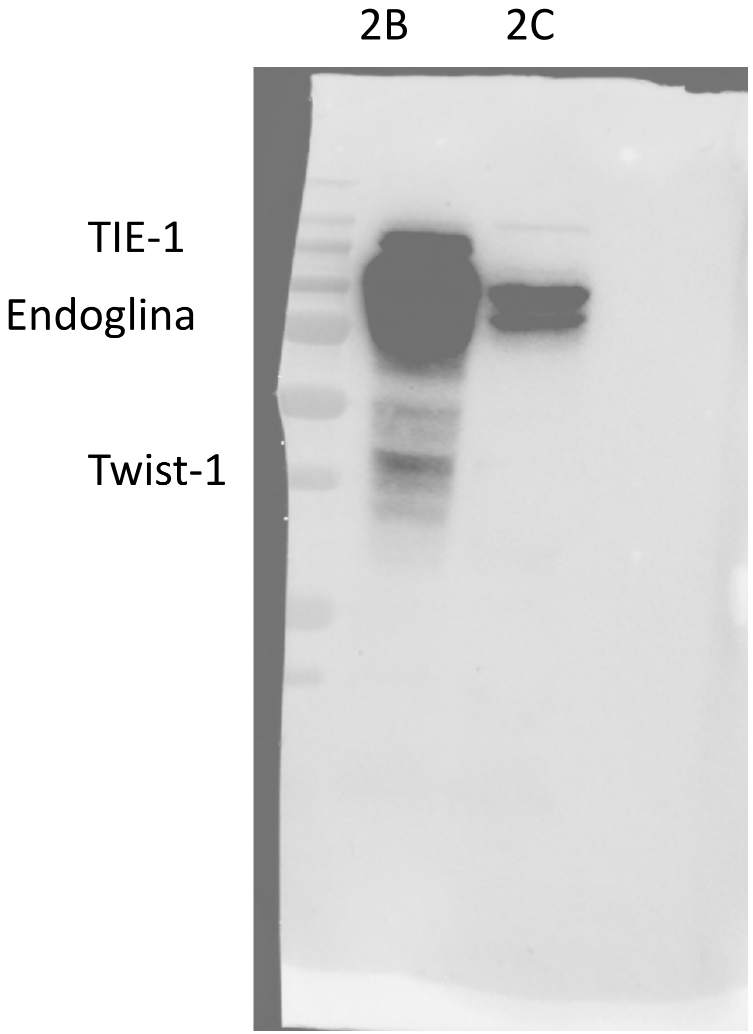

29/04/2024

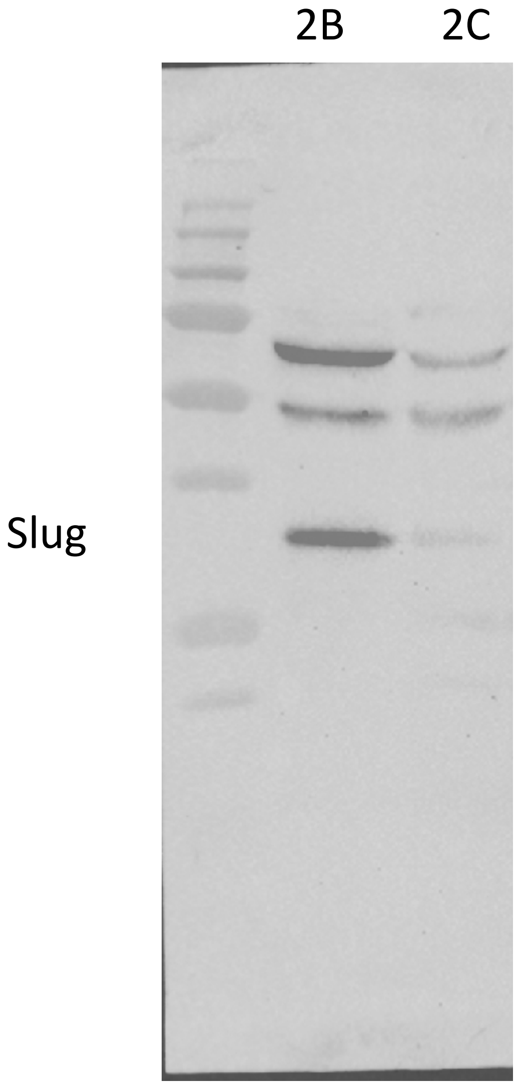

29/04/2024

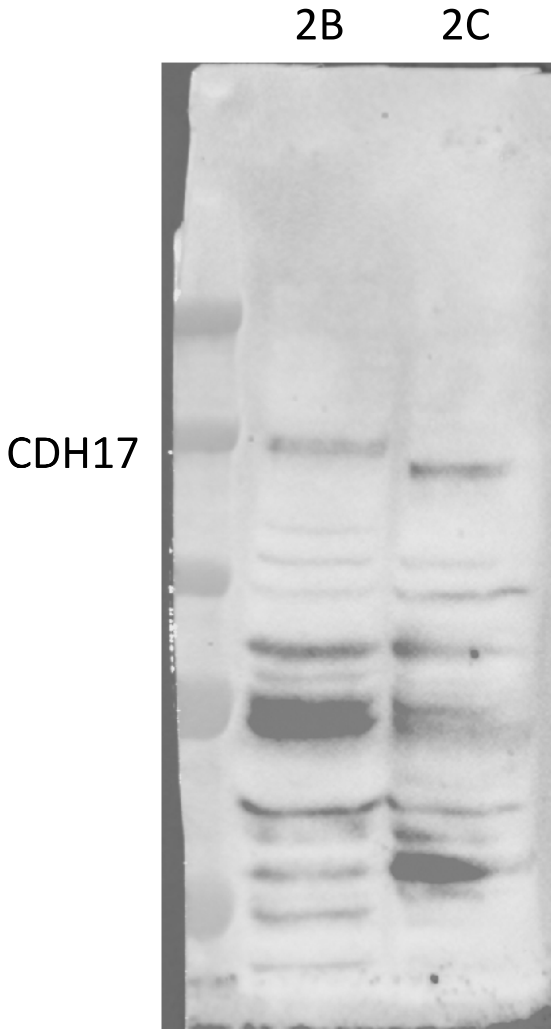

01/05/2024

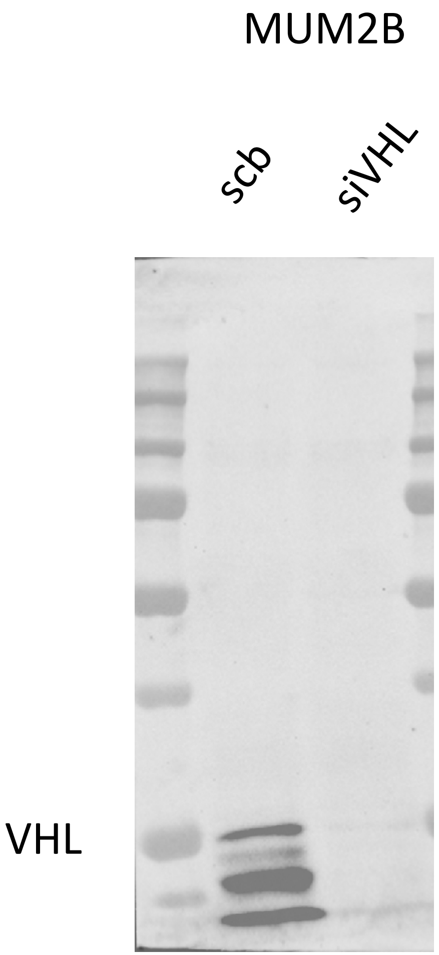

01/05/2024

MUM2B

scb siVHL

Y397 FAK

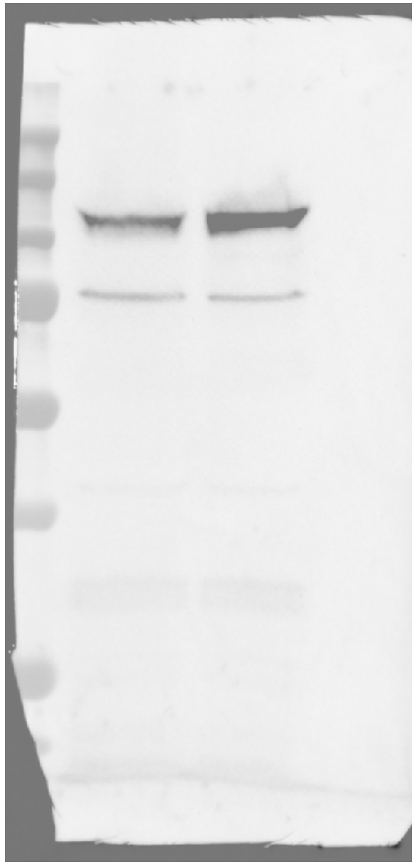

01/05/2024

MUM2B

scb siVHL

Y658 VEC

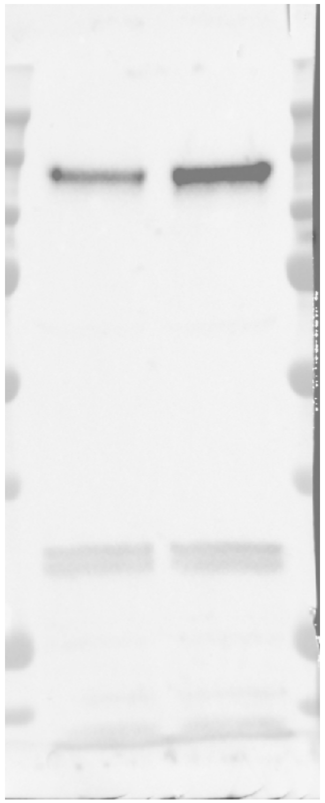

01/05/2024

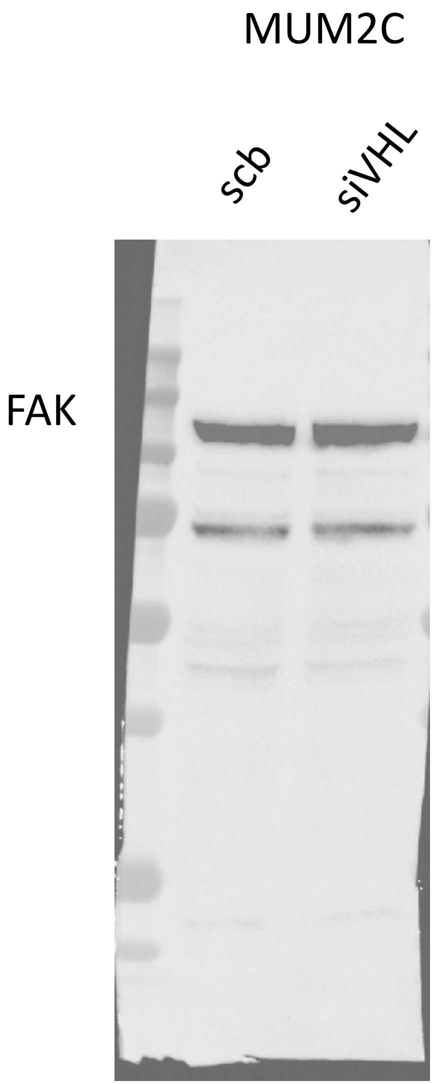

01/05/2024

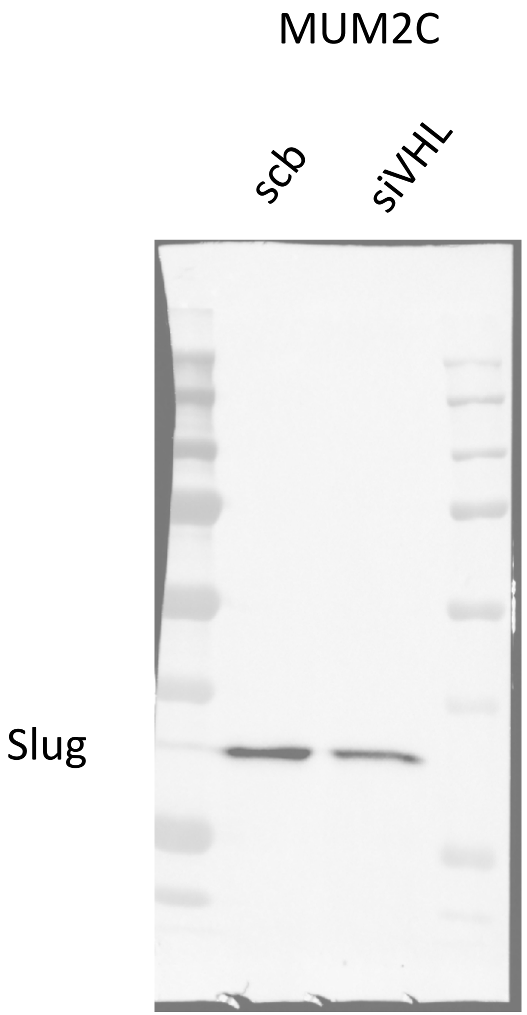

01/05/2024

MUM2C

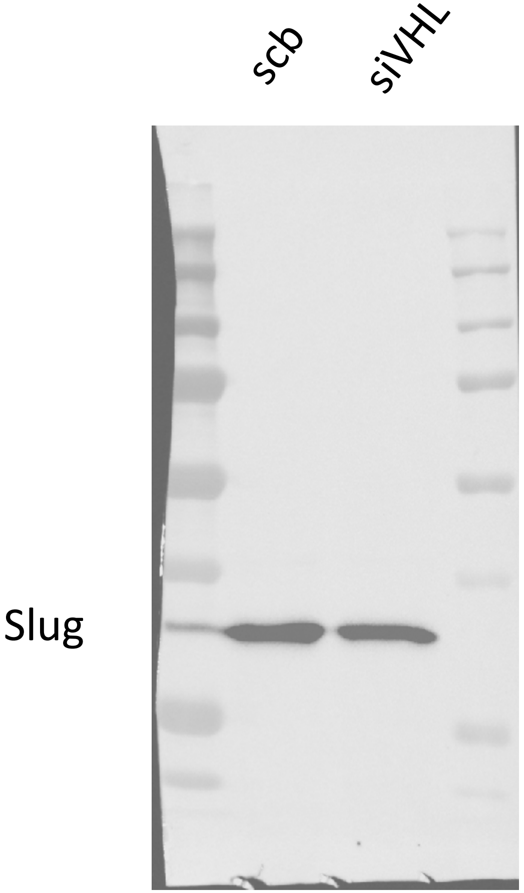

01/05/2024

MUM2B

Normoxia Hipoxia

Y397 FAK

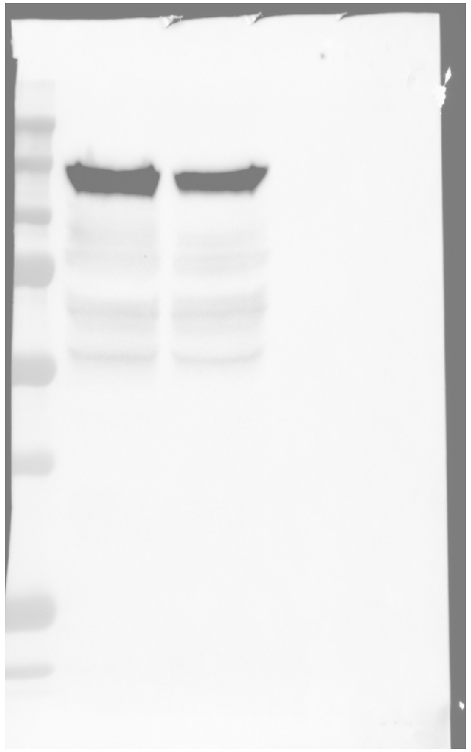

01/05/2024

MUM2B

Normoxia Hipoxia

Y658 VEC

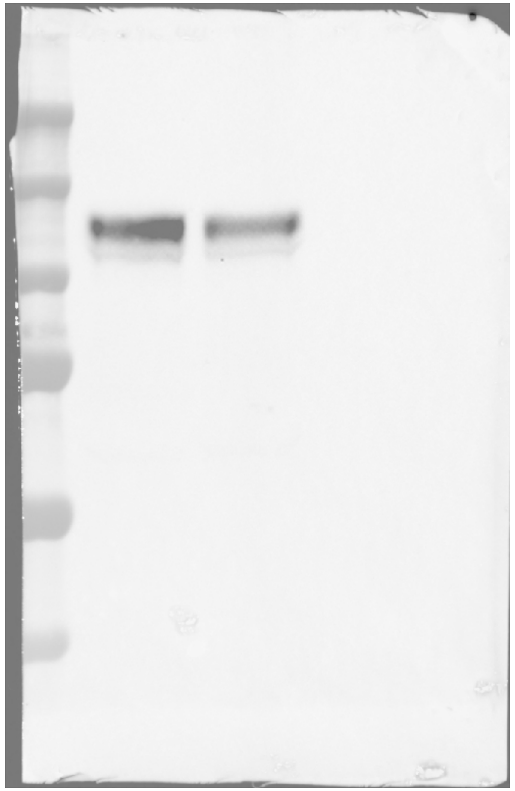

01/05/2024

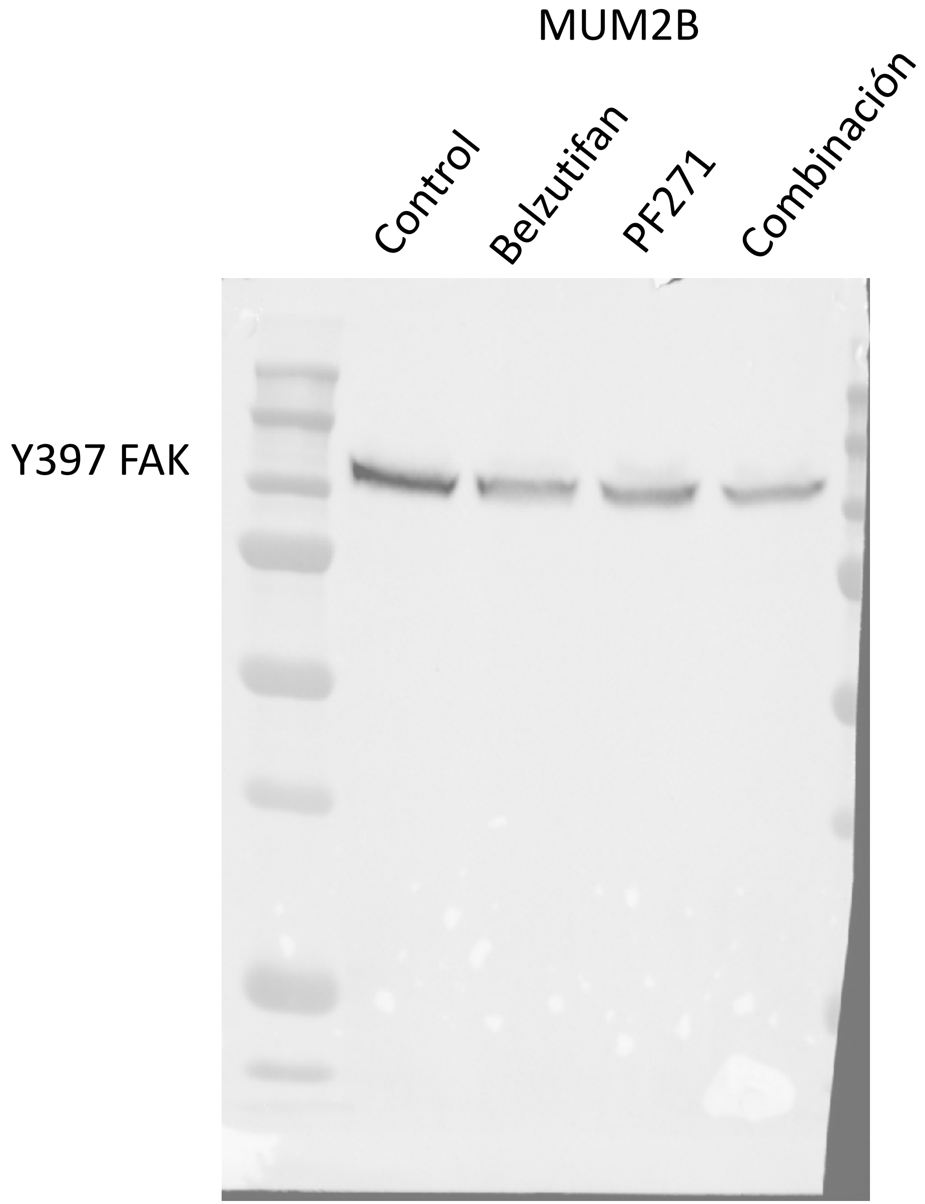

01/05/2024

MUM2B

Control | Belzutifan | PF271 | Belzu + PF271

Y658 VEC

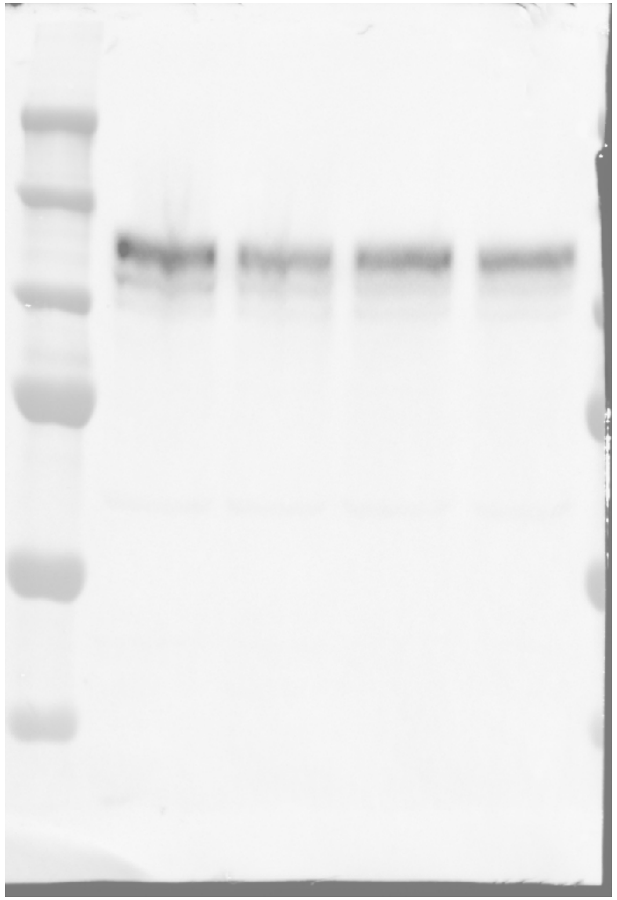

02/05/2024

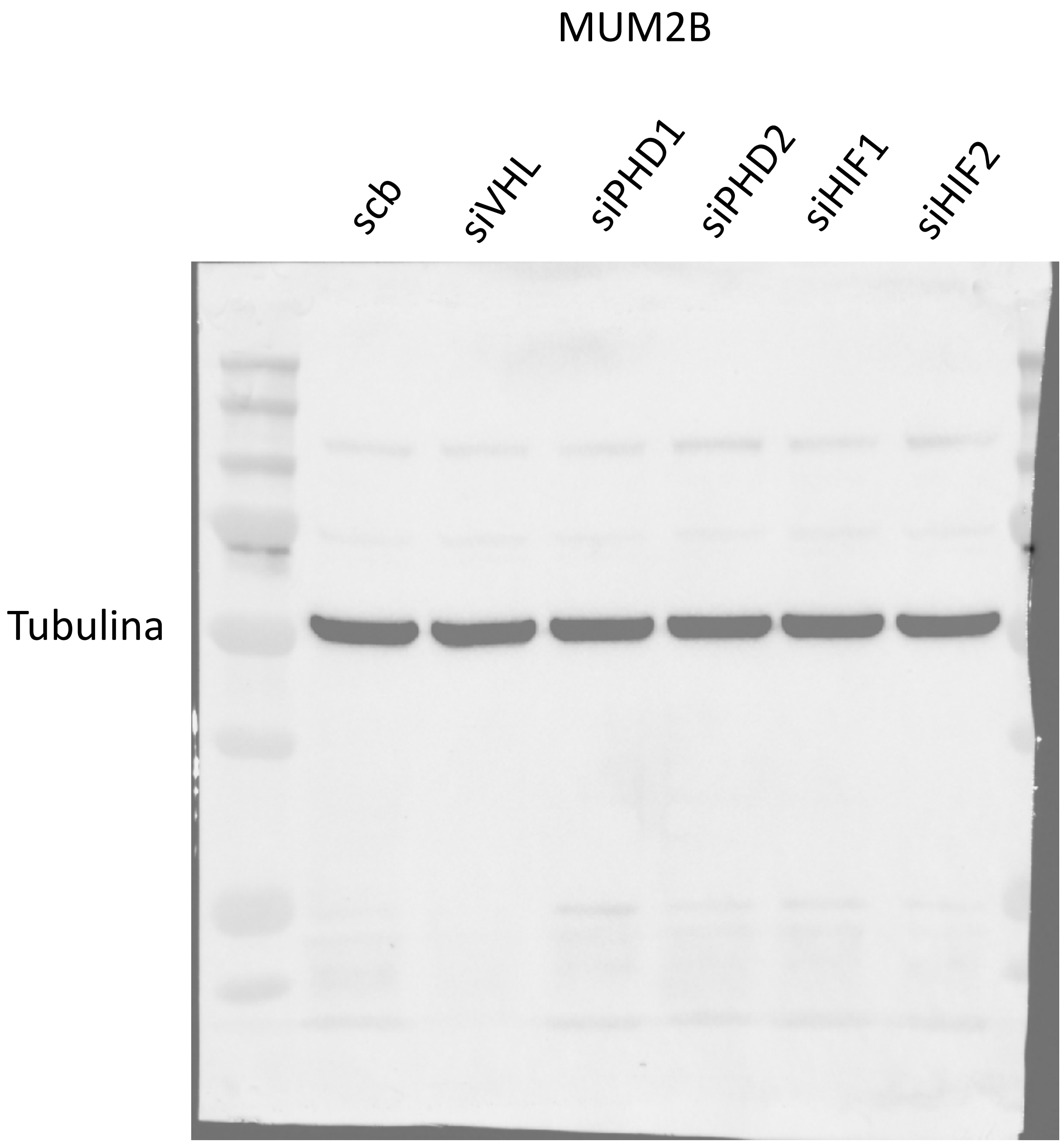

02/05/2024

MUM2B

scb siVHL

FAK

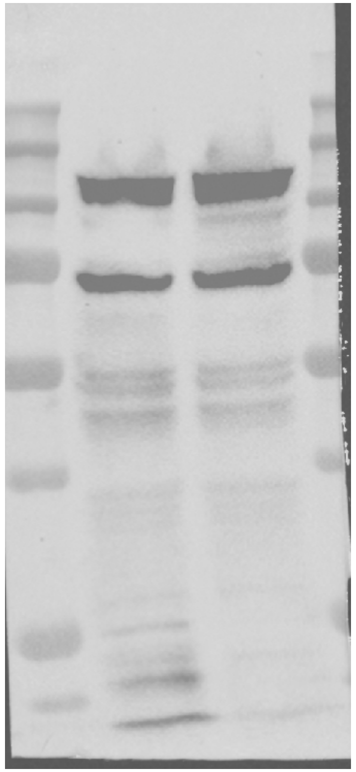

02/05/2024

MUM2B

scb siVHL

Y658
HIF1

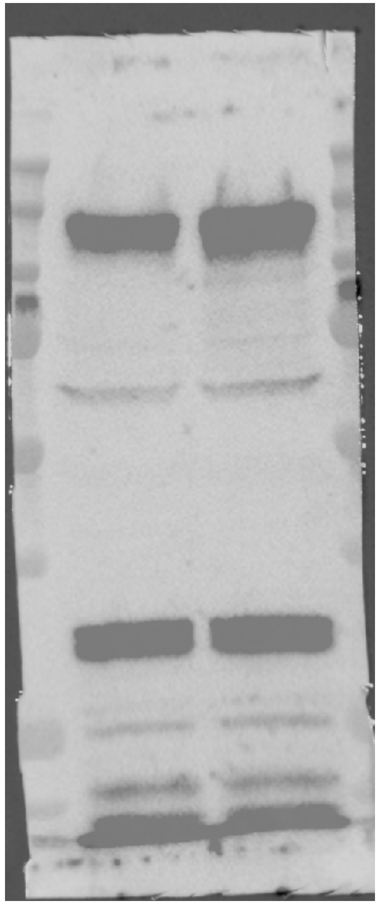

02/05/2024

MUM2B

scb siVHL

Y397
HIF2

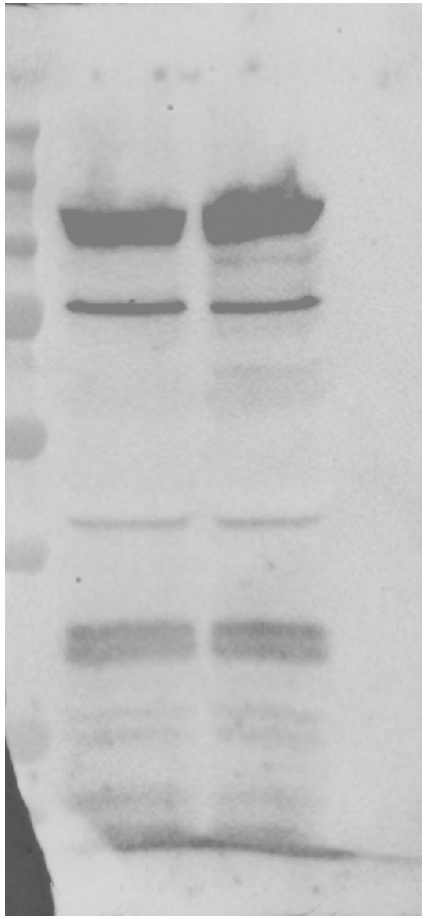

02/05/2024

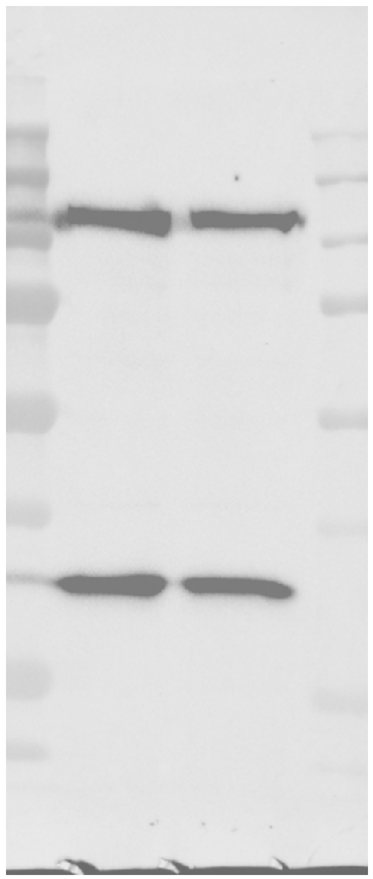

02/05/2024

MUM2C

scb siVHL

ENG

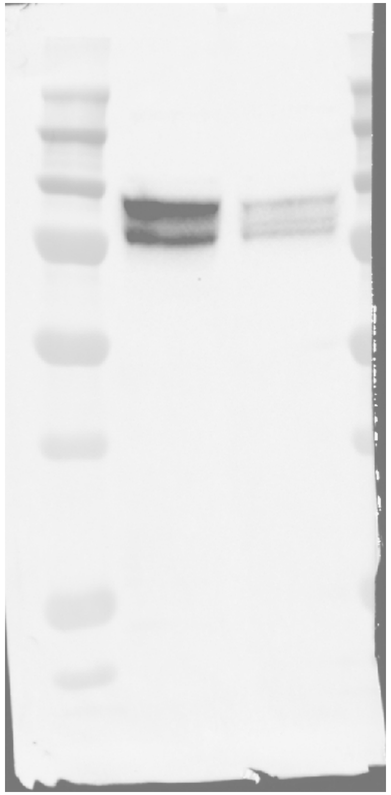

03/05/2024

MUM2B

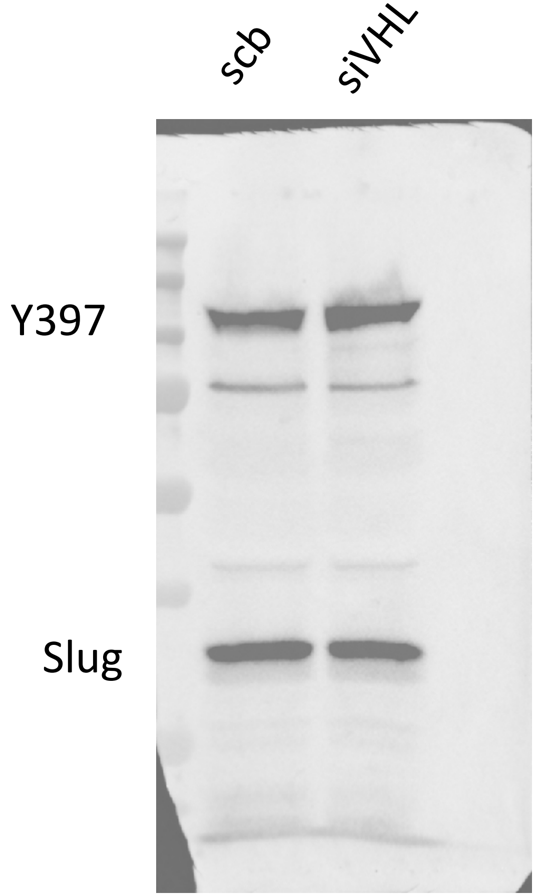

03/05/2024

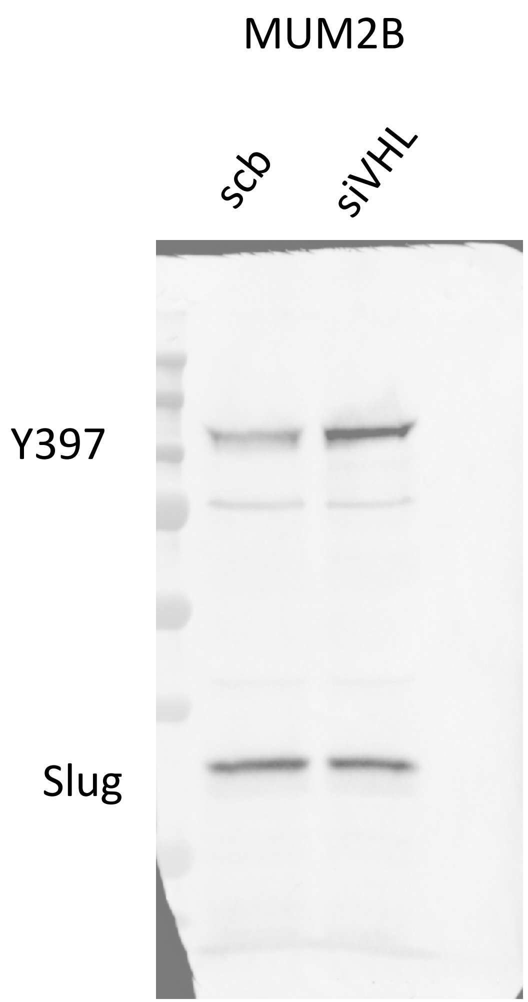

03/05/2024

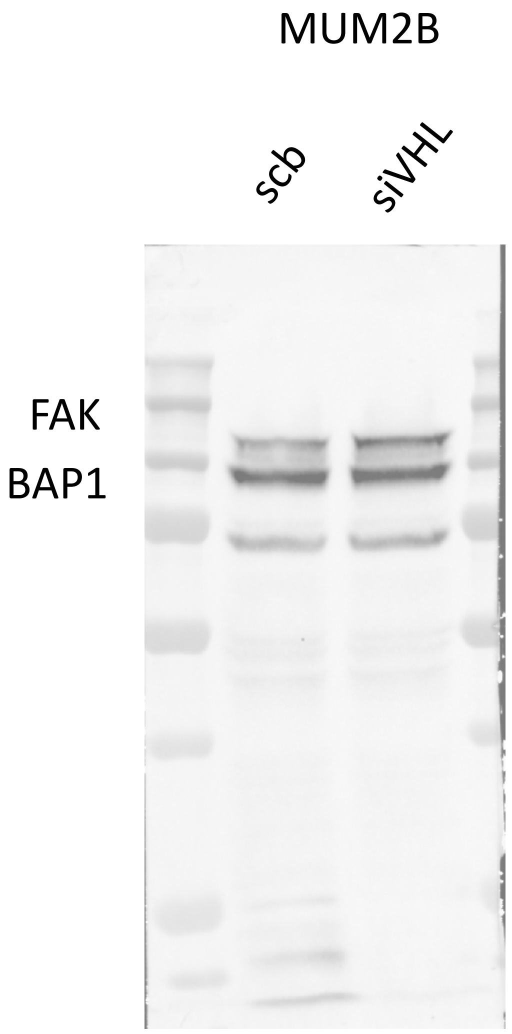

03/05/2024

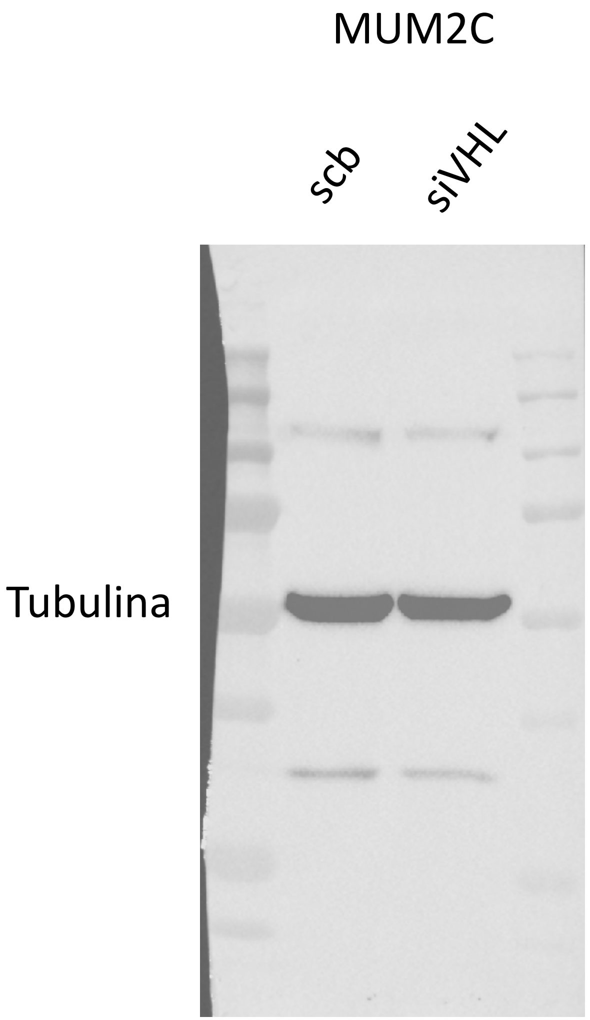

03/05/2024

MUM2B

Normoxia Hipoxia

Twist-1

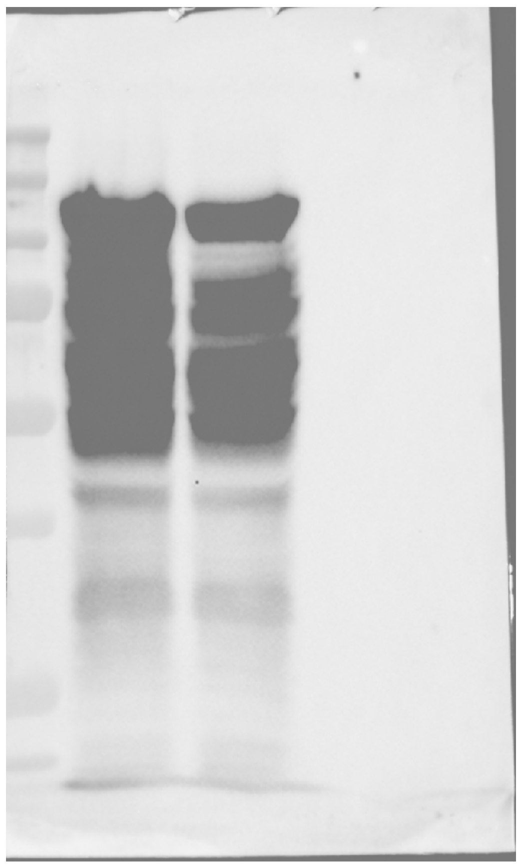

03/05/2024

MUM2B

Control Belzutifan PF271 Combinación

Y397 FAK

BAP1

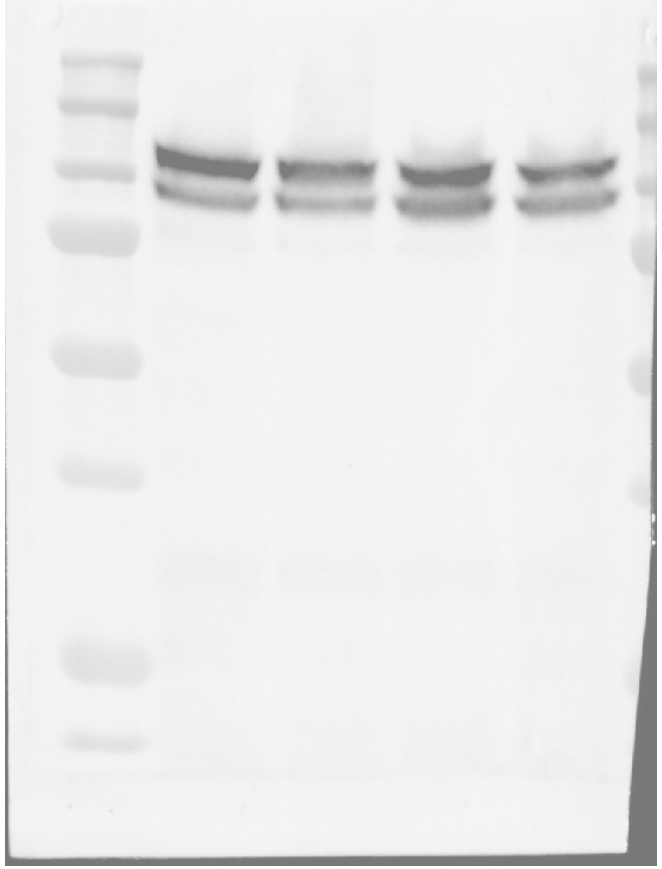

03/05/2024

MUM2B

Control
Belzutifan
PF271
Belzu + PF271

Y658 VEC

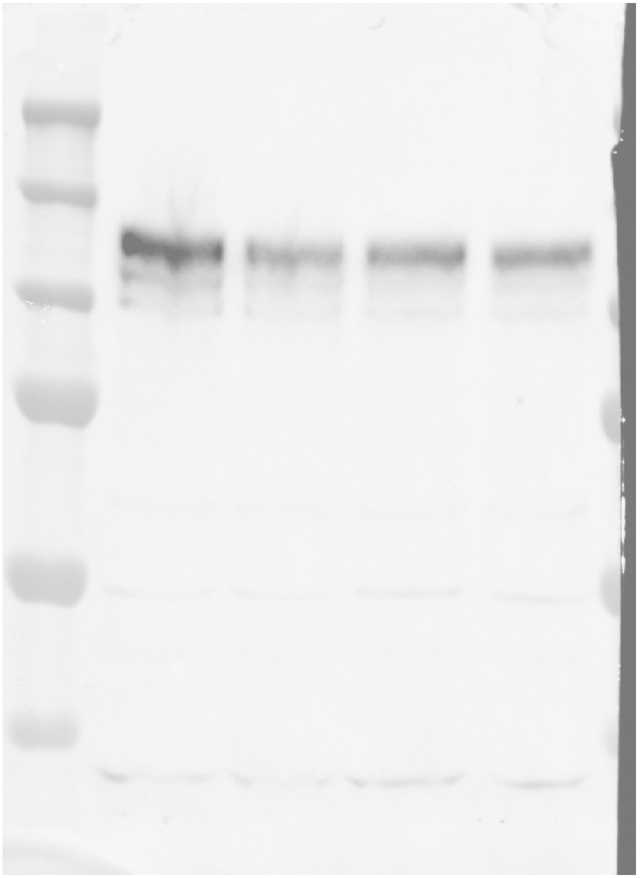

06/05/2024

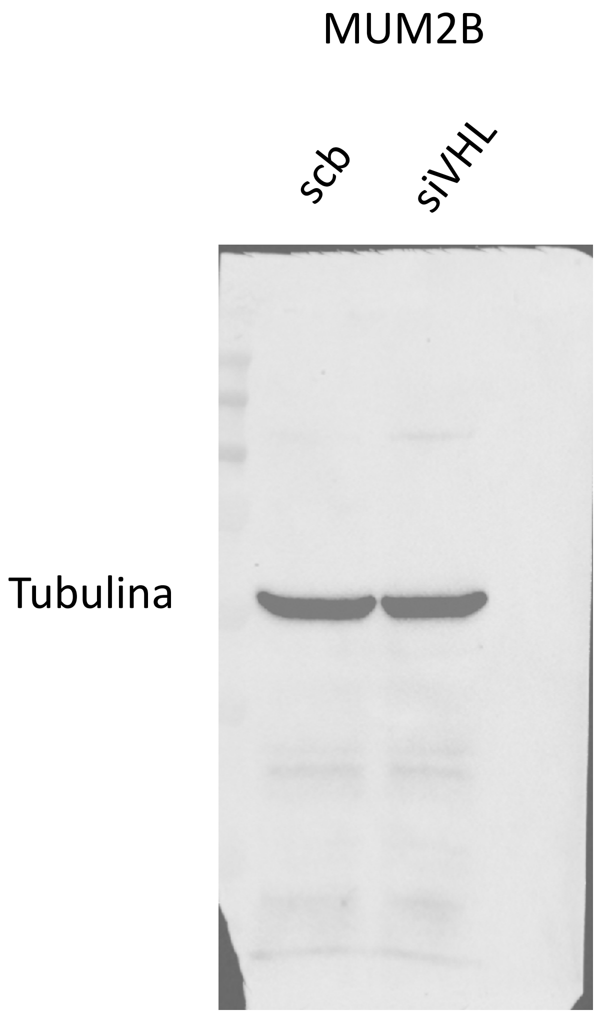

06/05/2024

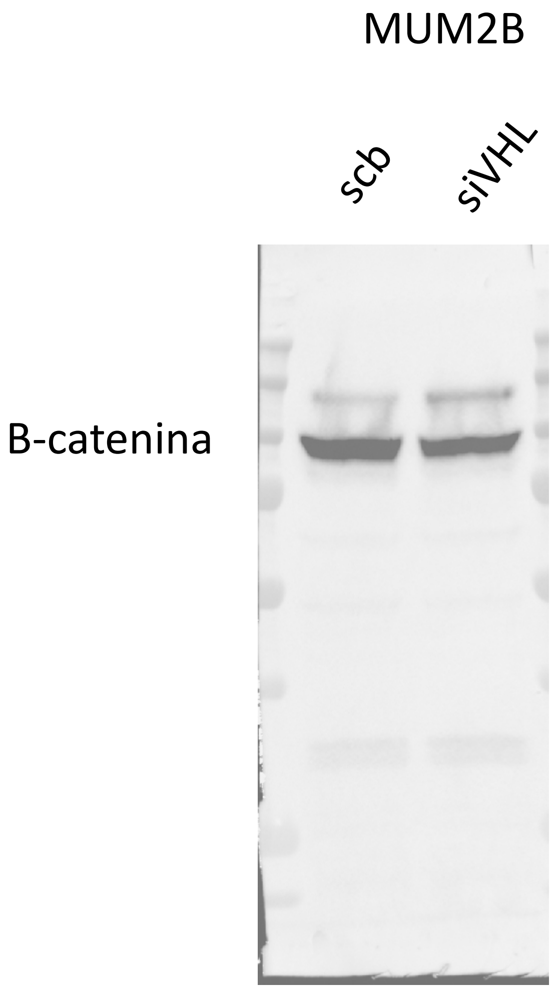

06/05/2024

MUM2B

scb siVHL

p120

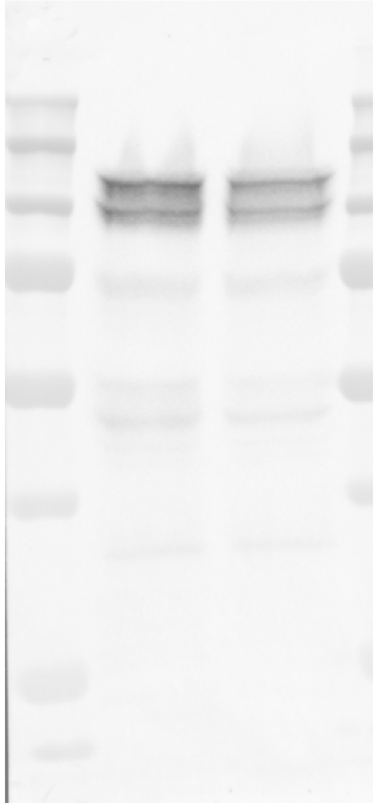

06/05/2024

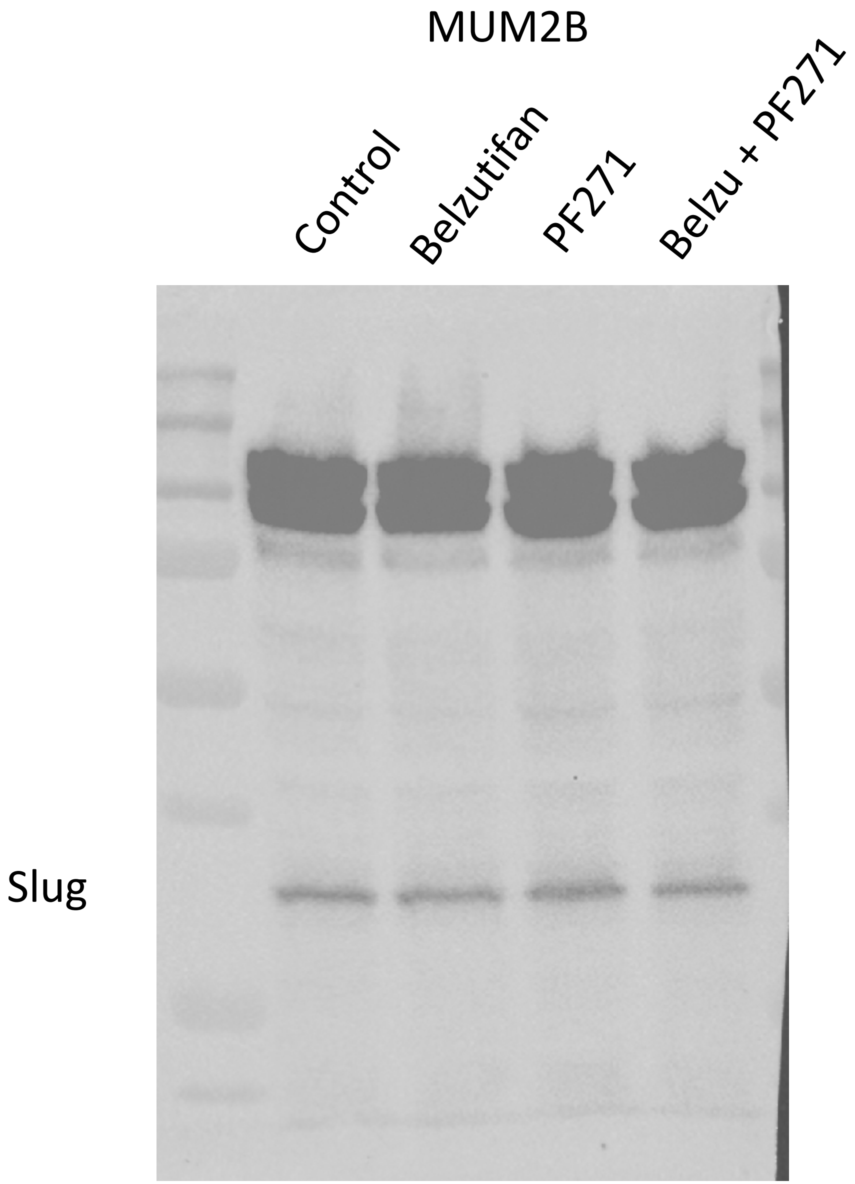

07/05/2024

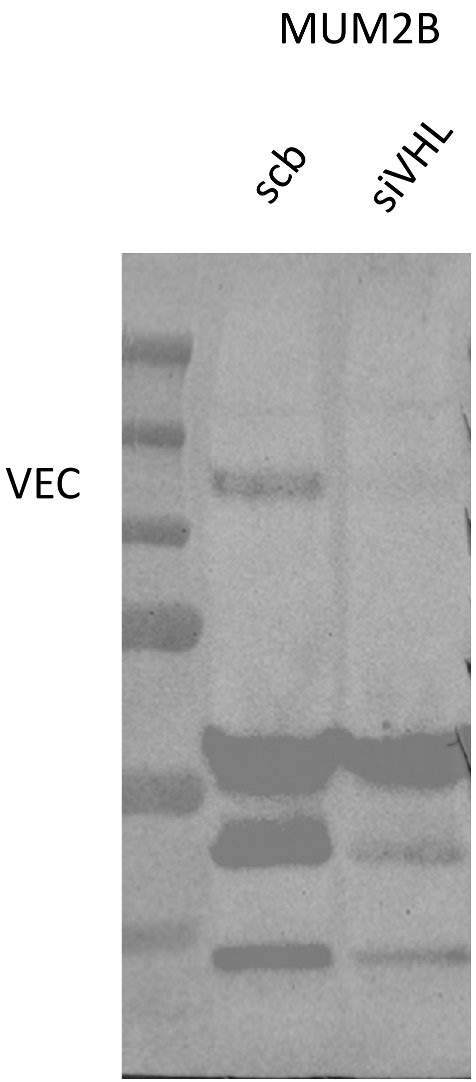

07/05/2024

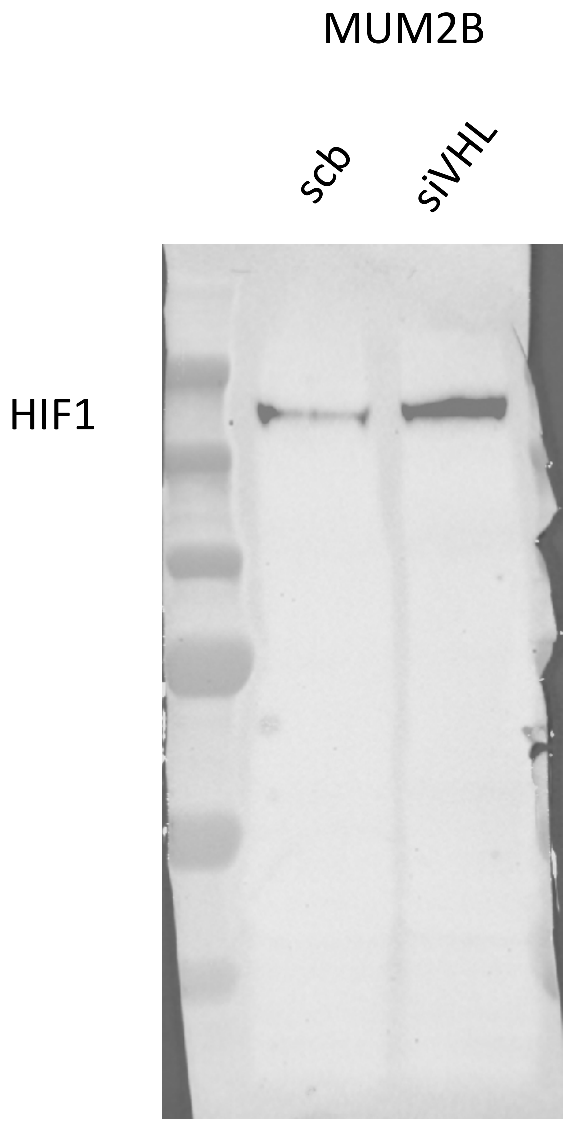

07/05/2024

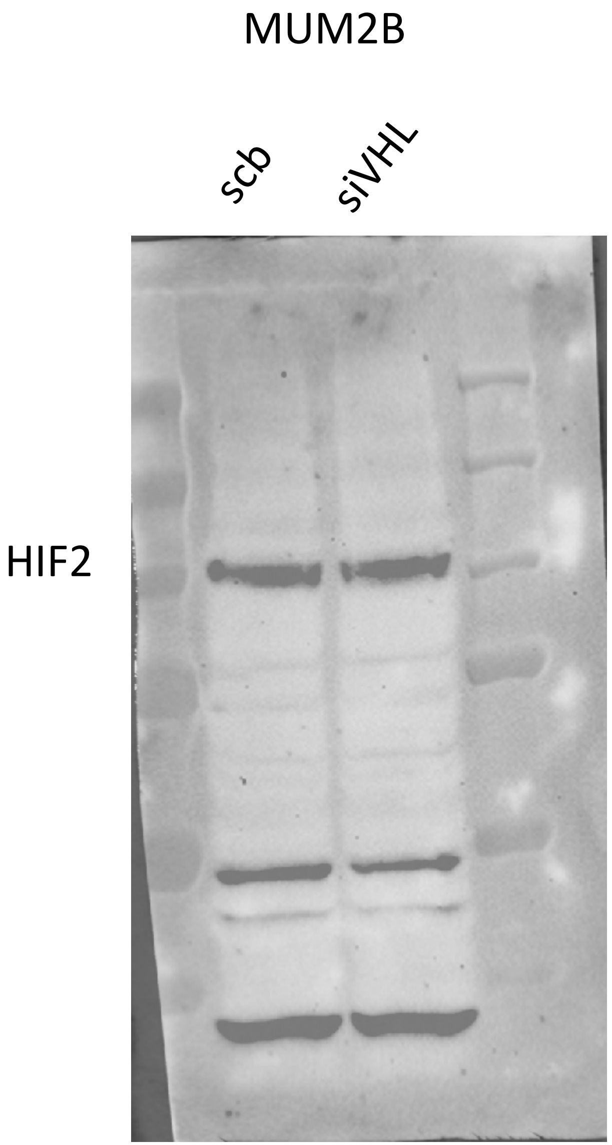

06/05/2024

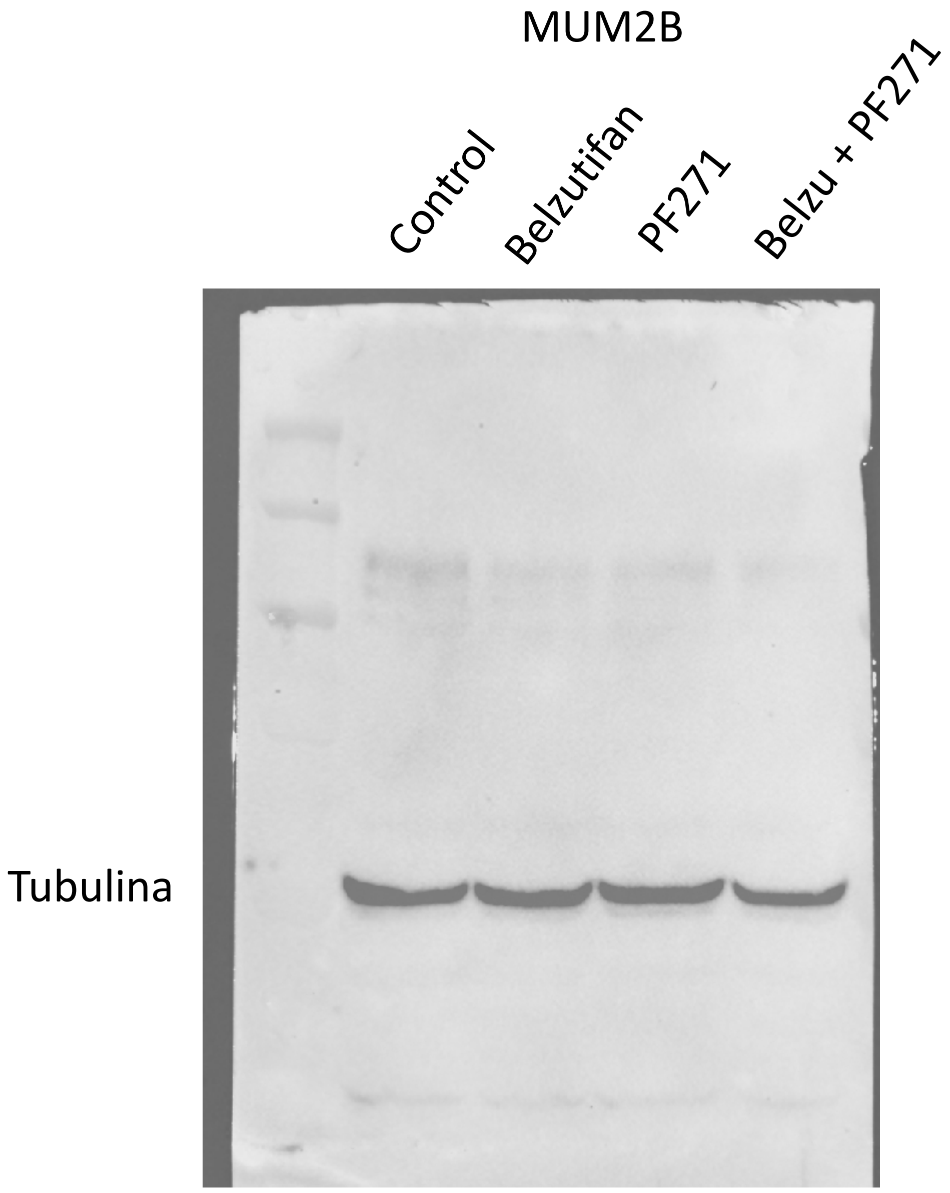

06/05/2024

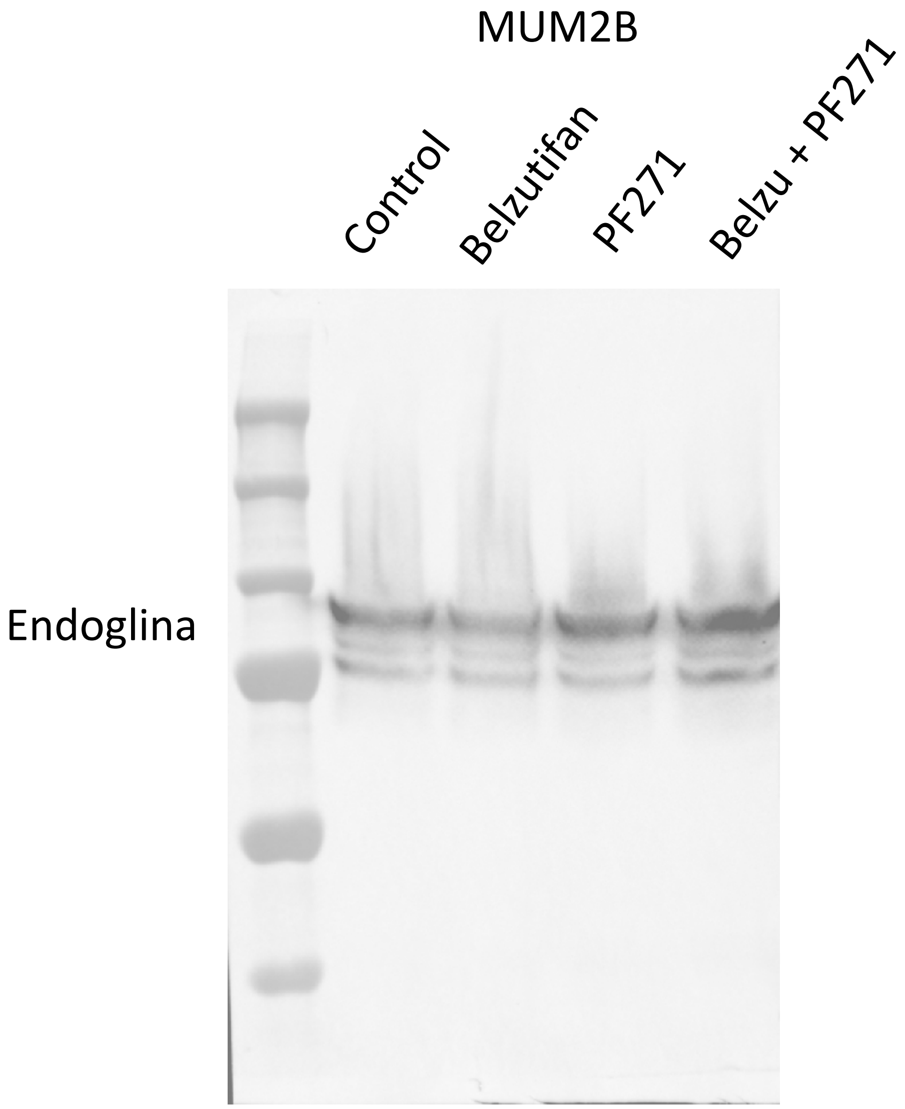

06/05/2024

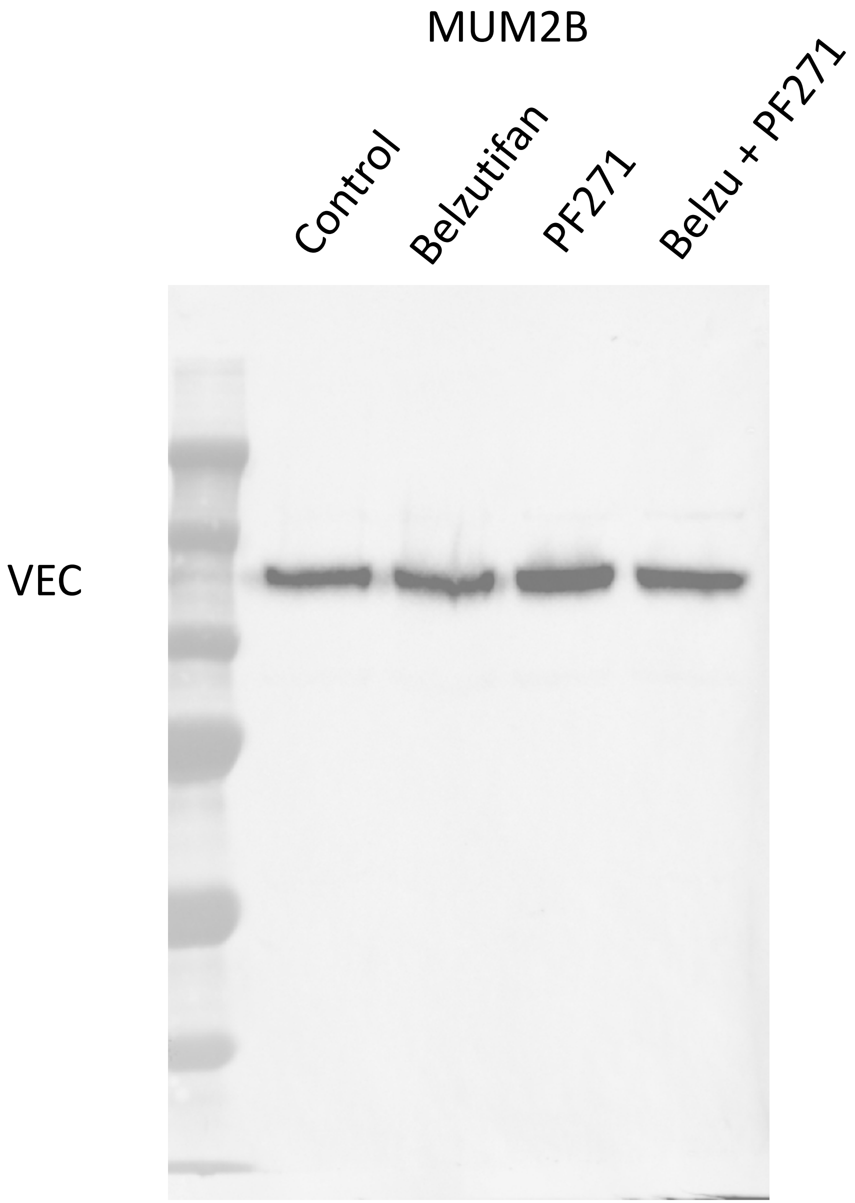

08/05/2024

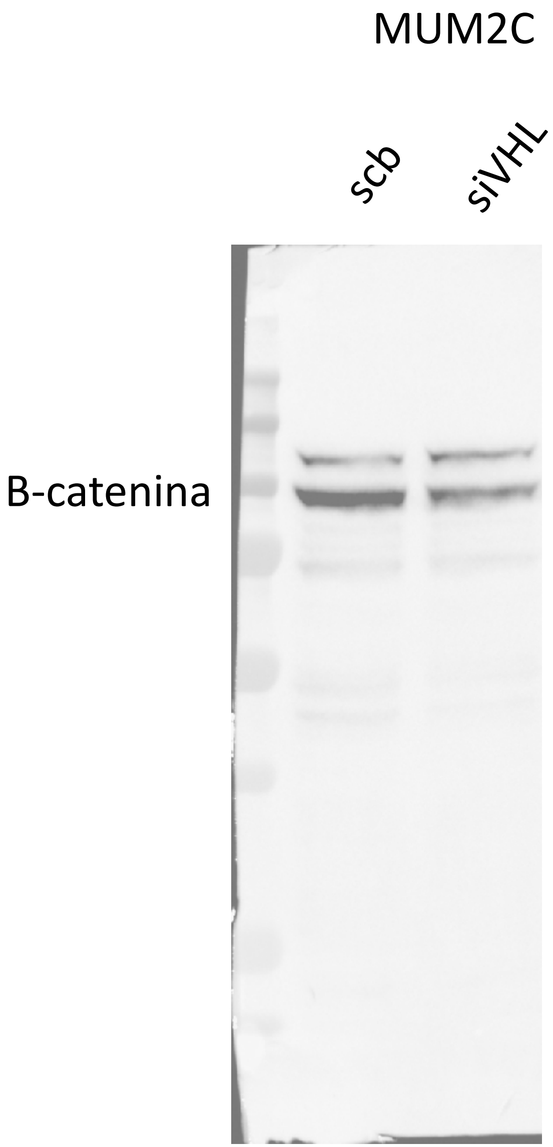

08/05/2024

MUM2B

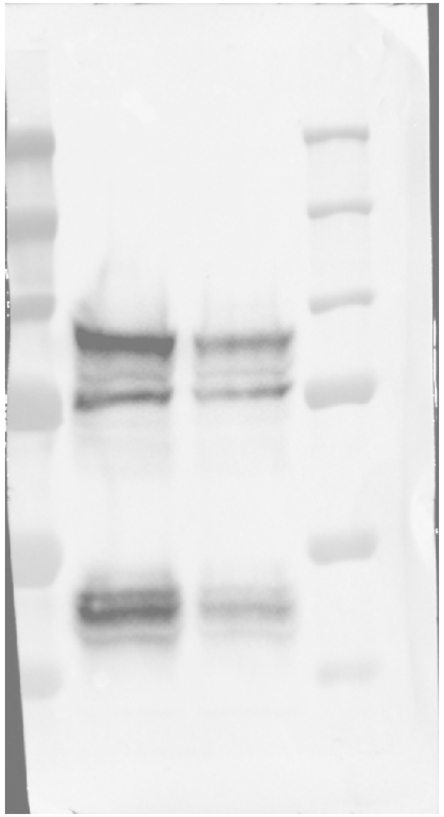

08/05/2024

MUM2B

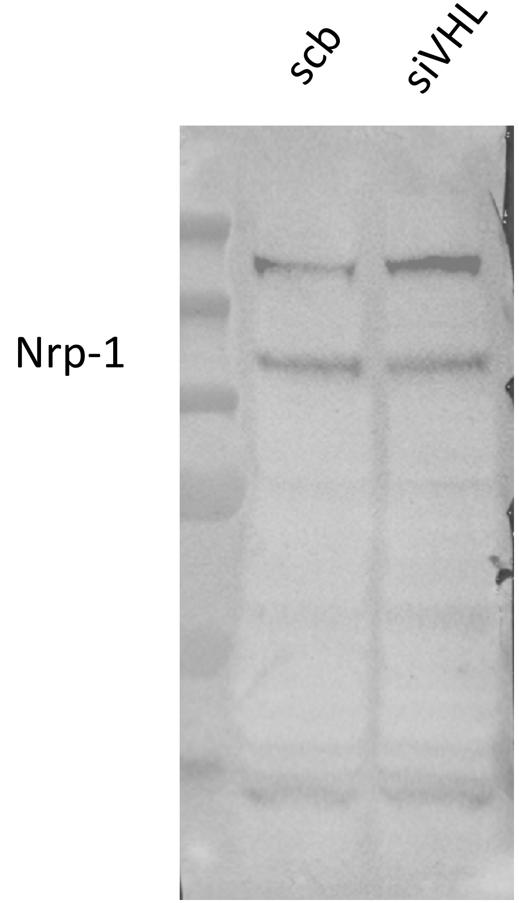

08/05/2024

MUM2B

Normoxia Hipoxia

Y397 FAK

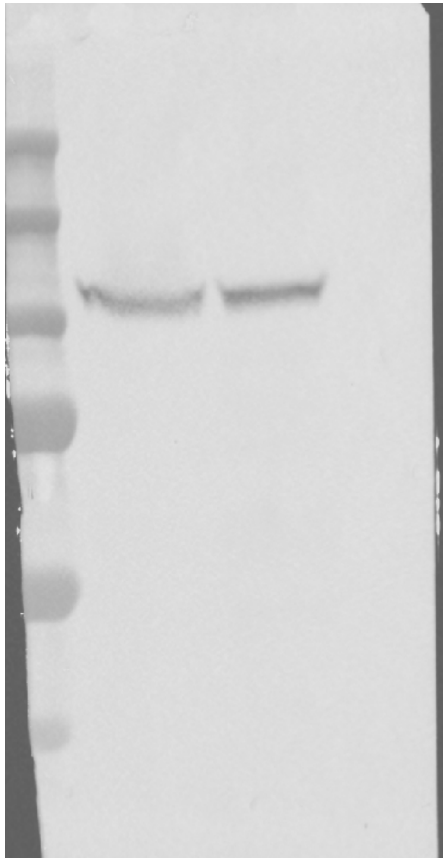

08/05/2024

MUM2B

Normoxia Hipoxia

Y658 VEC

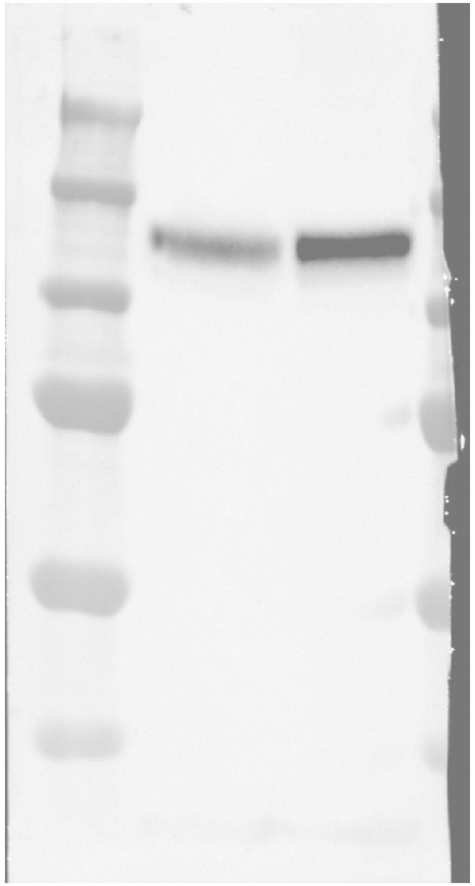

08/05/2024

MUM2B

Normoxia Hipoxia

HIF1-a

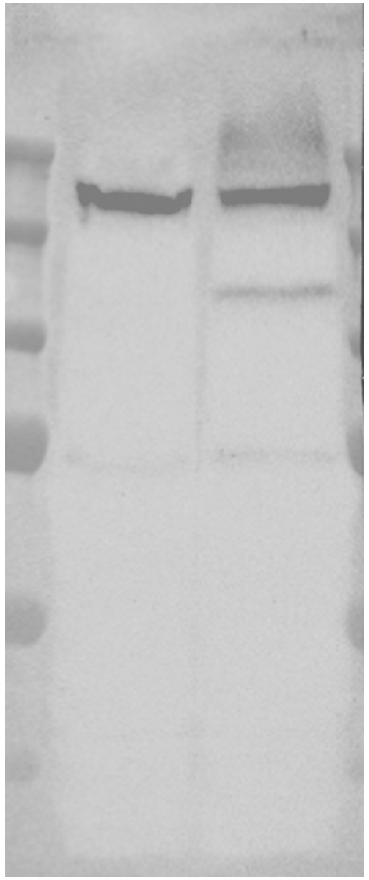

08/05/2024

MUM2B

Normoxia Hipoxia

HIF2-a

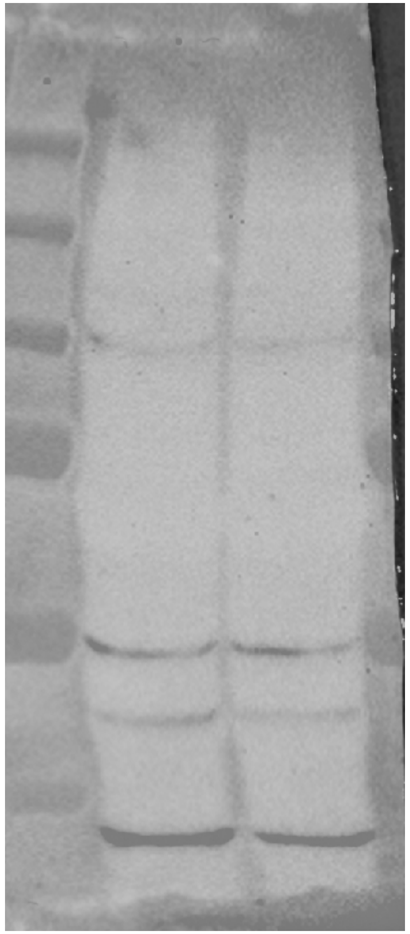

08/05/2024

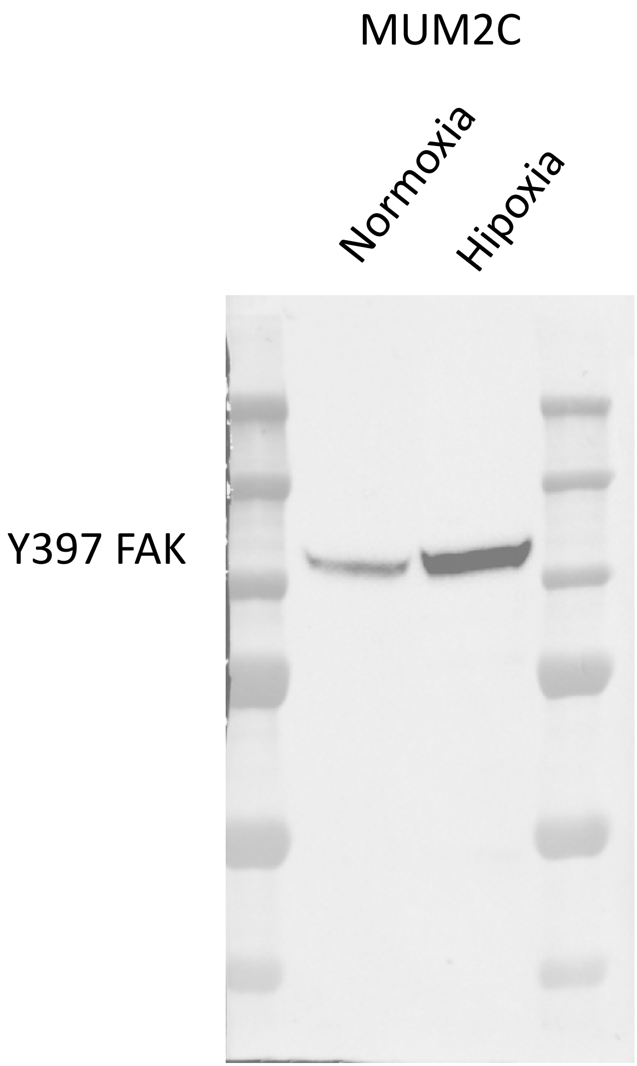

08/05/2024

MUM2C

Normoxia Hipoxia

Y658 VEC

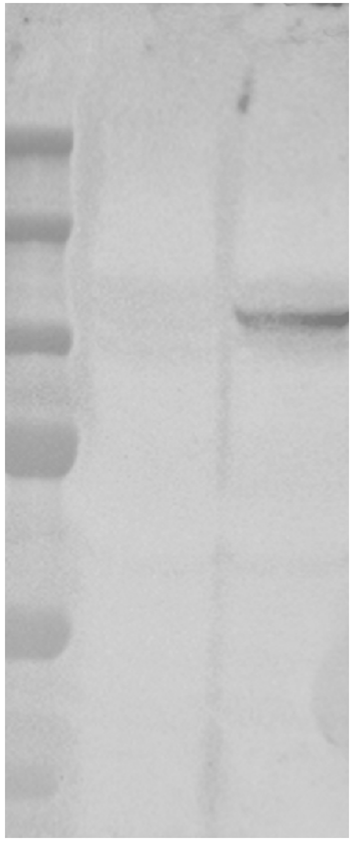

08/05/2024

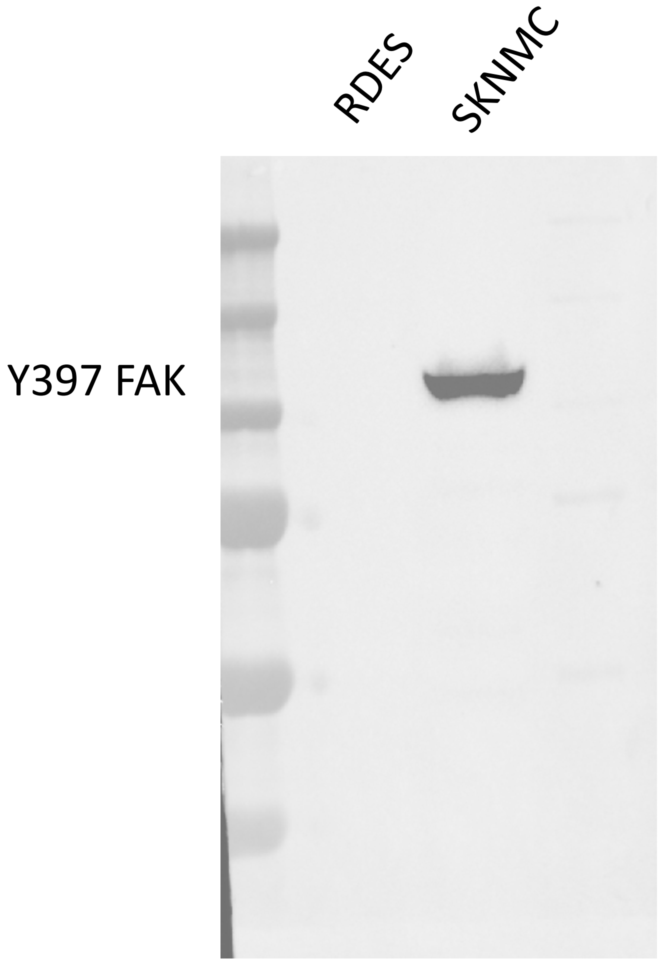

08/05/2024

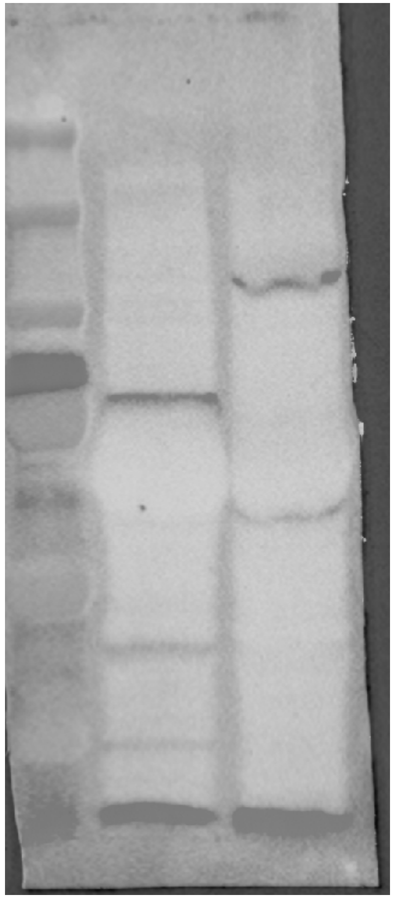

09/05/2024

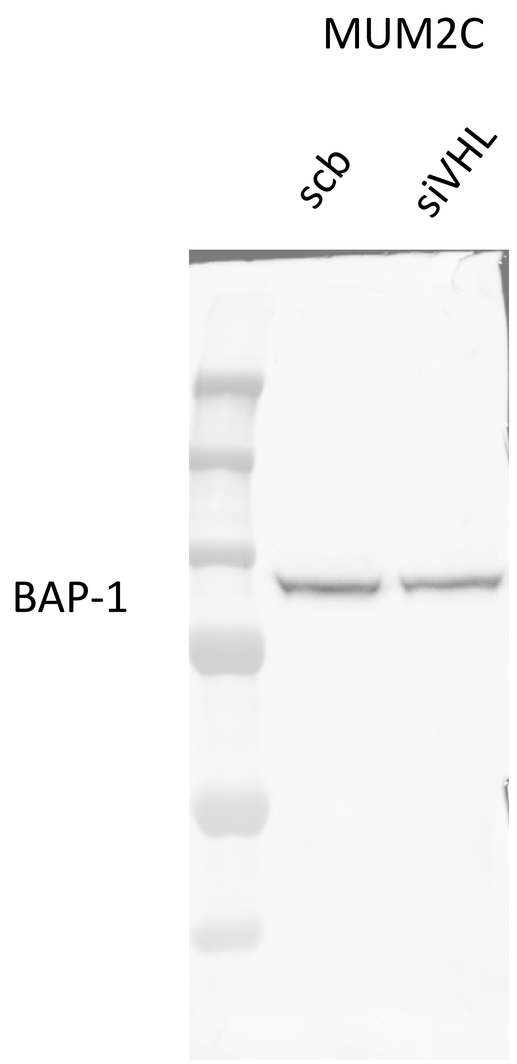

09/05/2024

MUM2C

scb siVHL

Y397 FAK

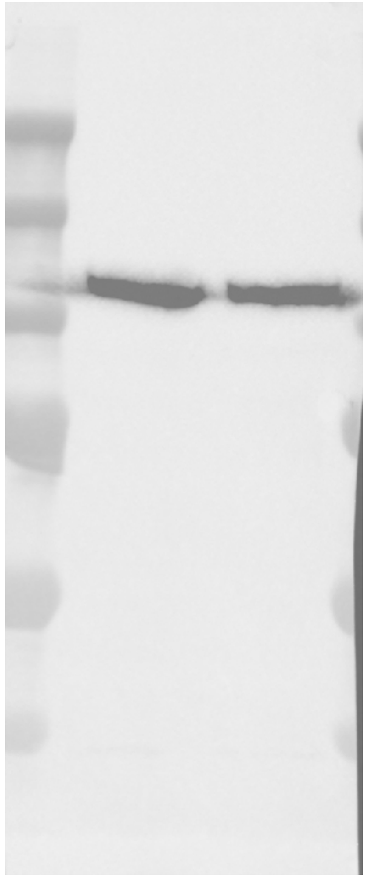

09/05/2024

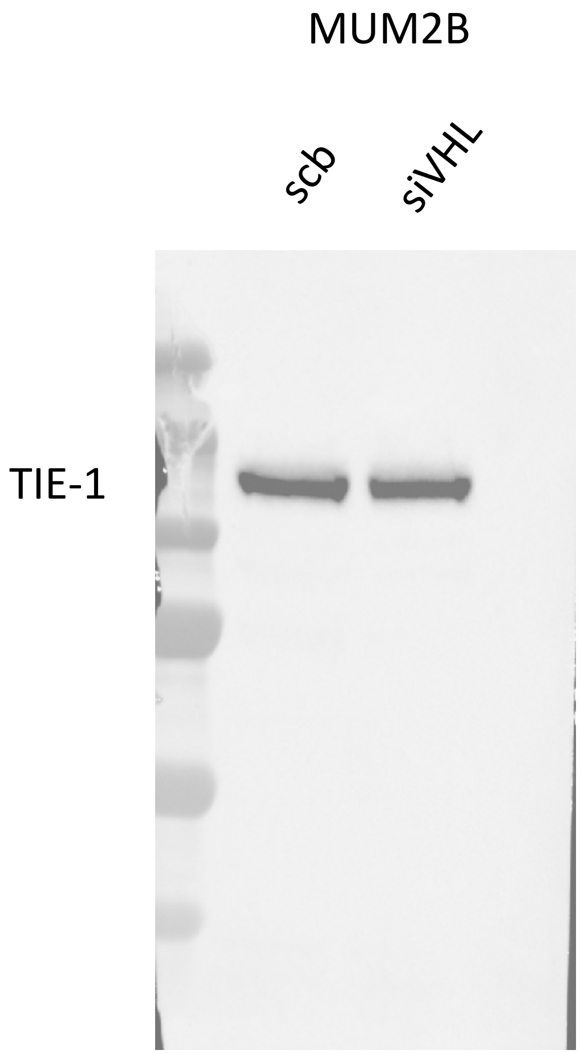

09/05/2024

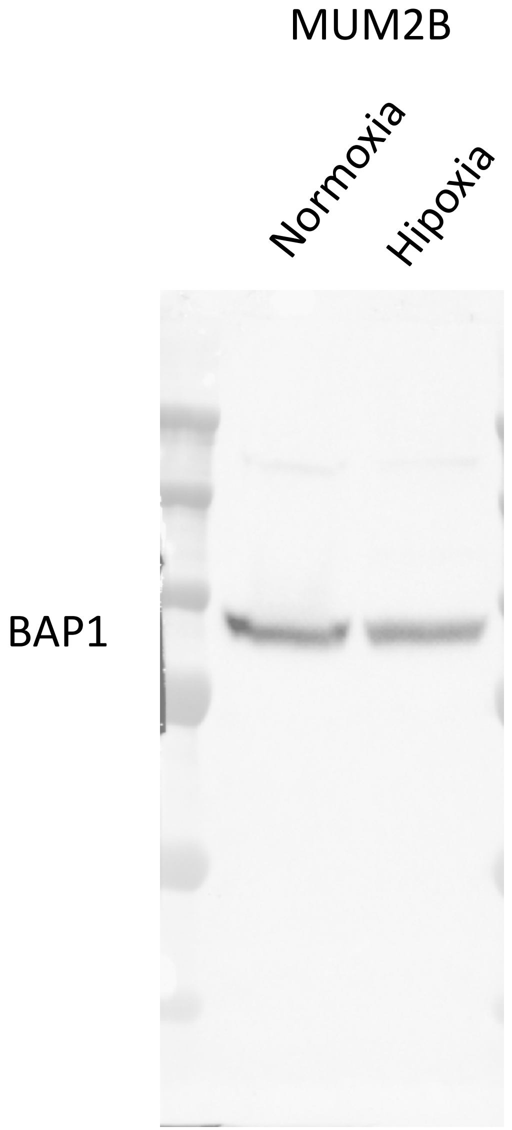

09/05/2024

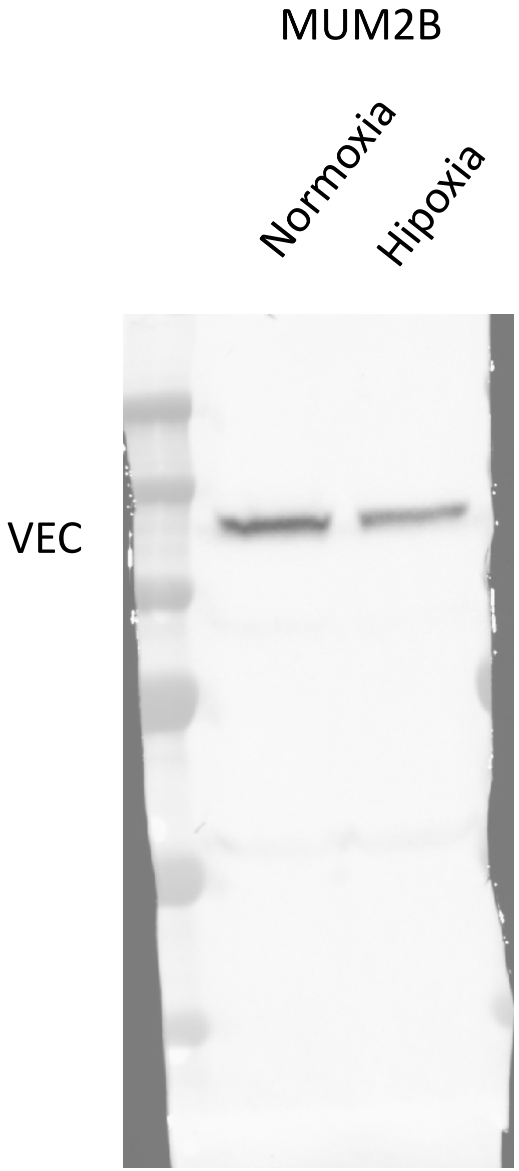

09/05/2024

MUM2B

Normoxia Hipoxia

Twist1

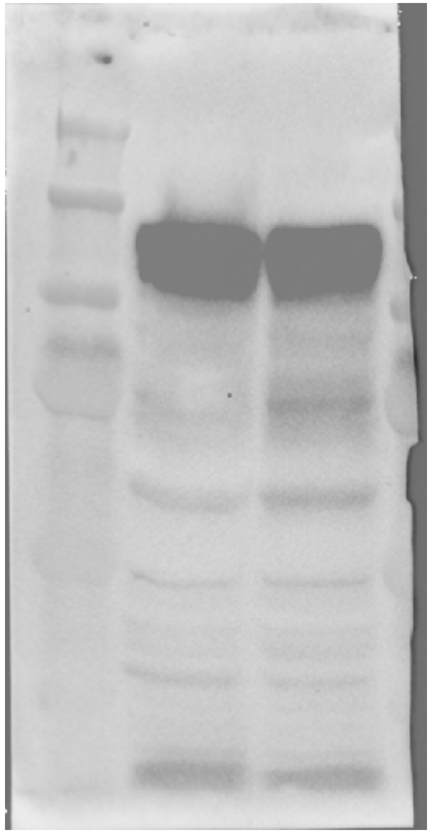

09/05/2024

MUM2B

Control
Belzutifan
PF271
Belzu + PF271

VEC
b-catenina

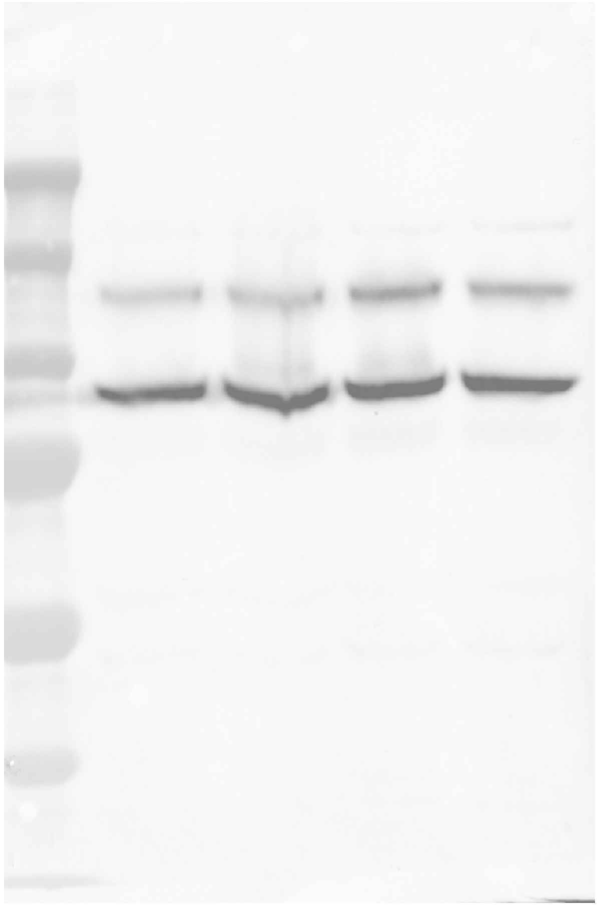

09/05/2024

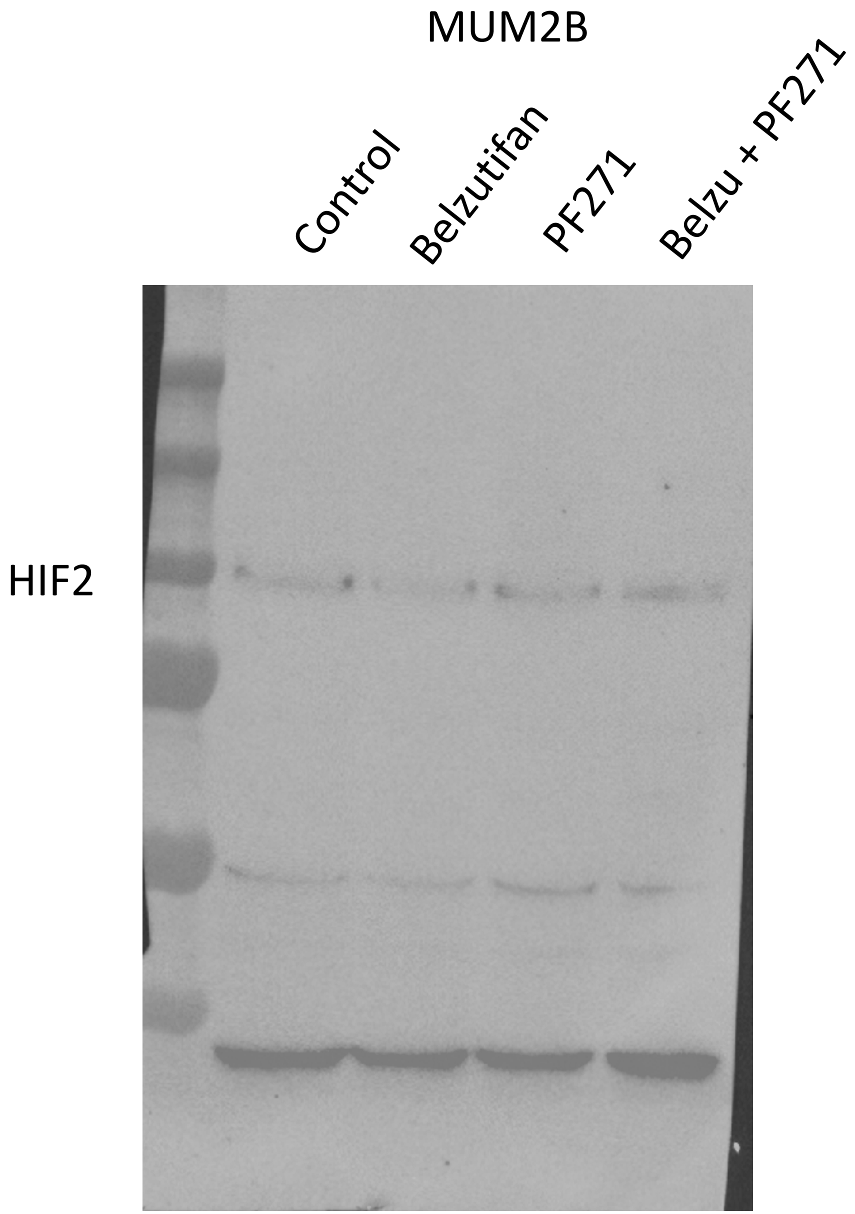

10/05/2024

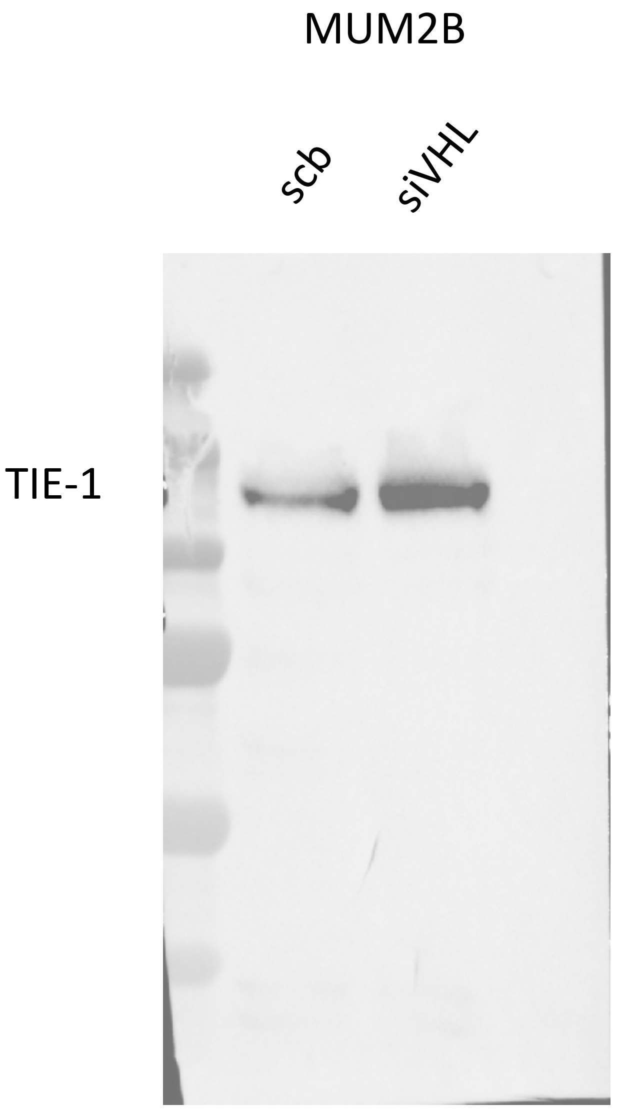

10/05/2024

MUM2B

Normoxia Hipoxia

B-catenina

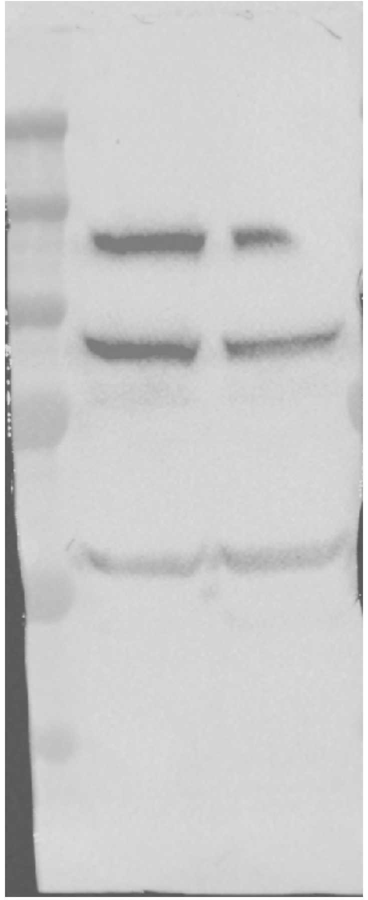

10/05/2024

MUM2B

Normoxia Hipoxia

TIE-1

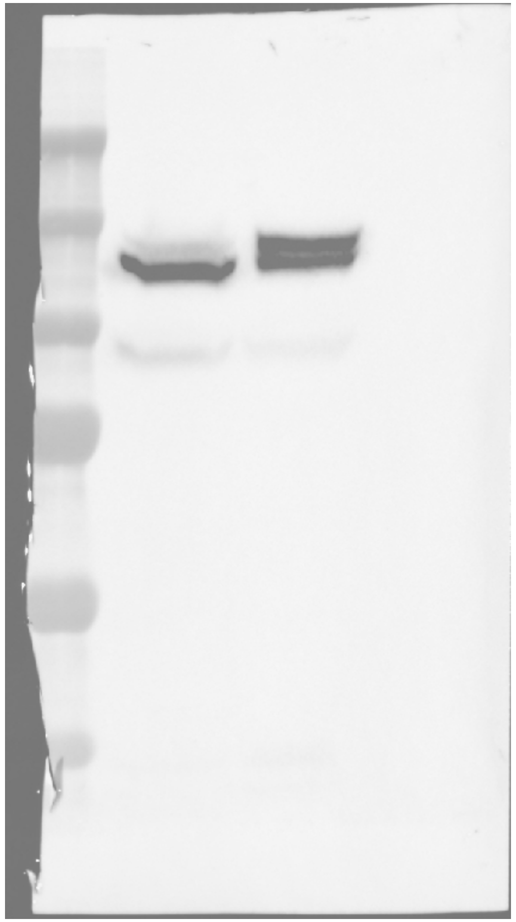

10/05/2024

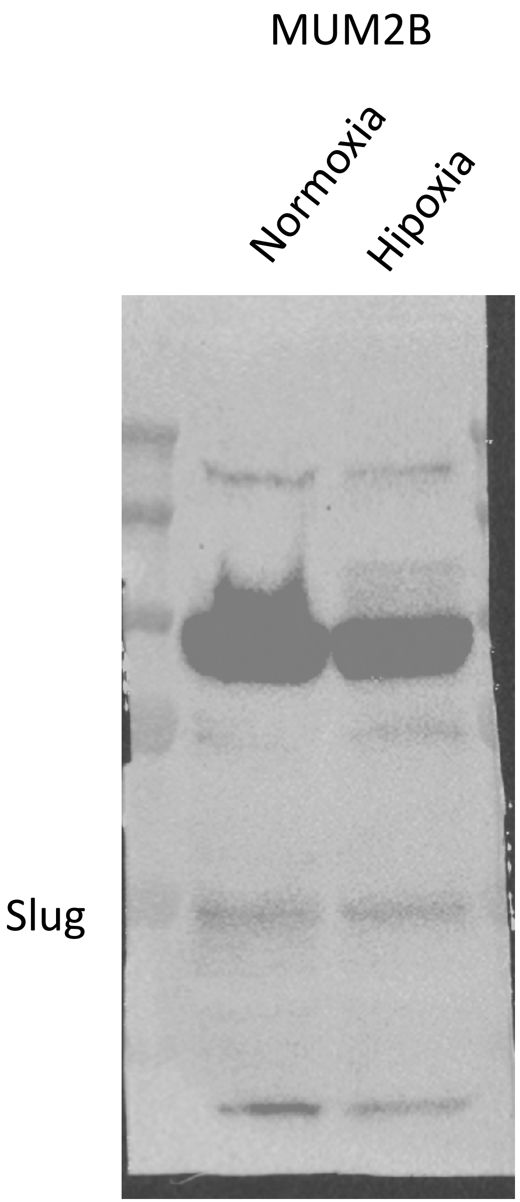

10/05/2024

MUM2B

Normoxia Hipoxia

HIF2

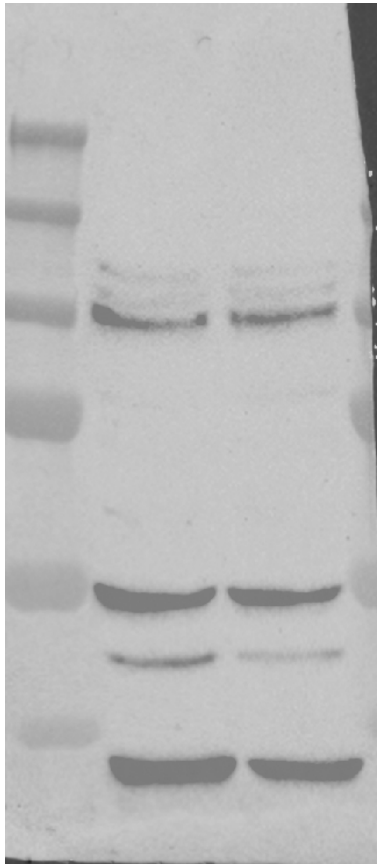

10/05/2024

MUM2B

Control Belzutifan PF271 Belzu + PF271

FAK

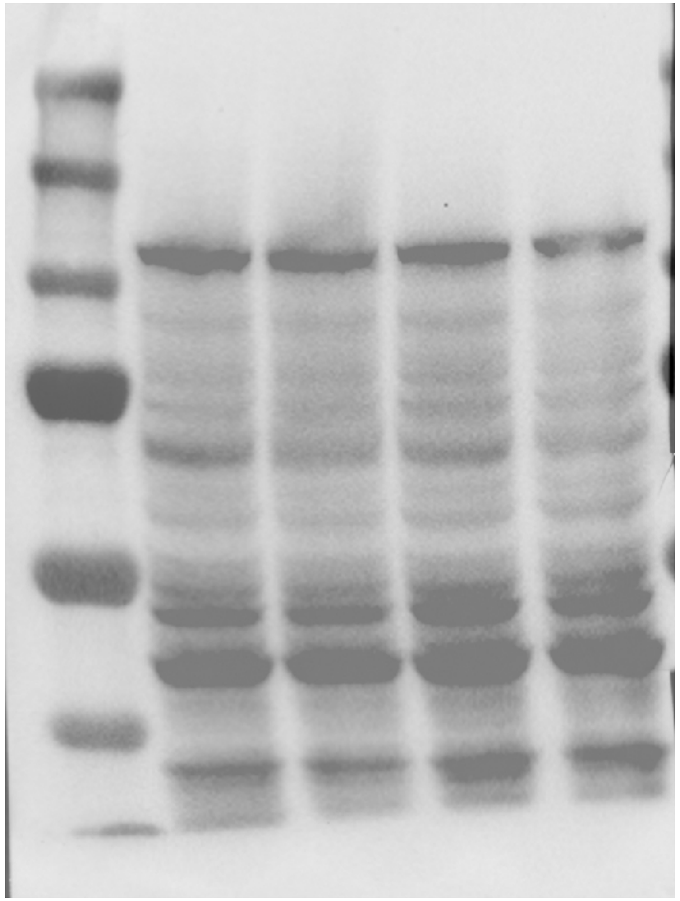

10/05/2024

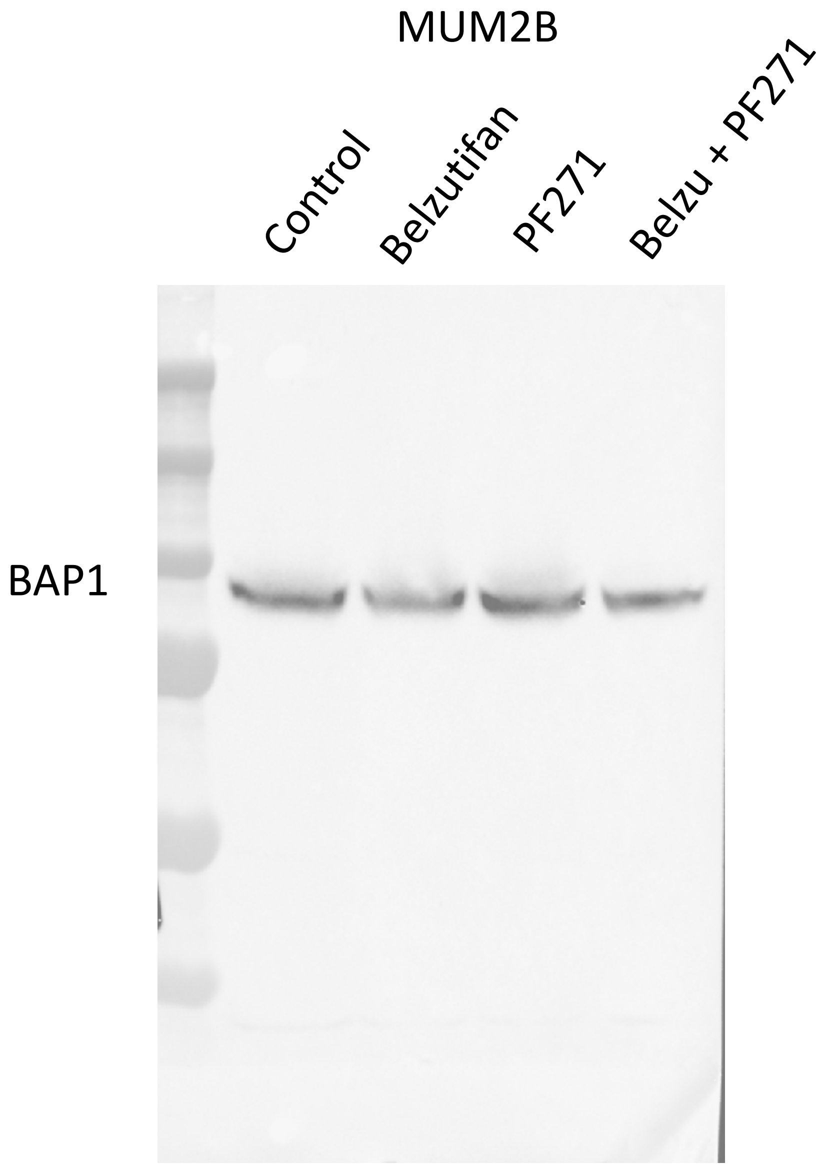

13/05/2024

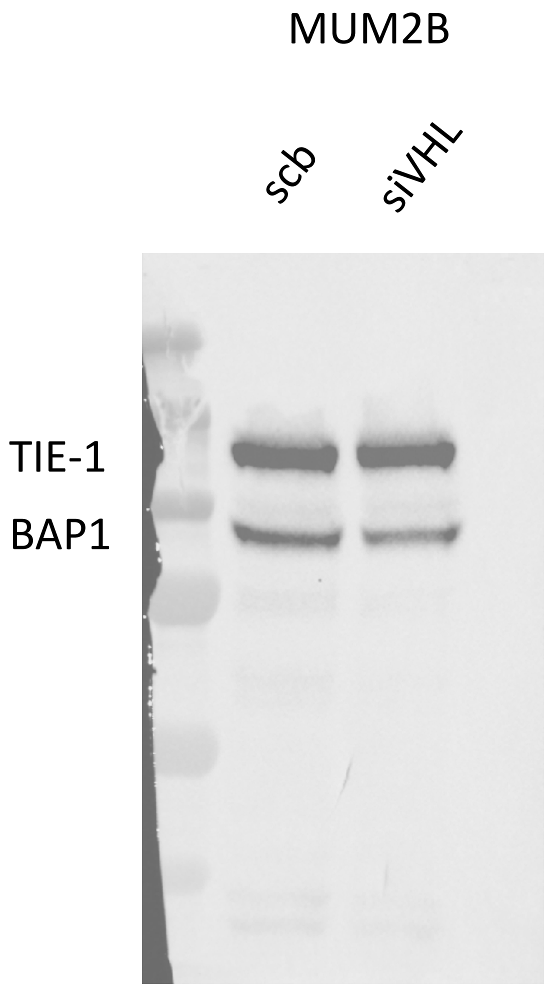

13/05/2024

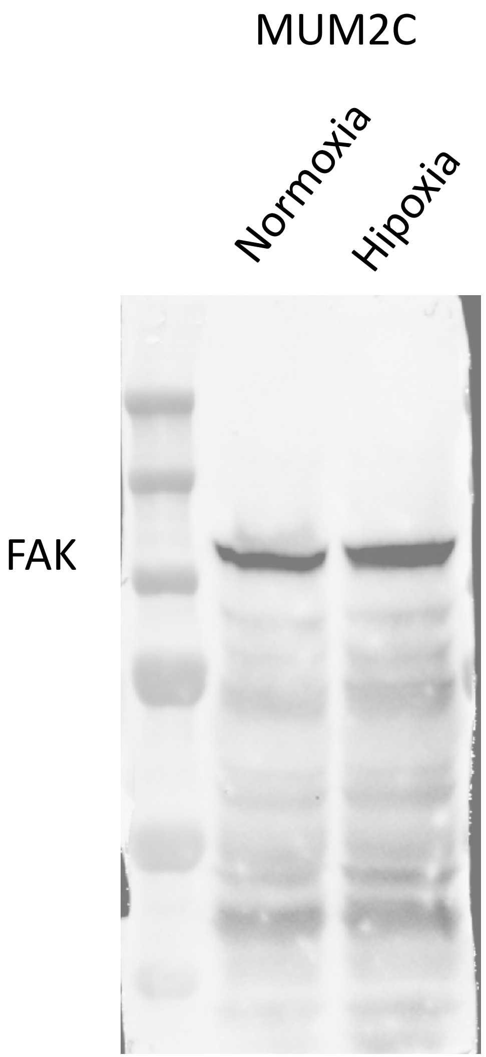

13/05/2024

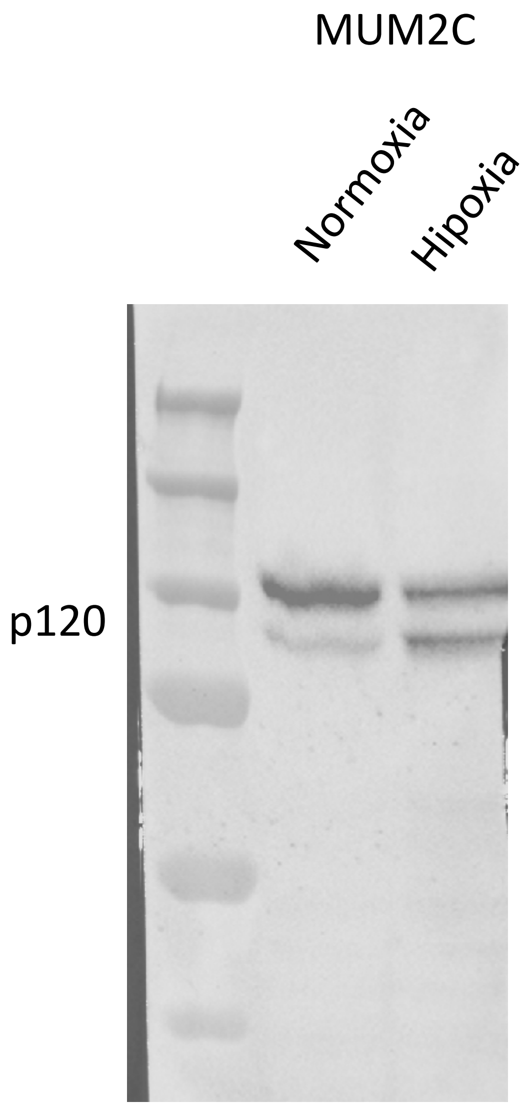

13/05/2024

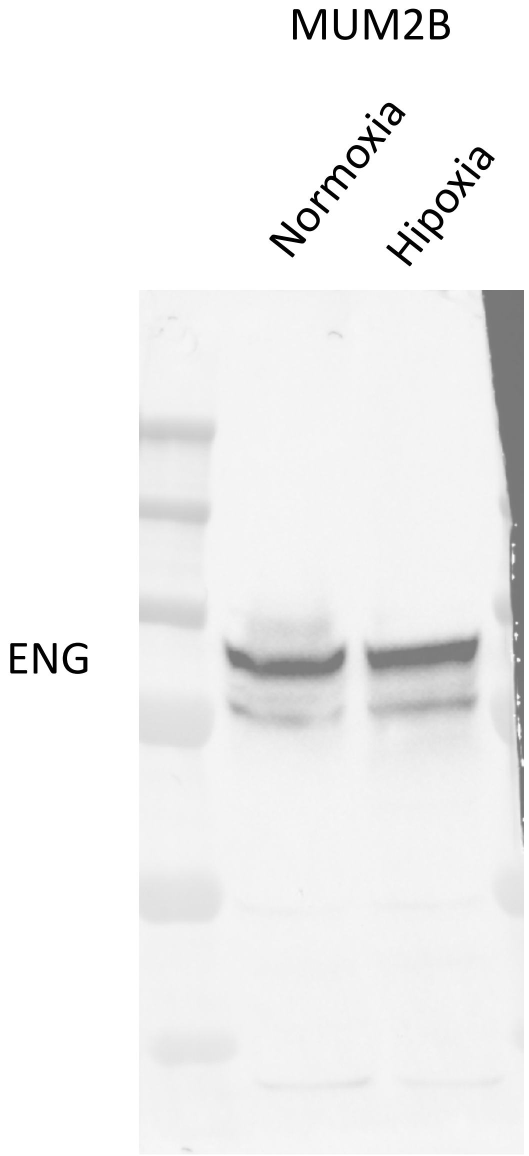

13/05/2024

MUM2B

Normoxia Hipoxia

p120

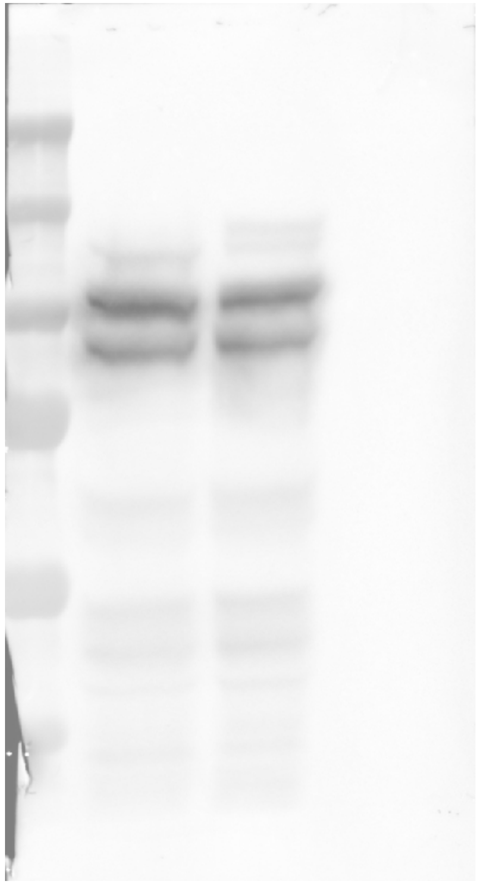

13/05/2024

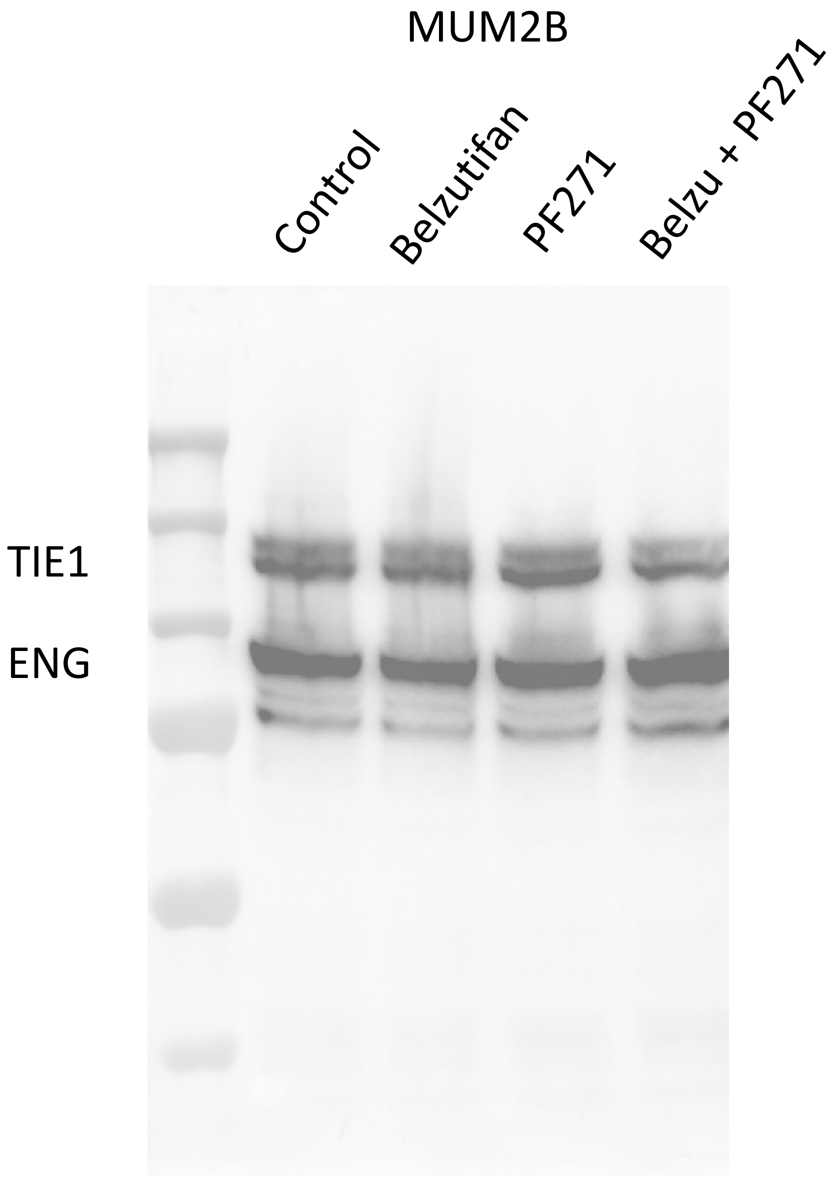

14/05/2024

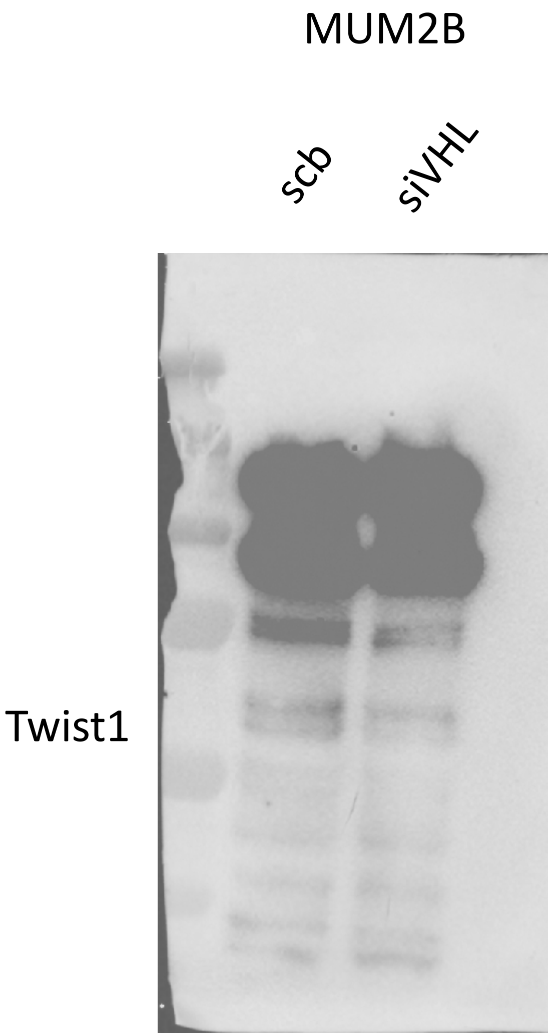

14/05/2024

MUM2B

Normoxia Hipoxia

FAK

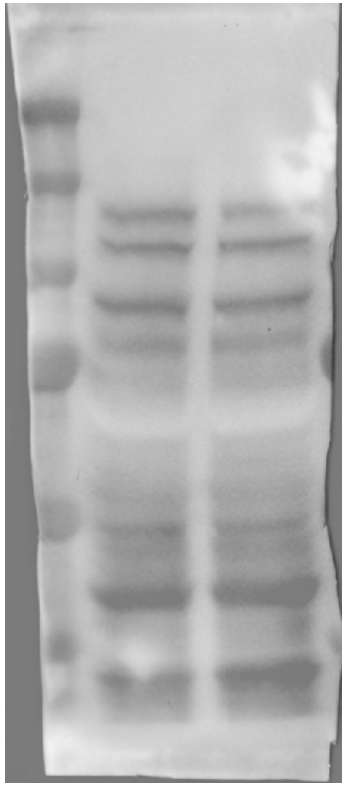

14/05/2024

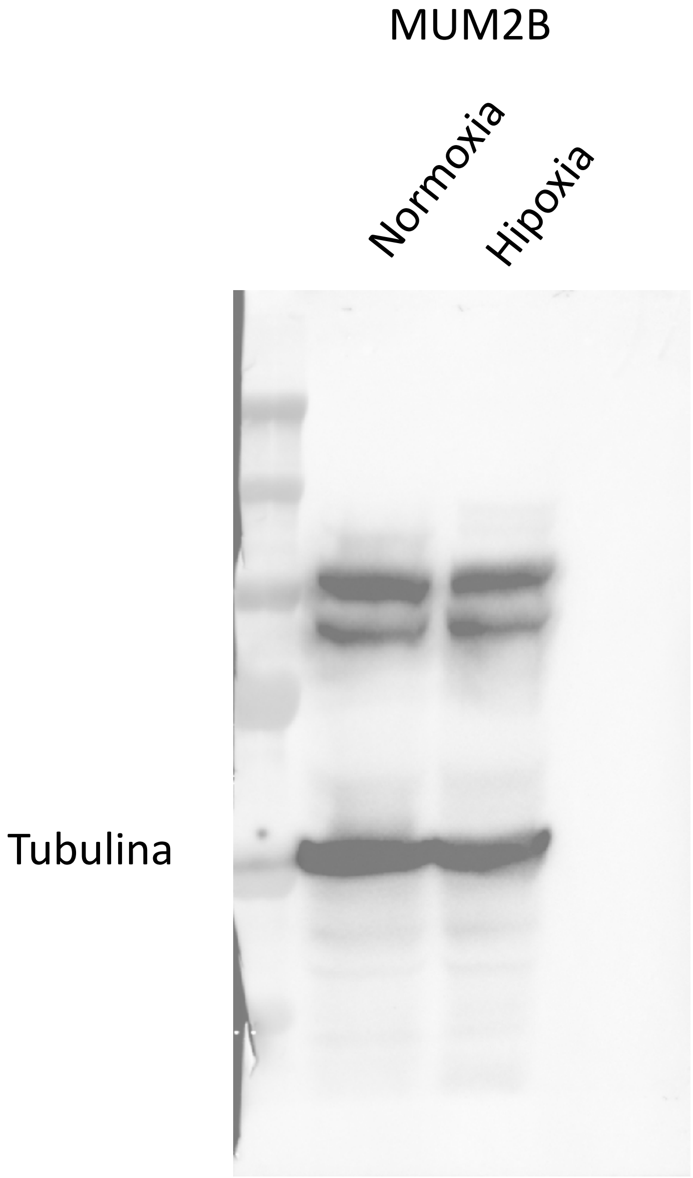

14/05/2024

MUM2C

Normoxia Hipoxia

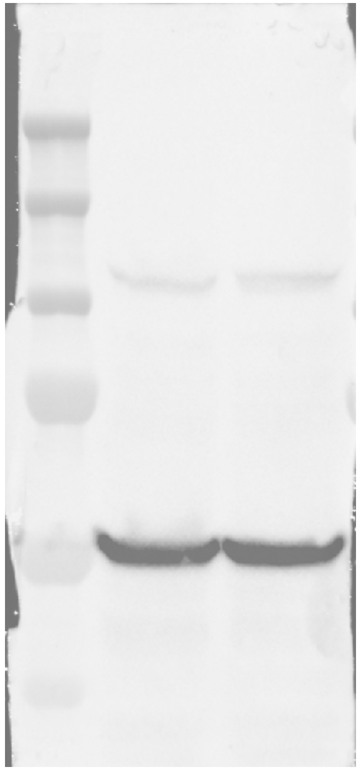

Tubulina

22/05/2024

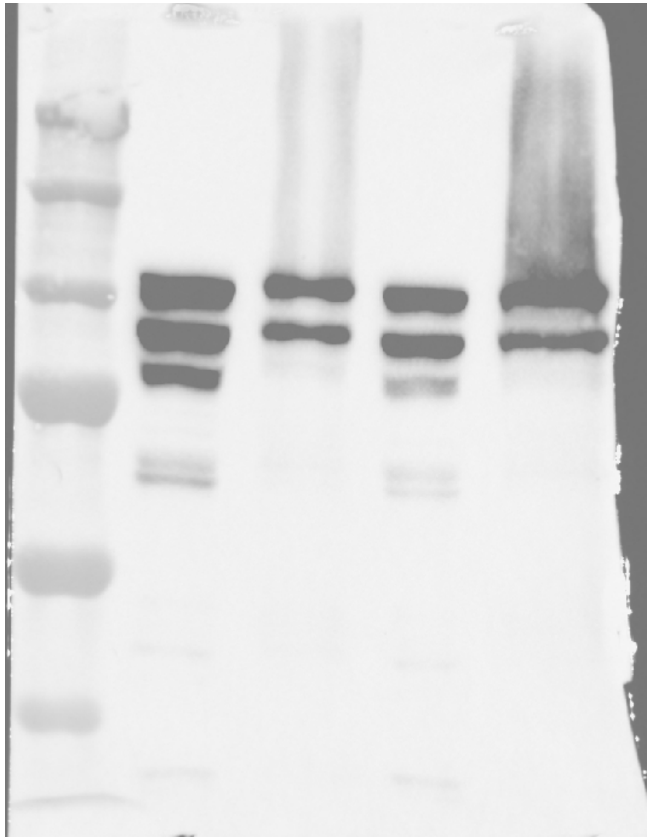

22/05/2024

MUM2B

scb siVHL

CE NE CE NE

p120

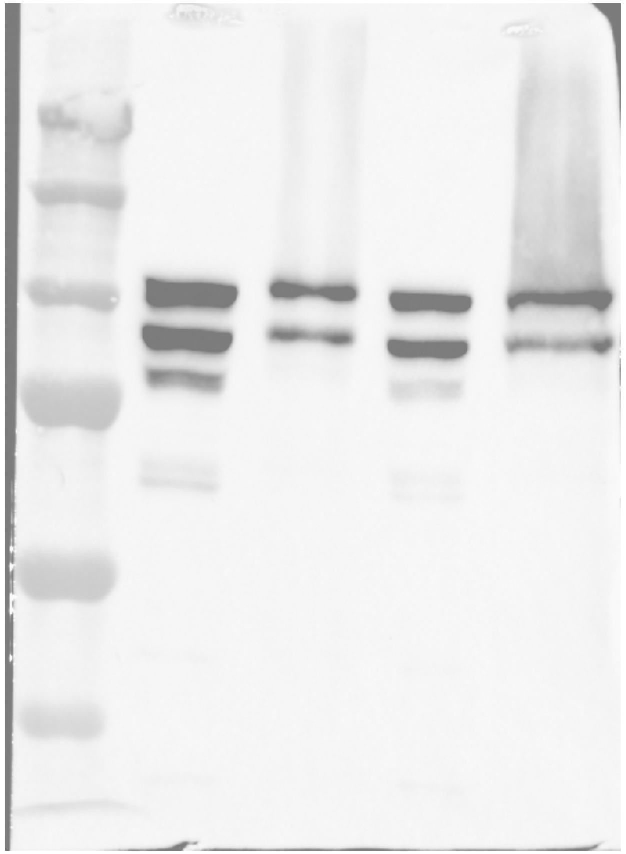

22/05/2024

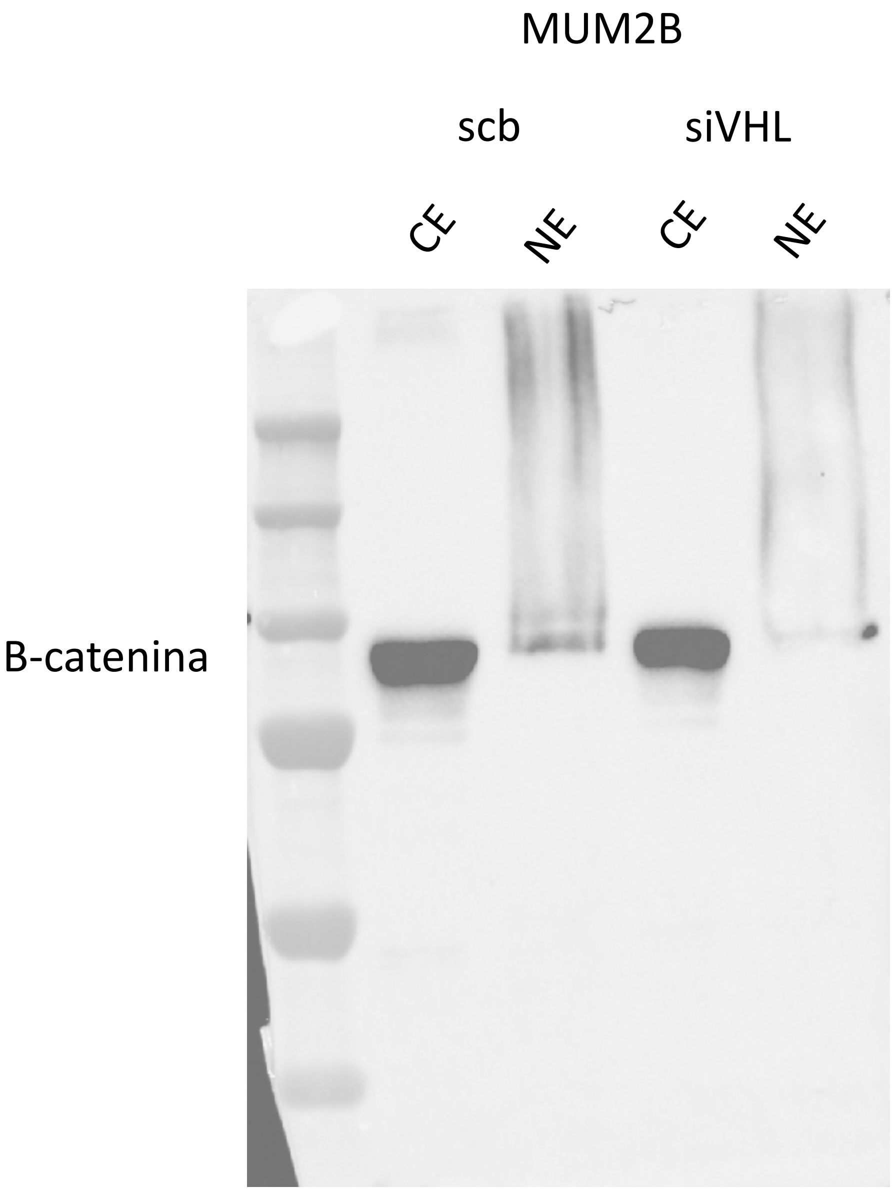

22/05/2024

MUM2B

scb siVHL

CE NE CE NE

B-catenina

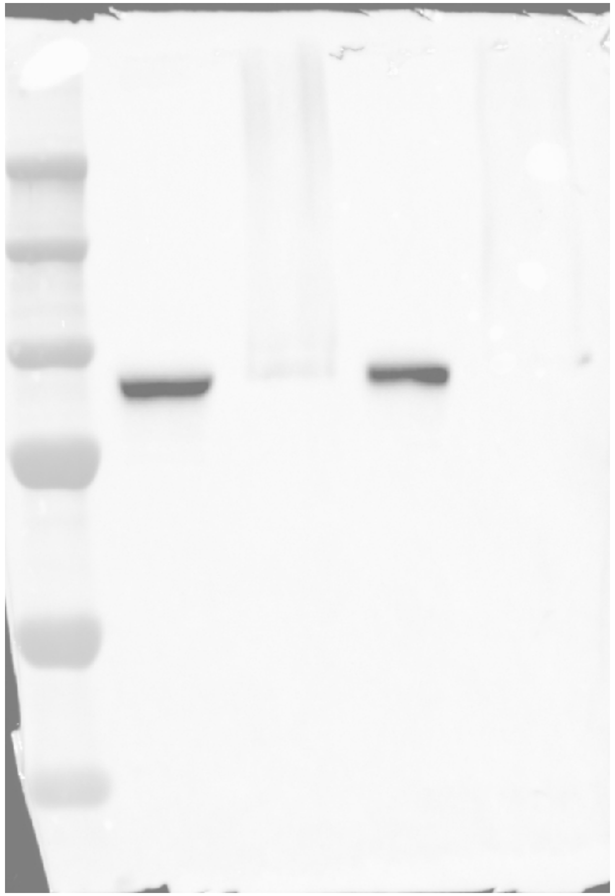

22/05/2024

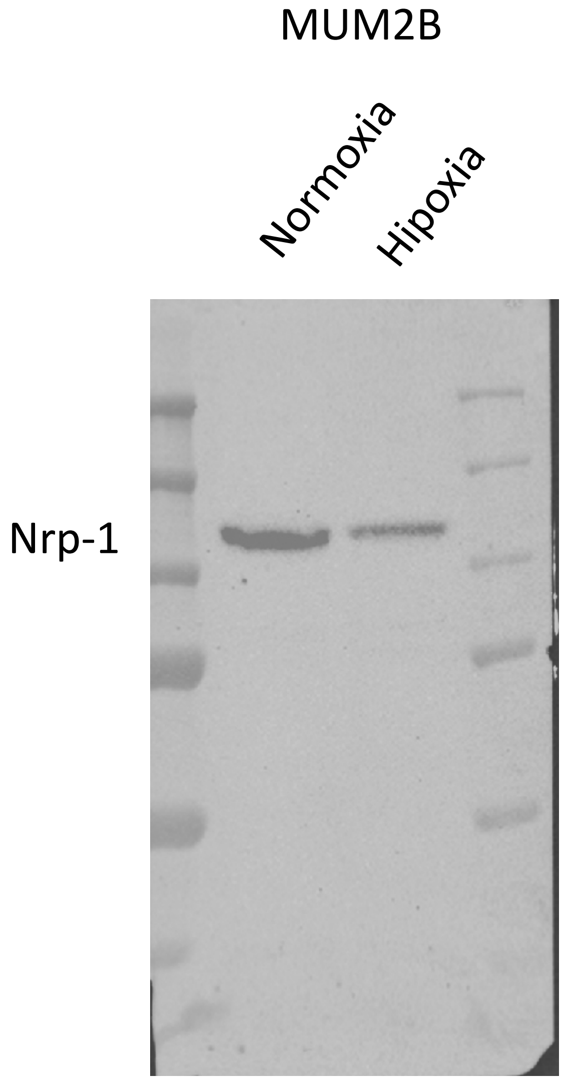

22/05/2024

MUM2C

Normoxia

Hipoxia

HIF1

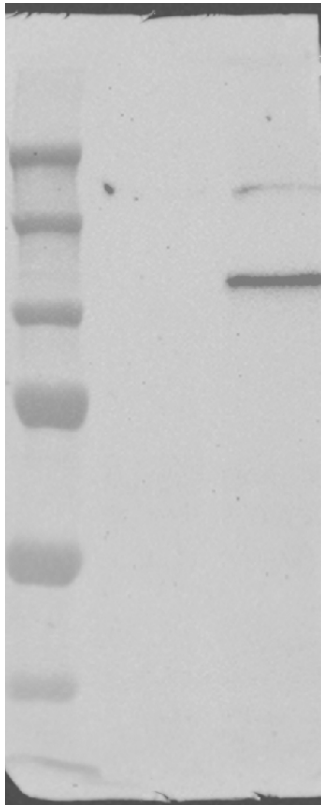

22/05/2024

MUM2B

scb siPHD2

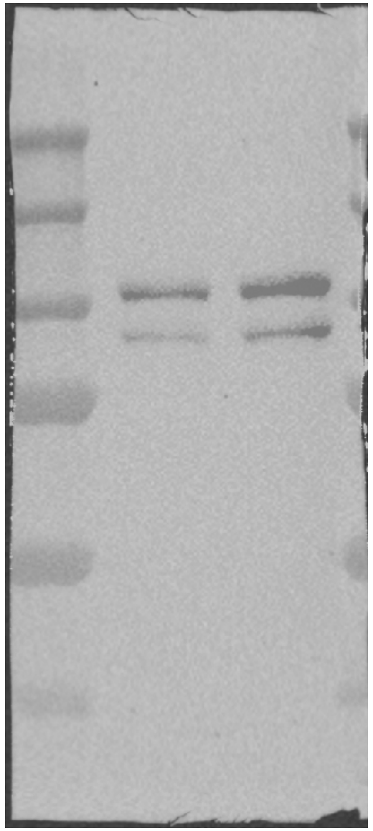

p120

22/05/2024

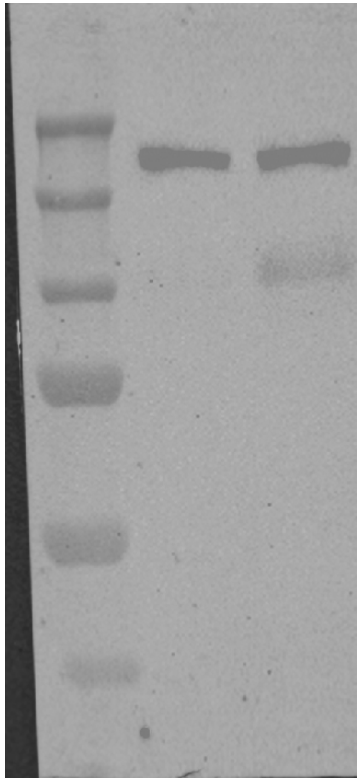

22/05/2024

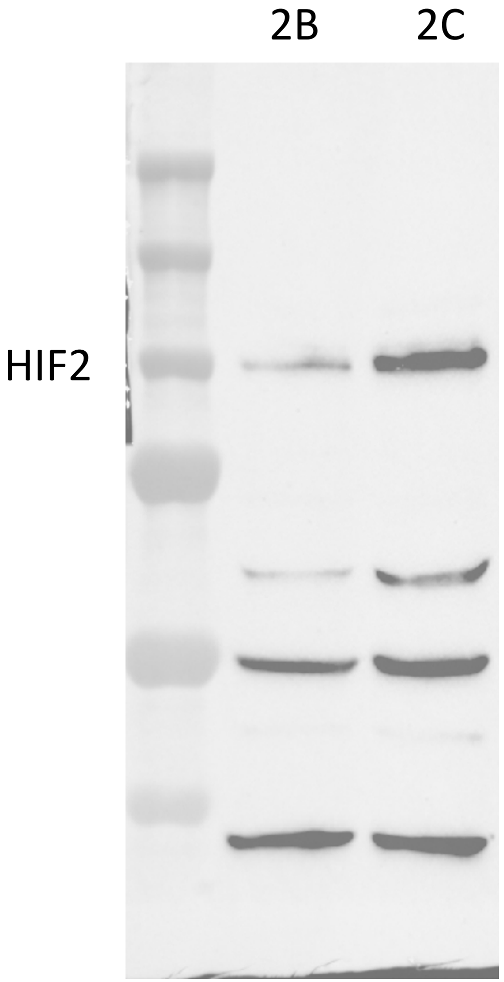

23/05/2024

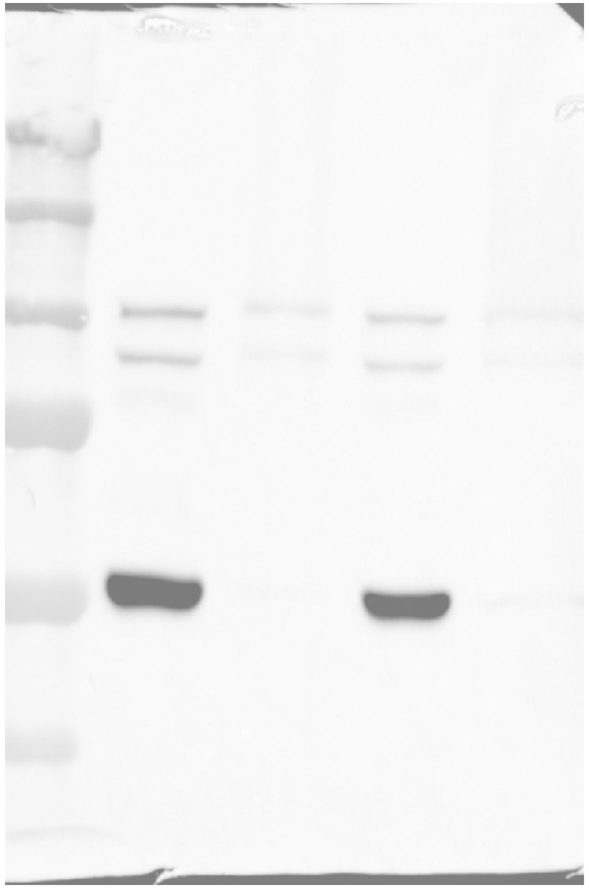

23/05/2024

MUM2B

scb siVHL

CE NE CE NE

PARP-1

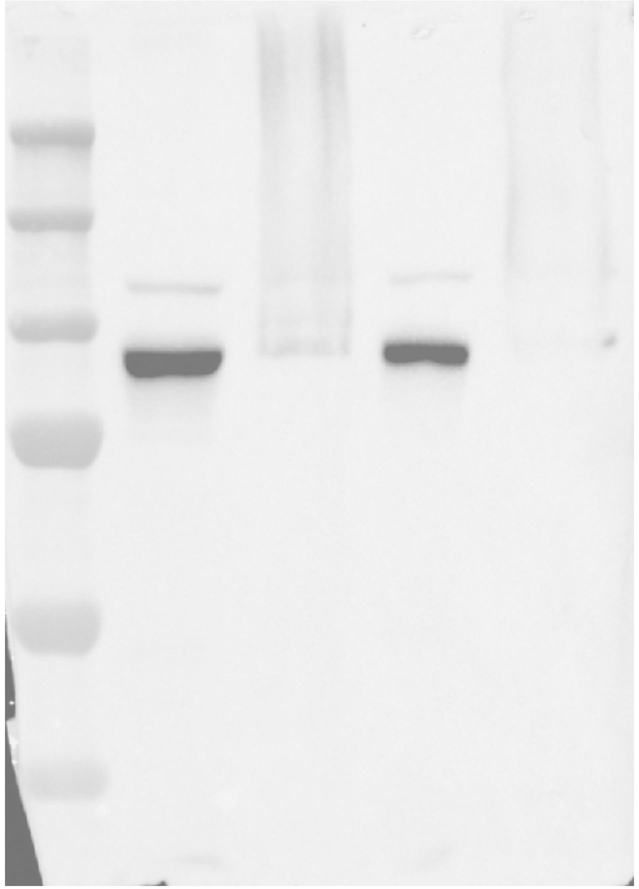

23/05/2024

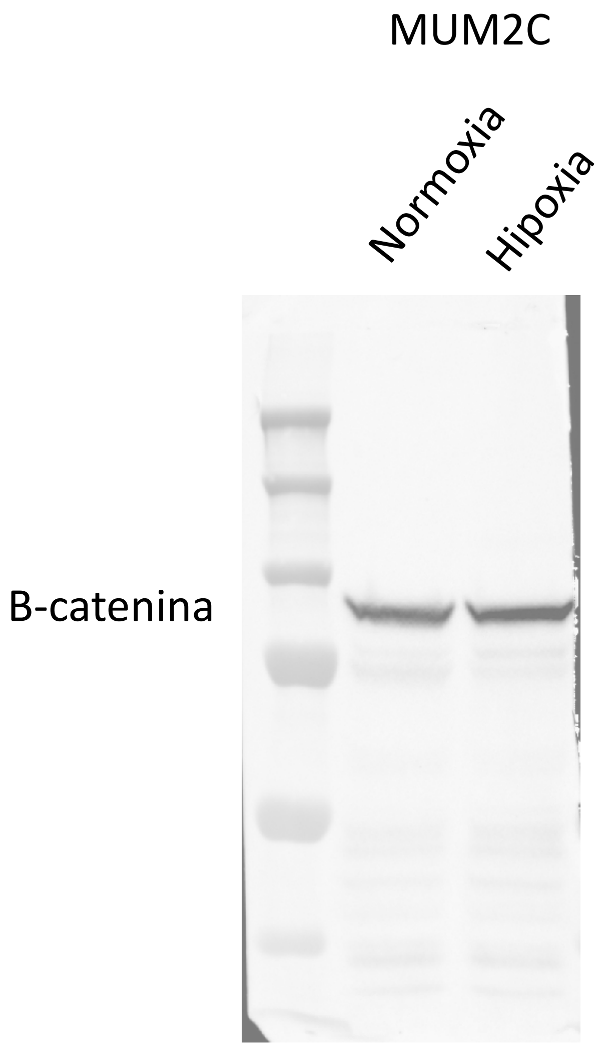

23/05/2024

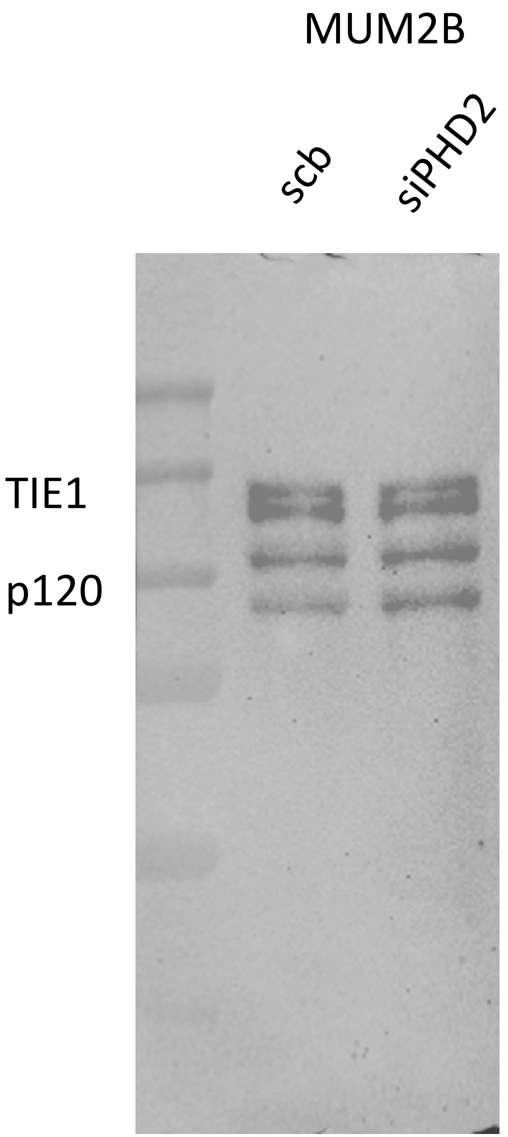

23/05/2024

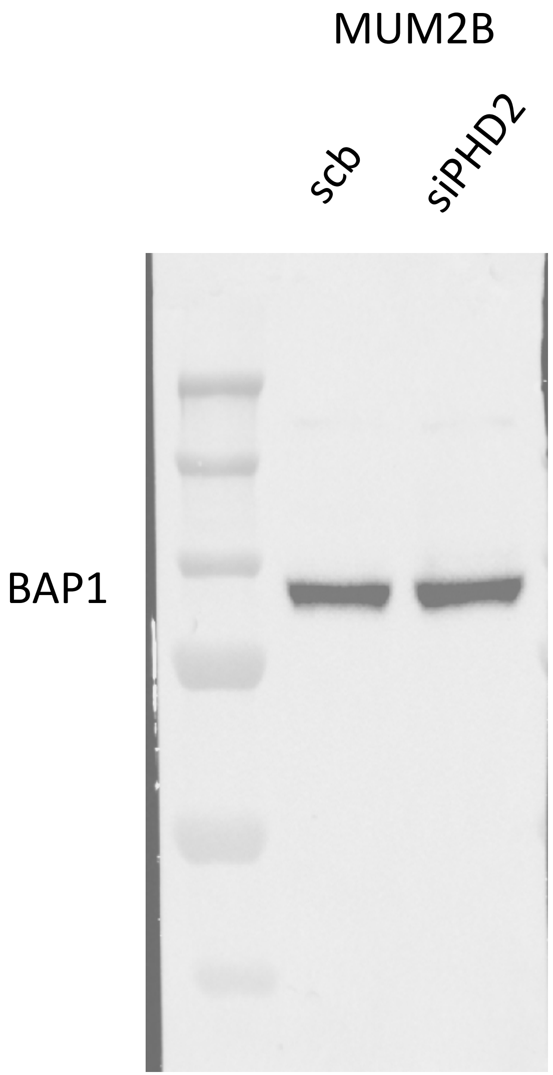

23/05/2024

MUM2B

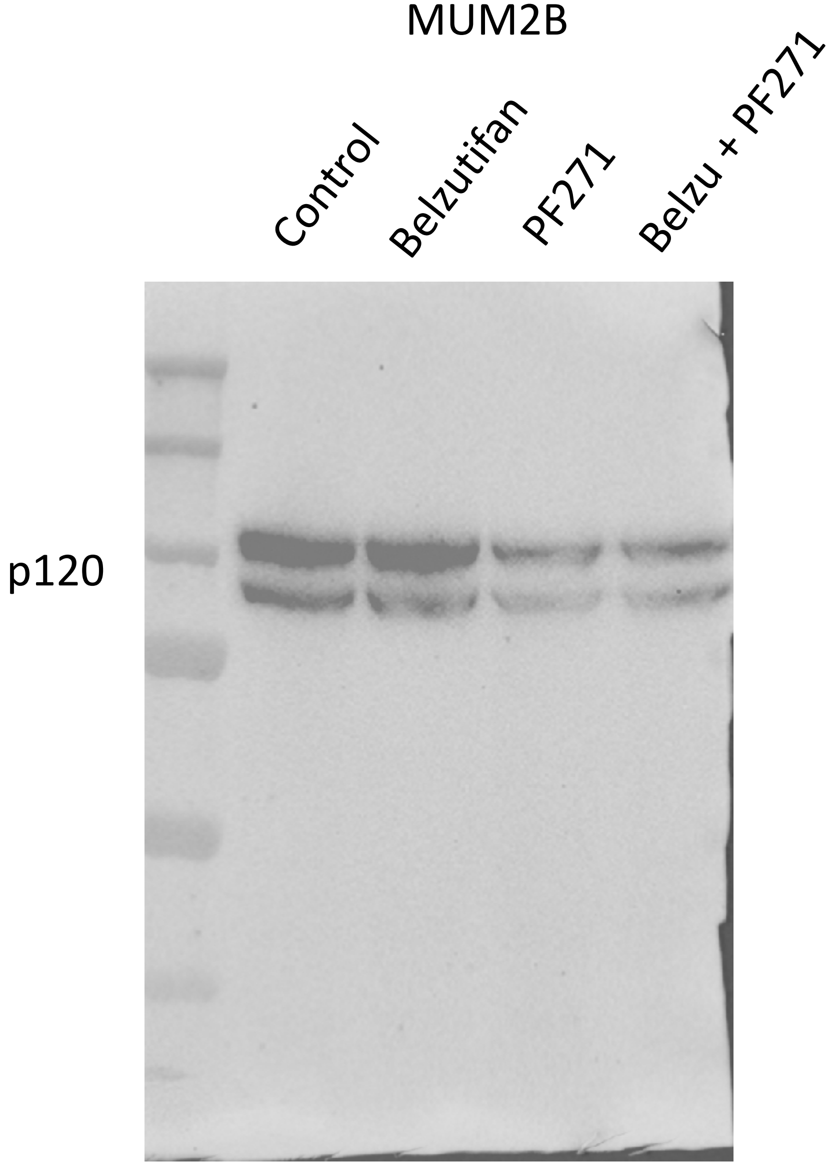

23/05/2024

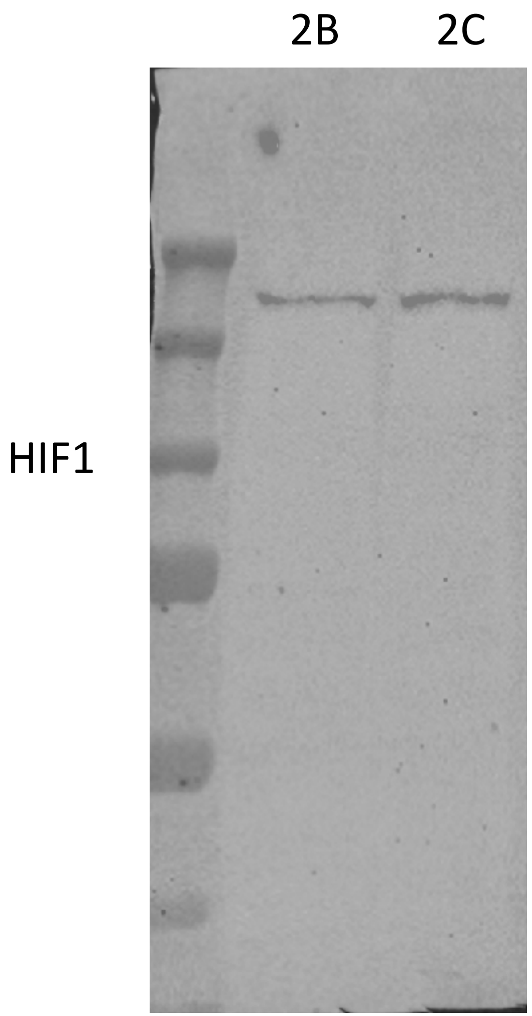

28/05/2024

MUM2B

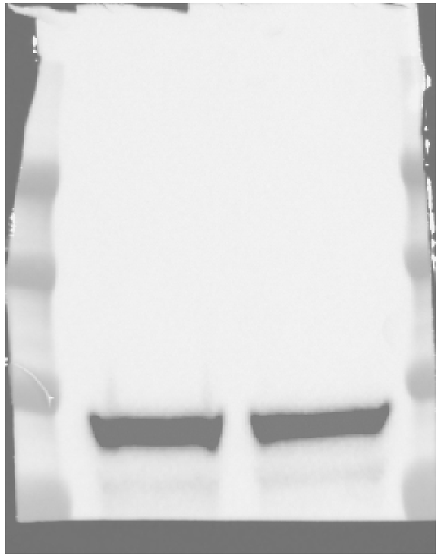

28/05/2024

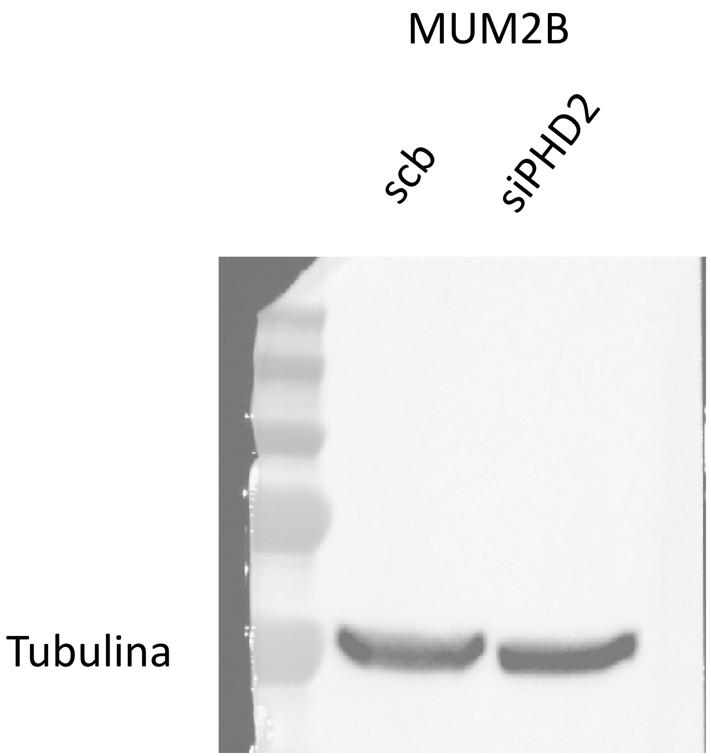

28/05/2024

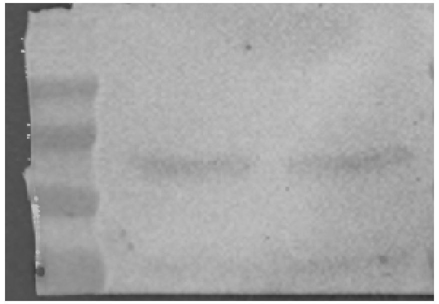

28/05/2024

MUM2B

scb siPHD2

VHL

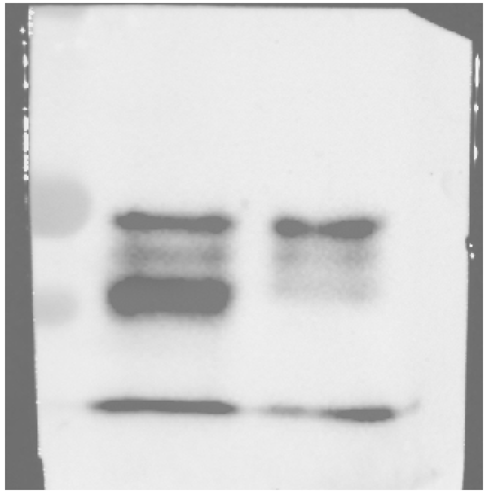

28/05/2024

MUM2B

scb siPHD2

Slug

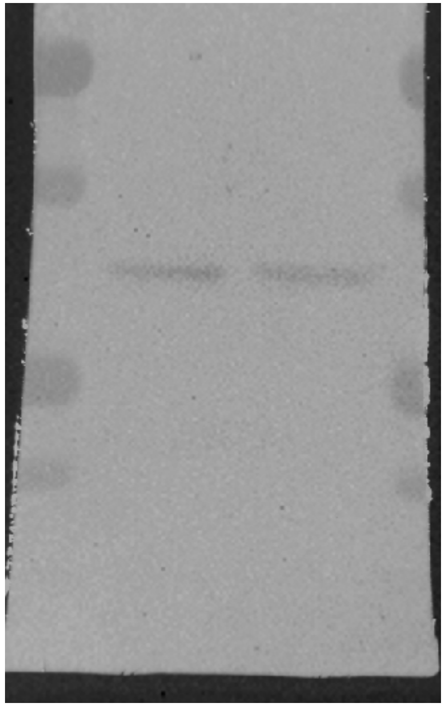

28/05/2024

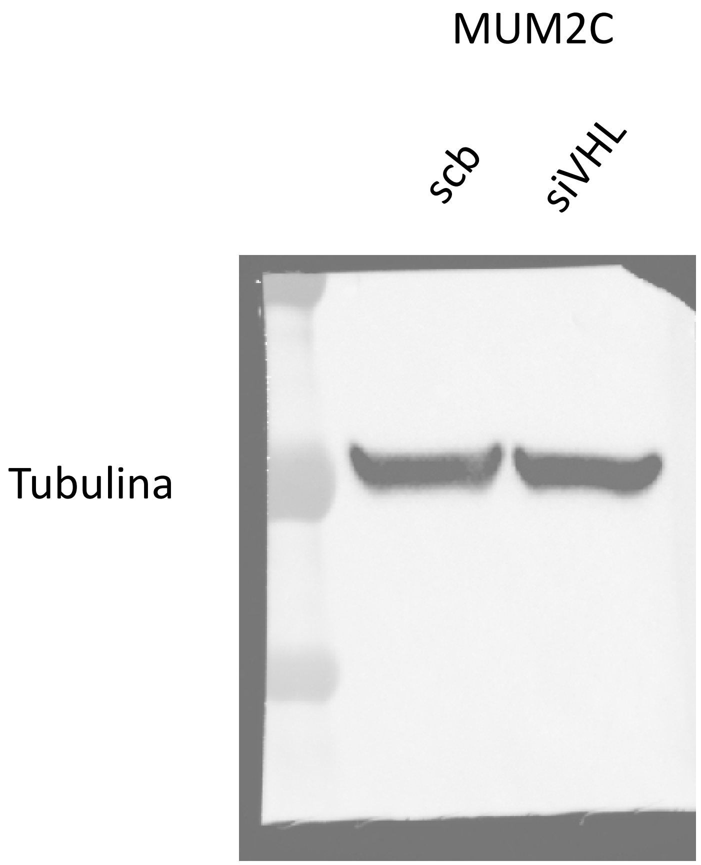

28/05/2024

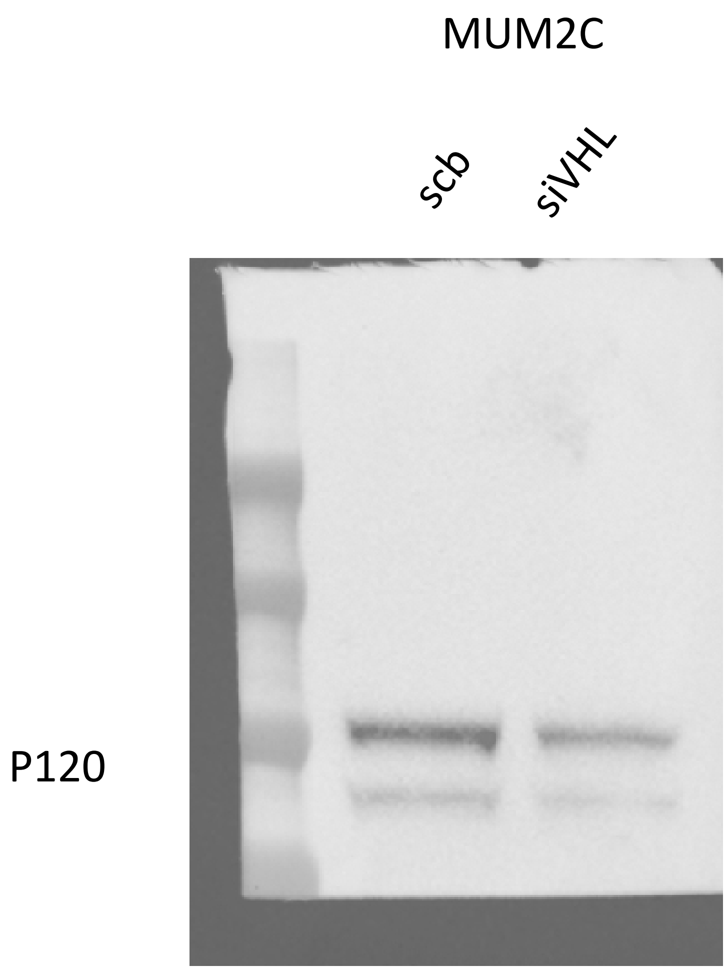

28/05/2024

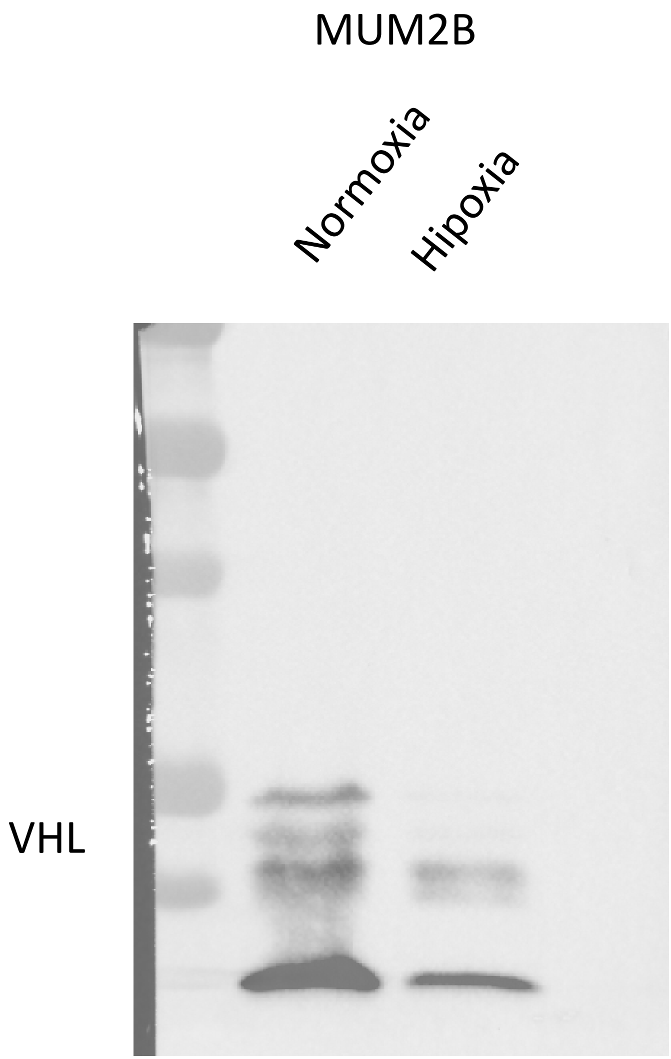

28/05/2024

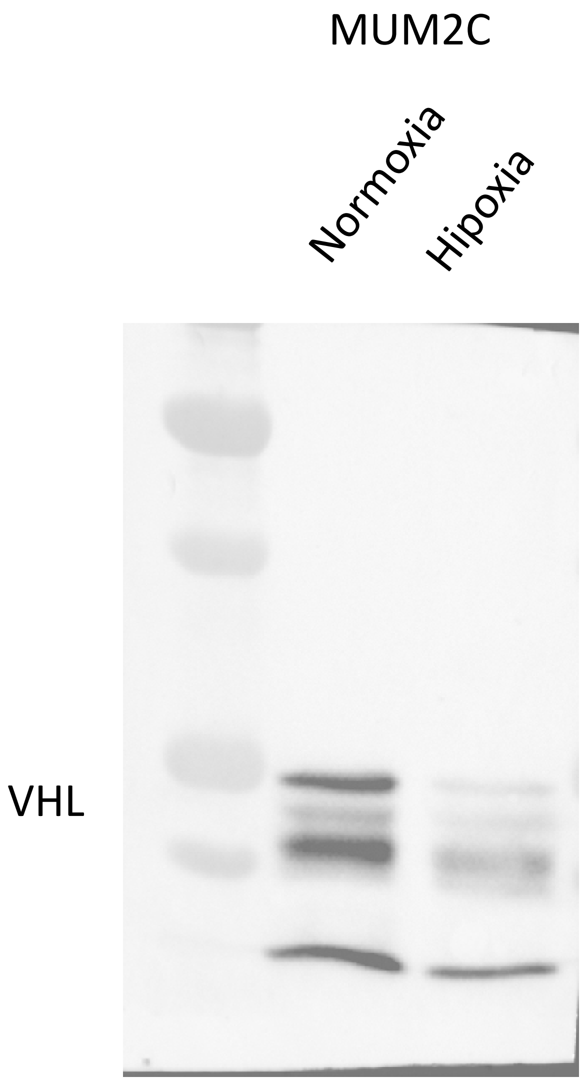

28/05/2024

MUM2C

Normoxia Hipoxia

BAP1

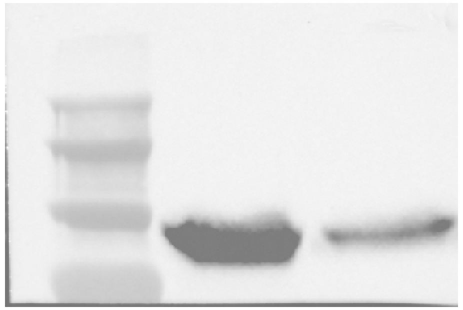

28/05/2024

MUM2C

Normoxia Hipoxia

ENG

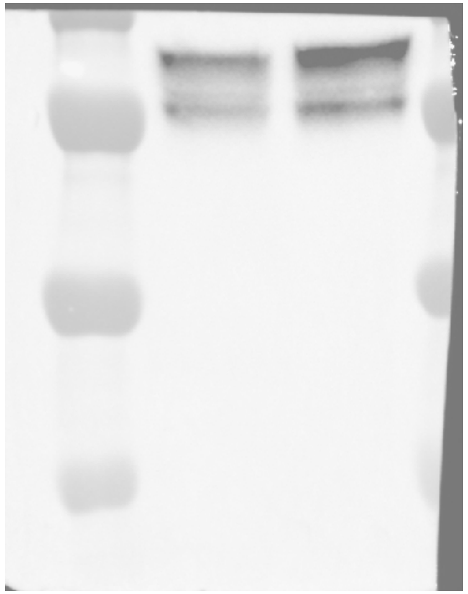

28/05/2024

MUM2C

Normoxia Hipoxia

HIF2

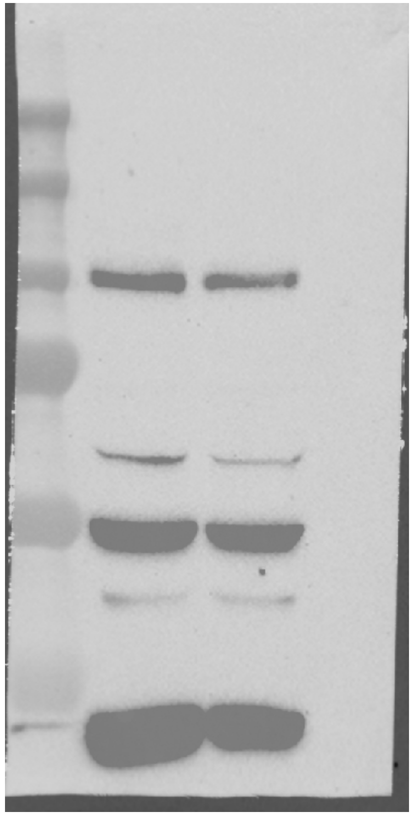

28/05/2024

MUM2B

Control Belzutifan PF271 Belzu + PF271

VHL

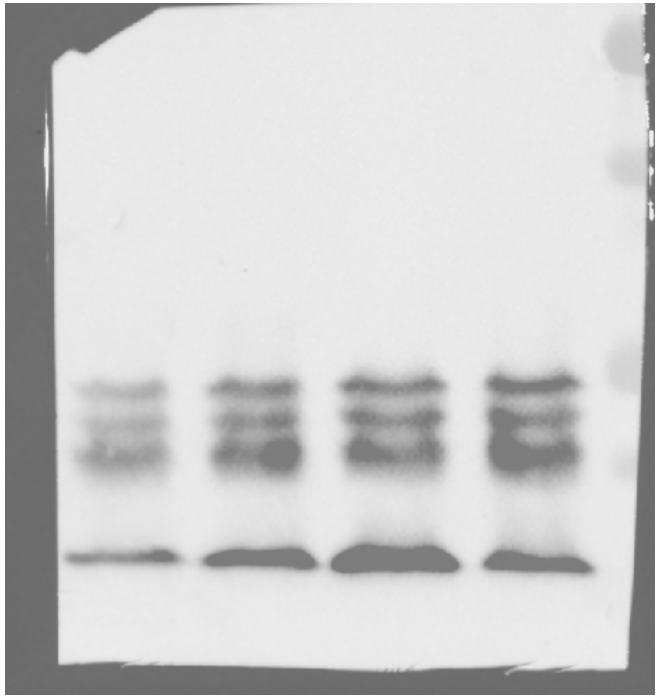

28/05/2024

MUM2B

Control Belzutifan PF271 Belzu + PF271

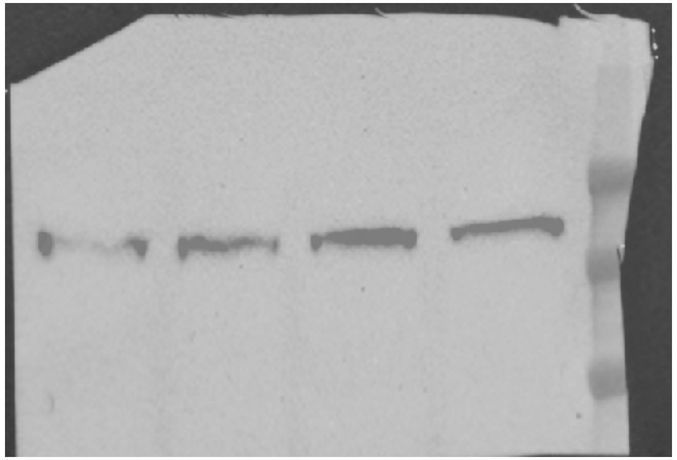

HIF1

28/05/2024

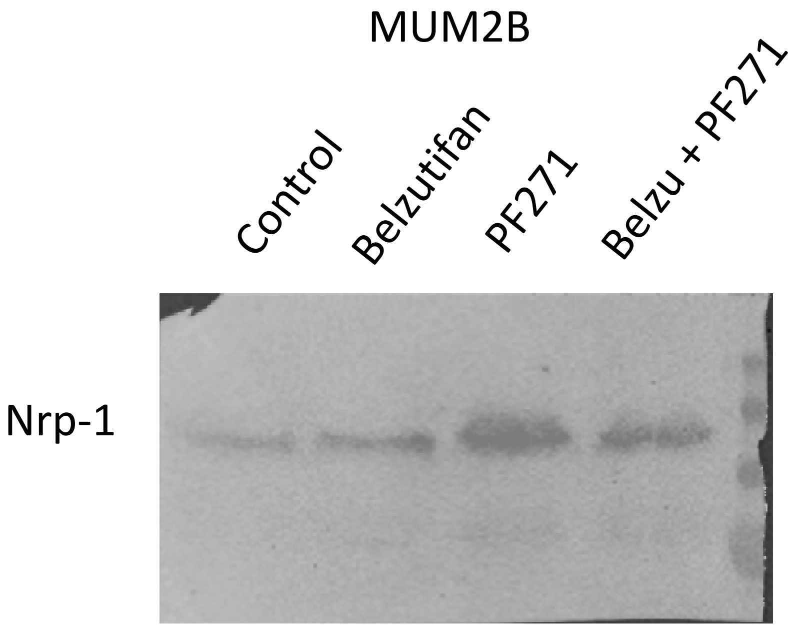

Supplement: Supplementary file 3 — WB original [file 41418_2025_1469_MOESM3_ESM.pdf]
